# Structure–activity relationship for calanthoside, a potential hair-growth stimulant with an indole 2-*S*-,3-*O*-bis-glucoside structure. Part 1: role of two glucoside moieties in promoting HFDPC proliferation

**DOI:** 10.1039/d6md00273k

**Published:** 2026-05-15

**Authors:** Riko Suzuki, Katsuki Takashima, Yoshiaki Manse, Haruto Nishikawa, Mina Ashidate, Shinsuke Marumoto, Fumihiro Ishikawa, Toshio Morikawa, Genzoh Tanabe

**Affiliations:** a Graduate School of Pharmacy, Kindai University 3-4-1 Kowakae Higashi-osaka Osaka 577-8502 Japan g-tanabe@phar.kindai.ac.jp; b Faculty of Pharmacy, Kindai University 3-4-1 Kowakae Higashi-osaka Osaka 577-8502 Japan; c Pharmaceutical Research and Technology Institute, Kindai University 3-4-1 Kowakae Higashi-osaka Osaka 577-8502 Japan morikawa@kindai.ac.jp; d Joint Research Centre, Kindai University 3-4-1 Kowakae Higashi-osaka Osaka 577-8502 Japan

## Abstract

The development of new and effective drugs for treating androgenetic alopecia (AGA) remains challenging. Minoxidil and finasteride, two drugs currently used to treat AGA, were originally developed for treating hypertension and prostate disease, respectively. Therefore, the pharmacological effects of these drugs may be double-edged in patients with AGA. Calanthoside (1) is an indole 2-*S*-,3-*O*-bis-glucoside isolated from the *Calanthe* genus. Compared to minoxidil, 1 induces significantly higher proliferation of human hair follicle dermal papilla cells (HFDPCs). This study aimed to identify the key structures responsible for the activity of 1. To this end, calanthoside derivatives were synthesised and evaluated. Analogues (9b, 9d–9f), in which the 3-*O*-glucoside moiety of 1 was replaced with different glycosides, exhibited minimal activity. In contrast, analogues (8c–8f), in which the 2-*S*-thioglucoside unit of 1 was substituted with various thioglycosides, demonstrated potent activity comparable to that of 1. These results suggest that 3-*O*-glucoside is an essential structural feature for activity. The 2-*S*-ethylated derivative (8g) exhibited a complete loss of activity. Similarly, compounds 10a and 10b, in which all the hydroxyl groups of the sugar residue of 1 were ester-protected, also exhibited a complete loss of activity. Therefore, highly polar sugar structures are required at the 2- and 3-positions. Collectively, the findings of this initial evaluation of the structure–activity relationship (SAR) provide valuable insights for expanding the chemical space for the future development of AGA treatments.

## Abstract 2

The development of new and effective drugs for treating androgenetic alopecia (AGA) remains challenging.

## Graphical abstract

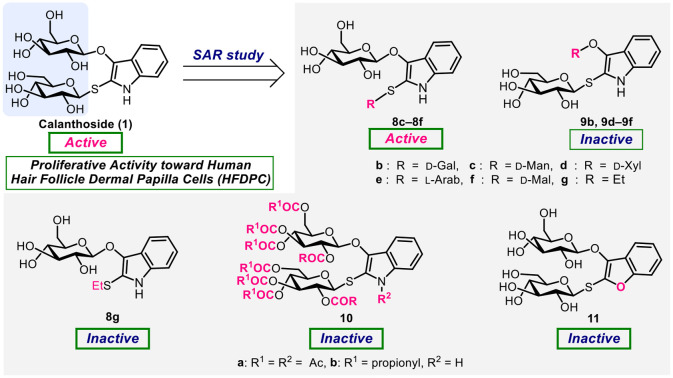

## Introduction

Androgenetic alopecia (AGA), also known as male pattern hair loss (MPHL) or female pattern hair loss (FPHL), is the most common form of hair loss worldwide. AGA affects up to 80% of men and 50% of women during their lifetime, and its prevalence increases with age.^1^ Hair loss can significantly impair patients' quality of life, reduce self-esteem, and elevate daily stress levels, potentially leading to psychological disorders.^2–4^ Currently, only two drugs, minoxidil and finasteride, are approved by the US Food and Drug Administration (FDA) for the treatment of AGA. However, both were originally developed for unrelated indications: minoxidil for hypertension and finasteride for prostate disorders.^5,6^ Consequently, these drugs can exert double-edged pharmacological actions in patients with AGA.^5,6^ Topical minoxidil is associated with localised side-effects such as scalp itching, rash, irritation, dryness, and dandruff.^7^ In some cases, its vasodilatory properties may also lead to systemic adverse effects, including headaches, dizziness, palpitations, and peripheral oedema.^8^ Studies indicate that finasteride, a 5α-reductase inhibitor, can cause sexual dysfunction in men, including reduced libido and erectile dysfunction.^9^ Moreover, its use in women is limited owing to potential teratogenicity.^10^ Thus, the development of new therapeutic agents for AGA remains an ongoing challenge.^11–13^ Identifying alternative lead compounds from natural sources has emerged as a promising strategy for addressing these issues.^14–17^

**Scheme 1:**
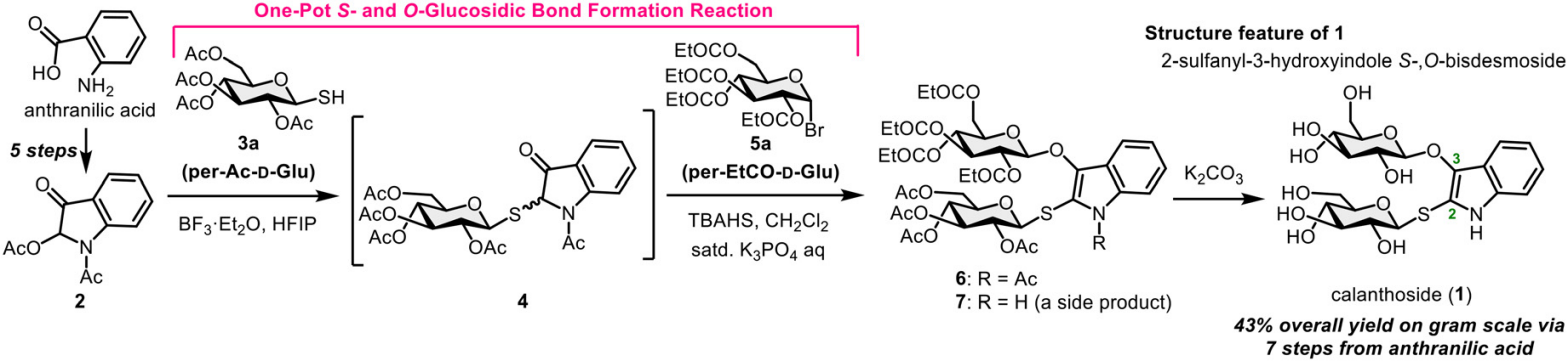
Overview of the synthetic route to calanthoside (1) using one-pot *S*-,*O*-glycosidic bond-formation reaction. TBAHS = tetrabutylammonium hydrogen sulfate.

**Fig. 1:**
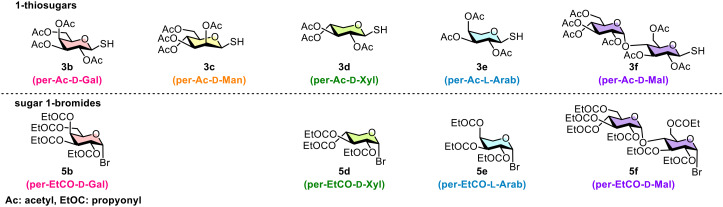
Structure of synthons for one-pot *S*-,*O*-glycosidic bond-formation reaction of 2.

**Fig. 2:**
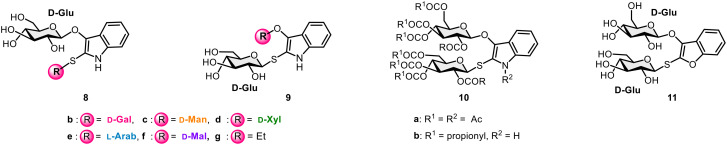
Designed calanthoside derivatives (8a–8e, 9a–9e, 10a, 10b and 11).

In 1998, Yoshikawa *et al.* isolated calanthoside (1), an indole alkaloid, from the plants *Calanthe discolor* LINDL. (known as “ebine” in Japanese) and *C. liukiuensis* SCHLTR.^18,19^ Compound 1 induces potent proliferation of human hair follicle dermal papilla cells (HFDPCs), exhibiting a level of activity superior to that of minoxidil.^20^ Moreover, studies have shown that its mechanism of action does not involve 5α-reductase inhibition,^20^ suggesting that 1 can prospectively serve as an alternative to finasteride for treating androgenetic alopecia (AGA) in both sexes. Structurally, compound (1) features a unique indole 2-*S*,3-*O*-bis-glucoside in which two d-glucose units are attached to the 2-sulfanyl-3-hydroxyindole framework *via S*- and *O*-glycosidic bonds. The novel architecture and attractive biological activity of 1 make it particularly intriguing. However, despite its promising profile, no synthetic route to 1 has been established, and no structure–activity relationship (SAR) studies have been conducted to date. Recently, we accomplished the first total synthesis of 1 on a preparative scale.^21^ Building on this achievement, a straightforward synthetic strategy (Scheme 1) was established for accessing structurally related analogues, either through replacement of the sugar-type intermediates (3a and 5a) used in the one-pot *S*,*O*-glucosidic bond-forming sequence (2 → 4 → 6) with alternative sugar derivatives (3b–3f, 5b, and 5d–5f; see Fig. 1), or through the use of isosteres of 2 in which an oxygen atom replaces the nitrogen atom. The structural modification of 1 represents a promising approach for discovering novel lead compounds for the treatment of AGA. In this study, we report the first evaluation of the SAR of 1. To examine the influence of structural variations of the sugar moiety on the biological activity, a series of analogues (8b–8f, 9b, and 9d–9f; see Fig. 2) was synthesised, in which the d-glucose moiety attached to either the sulfur or oxygen atom of 1 was replaced with different sugar units. In addition, analogue 8g, bearing an ethyl group instead of the d-glucose unit on the oxygen atom of 1, was prepared. Per-*O*-acylated derivatives (10a and 10b) were also synthesised to evaluate the role of the hydroxyl groups in the sugar moiety. The effects of the synthesised compounds on HFDPCs proliferation were evaluated. In addition to these biological evaluations, benzofuran (11) was synthesised and evaluated. Although 11 was virtually inactive, during its synthesis, a distinctly different mechanism of bond formation at the 2-position of intermediate 16b was revealed, compared to the S_N_1-type mechanism^21^ observed for intermediate 2 in the synthesis of compounds 1, 8b–8f, 9b, and 9d–9f, which is also discussed herein.

## Results and discussion

**Scheme 2:**
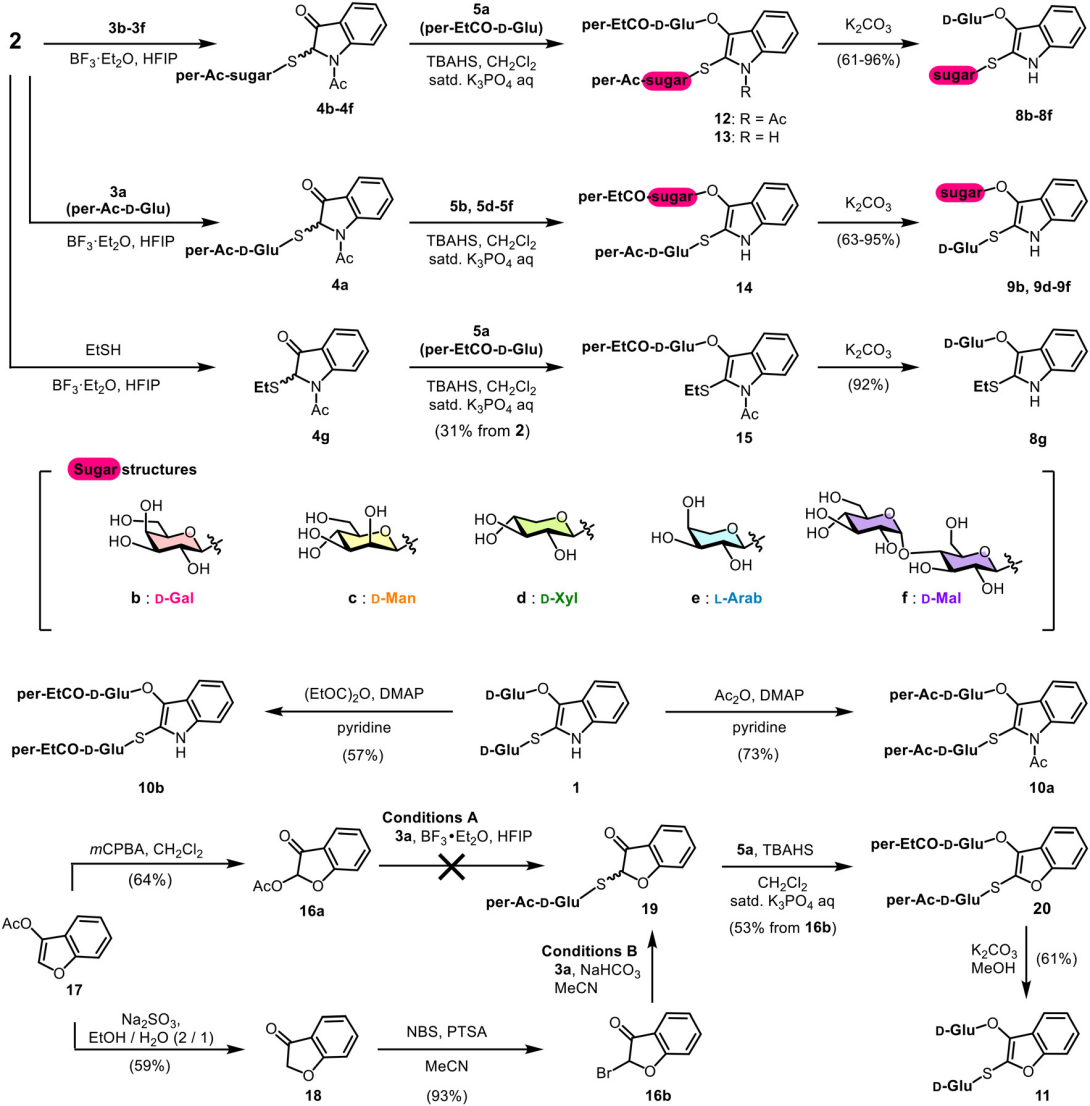
Synthesis of derivatives (8–11).

Following our previously reported method,^21^ indolinone 2, prepared from anthranilic acid, was treated sequentially with peracylated 1-thiosugars 3b–3f and peracylated glucose 1-bromide 5a to afford a mixture of *S*,*O*-bisdesmosides 12b–12f and their de-*N*-deacylated products 13b–13f in good yields. Without separating these two components, the acyl groups were subjected to methanolysis in the presence of K_2_CO_3_ to furnish 3-*O*-glucoside-type analogues 8b–8f in good yields. Conversely, when compound 2 was first reacted with peracylated 1-thioglucose 3a, followed by 3-*O*-glycosylation of the resulting 2-*S*-glucoside 4a with 5b, 5d, 5e, and 5f, the corresponding *S*,*O*-bisdesmosides 14b, 14d, 14e, and 14f were obtained in good yields. These compounds were then converted to the target analogues 9b, 9d, 9e, and 9f under methanolytic conditions similar to those used in the preparation of 8b–8f. In the positive-mode electrospray ionization (ESI) mass spectra, the quasimolecular ion peaks [M + Na]^+^ at *m*/*z* 512, 512, 482, 482, 674, 512, 482, 482, and 674 correspond to products 8b, 8c, 8d, 8e, 8f, 9b, 9d, 9e, and 9f, respectively, consistent with the proposed molecular structures. The ^1^H and ^13^C NMR spectra of these analogues were generally similar, except for differences in the signals arising from the hydrogen and carbon atoms of the sugar residues. For instance, the ^13^C NMR spectra of series 8 and 9 showed eight signals assignable to the carbons of the 2-sulfanyl-2-hydroxyindole ring in the range of *δ*_C_ 110–143 ppm. Additionally, signals arising from the anomeric carbons, supporting their *S*- and *O*-glycoside structures, appeared at *δ*_C_ 87 ppm and *δ*_C_ 105 ppm, respectively. Furthermore, the vicinal coupling constants (*J*_1,2_ = 9.0–9.7 Hz for the *S*-glucoside moiety and *J*_1,2_ = 7.2–7.9 Hz for the *O*-glucoside moiety) for the anomeric proton H-1 and the H-2 methine protons in these analogues (except for the signal of H-1 in the 2-*S*-mannoside structure of analogue 8c) indicated that both sugar units are linked *via* β-glycosidic bonds. The spectrum of compound 8c exhibited a signal of the anomeric proton H-1 at *δ*_H_ 4.98, with a small coupling constant (*J*_1,2_ ≈ 0 Hz), characteristic of the β-configuration of the anomeric carbon in the mannose unit. The β-stereochemistry at the anomeric centre was confirmed by nuclear Overhauser effect (NOE) correlations among the three axial protons (H-1, H-3, and H-5) of the mannose residue. Additionally, the related analogue 8g was synthesised *via* a three-step sequence comprising *S*-glucosylation with ethyl mercaptan, *O*-glucosidation of the resulting intermediate 4g, and deprotection of compound 15 under basic conditions. The ^13^C NMR spectrum of 8g was highly similar to those of 8b–8f, except for the observation of signals at *δ*_C_ 15.5 and 31.0 ppm, arising from the ethyl group carbons in 8g, which appeared in place of the sugar residues in 8b–8f. Calanthoside (1), pre-prepared in large quantities,^21^ was acylated with either acetic or propionic anhydride in pyridine in the presence of a catalytic amount of 4-dimethylaminopyridine (DMAP) to produce 10a and 10b, with yields of 73 and 57%, respectively (Scheme 2). In the ^13^C NMR spectra of 10a and 10b, the signals of the sugar carbons (C2, C3, C4, C6) shifted to a lower field compared to those of 1, providing strong evidence that the hydroxyl groups were fully acylated. The quasimolecular [M + Na]^+^ peaks observed at *m*/*z* 868 and 938 in the ESI mass spectra of 10a and 10b, respectively, are consistent with the molecular weights of the corresponding acylated compounds. Subsequently, benzofuran-type analogue 11 was synthesised as follows: Using a literature procedure,^22^ key intermediate 3-acetoxybenzofuran (17) was prepared from salicylic acid. After treatment of 17 with *m*-chloroperoxybenzoic acid (*m*CPBA), the resulting oxidation product 16a was subjected to the *S*-glycosidic bond-formation reaction under the same conditions used in the synthesis of 4a. Interestingly, the desired intermediate 19 was not observed, suggesting that the S_N_1-type mechanism involving electron donation from nitrogen, which proceeds with compound 4a,^23,24^ does not occur in the case of 16a. To overcome this limitation, compound 16b, in which the leaving group was changed from an acetate moiety to a bromine moiety, was synthesised from 17 in two steps and subsequently reacted with the thiolate anion generated from 3a under basic conditions to afford 19. The successful synthesis of this compound supports the conclusion that the reaction proceeds *via* an S_N_2-type mechanism. Compound 19 was found to be rather unstable and was, therefore, immediately *O*-glucosylated with 5a to afford the fully acylated analogue 20 in 53% yield *via* a two-step reaction from 16b. Finally, methanolysis of 20 furnished the desired product 11 in 61% yield. The spectra of compound 11 exhibited a quasimolecular ion [M + Na]^+^ peaks at *m*/*z* 905; and its molecular formula, C_20_H_50_O_20_SNa, was confirmed by high-resolution ESI mass spectrometry (HRESIMS). The ^13^C NMR spectrum of 11 closely match that of calanthoside (1), strongly suggesting that 11 is an isostere of 1 (Scheme 2).

With all thirteen target compounds (8b–8g, 9b, 9d–9f, 10a, 10b, and 11) in hand, their potency as activators of HFDPCs proliferation was examined and compared with that of 1 and minoxidil sulfate, as shown in Table 1. Although the biological activity of natural calanthoside (1) has been previously reported,^20^ its synthetic counterpart was revaluated in the present study as a reference standard. Compound 1 exhibited potent activity in promoting the proliferation of HFDPCs at concentrations ranging from 12.5 μM to 50 μM, with activity levels of 134.0% ± 5.2% to 139.6% ± 8.6% (entry 13) relative to that of the control. Although the proliferation-promoting effect of 1 was somewhat reduced at 100 μM compared with that at 50 μM, the compound still showed a favourable effect, with a proliferation rate of 125.0 ± 2.8% relative to that of the control. Furthermore, the efficacy of 1 was confirmed to be significantly higher than that of minoxidil sulfate (entry 15). Subsequently, proliferation activity tests were conducted on the series of 3-*O*-glucoside-type analogues 8b–8g. Unlike 8b and 8g, four analogues (8c, 8d, 8e, and 8f) were effective activators, exhibiting proliferation rates of 115.5% ± 5.4%, 120.8% ± 2.7%, 138.0% ± 2.7%, and 122.4% ± 3.5% *versus* that of the control at 100 μM, respectively (entries 2–5). Therefore, based on the results for compound 8d (entry 3), the hydroxymethyl group in the thioglucoside moiety of 1 is plausibly not essential for activity. Consistent with this structural insight, removal of this group from analogue 8b (entry 1) markedly increased its activity, yielding analogue 8e, which exhibited a proliferation-promoting effect of 138.0% ± 2.7% *versus* that of the control, surpassing that of 1 (125.0% ± 2.8% relative to the control). These findings suggest that in the absence of a hydroxymethyl group at the 5-position, the stereochemistry of the hydroxyl groups at the 2- or 4-position of the sugar moiety (axial or equatorial) is not a determining factor for proliferative activity. However, the activity of 8g was almost completely lost, suggesting that highly polar pyranoside moieties at the 2-position of the indole core are essential for maintaining the activity (entry 6). In contrast, among the 2-thioglucoside-type analogues (9b, 9d, 9e, and 9f), only 9e retained slight activity. The remaining compounds (9b, 9d, and 9f) showed no significant proliferative effect, even at a concentration of 100 μM (entries 7–10, 79.9% ± 1.8% to 101.1% ± 0.5% *versus* the control). These results indicate that the 3-*O*-glucoside moiety is requisite for the activity of this series. The per-acylated derivatives 10a and 10b exhibited lower inhibitory activities than the parent compound 1 (entries 10 and 11). Notably, 10a exhibited markedly reduced activity and clear toxicity towards HFDPCs (entry 11). These findings indicate that the polarity of the hydroxyl groups of the sugar moieties is essential for the biological activity of the compounds. Finally, compound 11 exhibited a markedly diminished effect on cell proliferation compared to 1 and also showed mild toxicity towards HFDPCs. This result suggests that substituting the nitrogen atom with an oxygen atom is an unfavourable structural modification.

**Table 1:** Effects of compounds 8–11 on the proliferation of HFDPCs

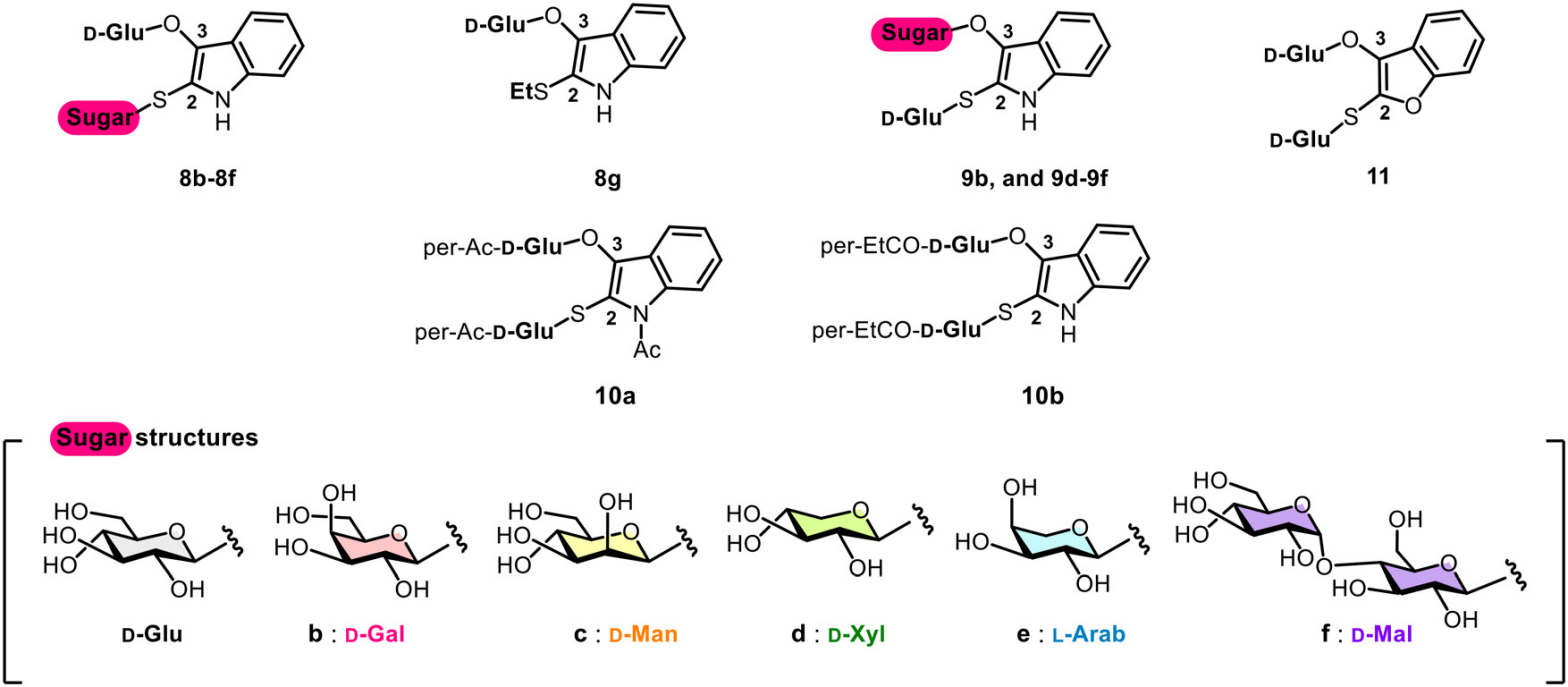						
Entry	Compounds	Relative activity level^a^ (% of control)				
		0 μM	12.5 μM	25 μM	50 μM	100 μM
1	8b (2S-**Gal**;3O-**Glu**)	100.0 ± 6.9	89.1 ± 2.6	94.9 ± 6.4	95.9 ± 4.0	99.5 ± 1.9
2	8c (2S-**Man**;3O-**Glu**)	100.0 ± 1.3	103.6 ± 5.2	110.5 ± 1.2	119.0 ± 0.7**	115.5 ± 5.4*
3	8d (2S-**Xyl**;3O-**Glu**)	100.0 ± 6.3	105.6 ± 5.4	106.2 ± 3.9	105.3 ± 4.3	120.8 ± 2.7*
4	8e (2S-**Arab**;3O-**Glu**)	100.0 ± 4.6	104.4 ± 3.4	108.1 ± 0.8	134.9 ± 8.8**	138.0 ± 2.7**
5	8f (2S-**Mal**;3O-**Glu**)	100.0 ± 1.8	101.8 ± 0.3	103.1 ± 2.7	109.6 ± 6.1	122.4 ± 3.5**
6	8g (2S-Et;3O-**Glu**)	100.0 ± 4.5	87.7 ± 6.6	99.4 ± 6.7	100.6 ± 6.5	106.1 ± 2.3
7	9b (2S-**Glu**;3O-**Gal**)	100.0 ± 4.4	96.2 ± 6.4	97.5 ± 5.2	96.6 ± 4.5	98.5 ± 4.3
8	9d (2S-**Glu**;3O-**Xyl**)	100.0 ± 10.4	100.4 ± 9.5	106.1 ± 9.9	100.4 ± 8.6	101.1 ± 0.5
9	9e (2S-**Glu**;3O-**Arab**)	100.0 ± 16.3	108.0 ± 19.7	117.7 ± 15.3	111.7 ± 14.3	105.5 ± 2.8
10	9f (2S-**Glu**;3O-**Mal**)	100.0 ± 4.8	101.4 ± 8.7	94.3 ± 3.8	95.0 ± 3.3	79.9 ± 1.8
11	10a (2S-Ac-**Glu**;3O-Ac-**Glu**)	100.0 ± 7.2	76.6 ± 4.8**	50.6 ± 3.3**	50.9 ± 0.3**	66.4 ± 1.5**
12	10b (2S-Pro-**Glu**;3O-Pro-**Glu**)	100.0 ± 5.5	98.1 ± 5.4	97.3 ± 4.7	119.0 ± 4.5	108.6 ± 3.4
13	Calanthoside (1, synthetic)^b^	100.0 ± 2.9	134.0 ± 5.2**	138.9 ± 4.1**	139.6 ± 8.6**	125.2 ± 2.8
14	11	100.0 ± 7.4	89.0 ± 5.7	87.5 ± 4.6	99.1 ± 17.3	90.4 ± 3.3**
15	Minoxidil sulfate^c^	100.0 ± 1.7	108.3 ± 5.5	101.5 ± 4.4	107.5 ± 2.7	112.4 ± 3.3

a Each value represents the mean ± S.E.M. (*N* = 3). Significantly different from the control, *p < 0.05, **p < 0.01.

b The activity of natural calanthoside (1) had been previously reported.^20^ However, to compare the activity of candidates 8–11, we re-examined the activity of the synthetic calanthoside (1).

c Minoxidil sulfate was purchased from FUJIFILM Wako Pure Chemical Corporation (Osaka, Japan).

## Conclusions

Calanthoside (1) is an indole 2-*S*,3-*O*-bis-glucoside found in traditional medicinal plants. Because of its potential to promote hair growth, it is considered a promising lead compound for the development of novel pharmaceuticals to treat androgenetic alopecia (AGA). This study investigated the relationship between the two glucoside structures and the proliferative activity of HFDPCs as an initial exploration of the structure–activity relationship. Compounds 8c, 8d, 8e, and 8f (excluding 8b) exhibit strong proliferation-promoting effects. In contrast, the latter group (9b, 9d, 9e, and 9f) shows almost no activity. These findings indicate that the 3-*O*-glucoside structure is essential for promoting proliferation. Furthermore, the inactivity of the 2-*S*-ethylated derivative 8g, which is related to 8b–8f, suggests that introducing a hydrophobic substituent at the 2-position of the sulfur atom is undesirable. Thus, it was determined that a highly polar pyranoside unit at the 2-*S* position of the indole core is required for achieving activity. Additionally, no activity was detected for the peracylated analogues 10a and 10b, indicating that the polarity of the sugar moiety is crucial for the manifestation of activity. Taken together, these findings provide a foundation for the further development of calanthoside derivatives. In our laboratory, structure–activity relationship studies of calanthoside (1) are currently being advanced, with particular emphasis on substitutions on the indole ring. In addition, further studies including toxicity profiling, transcriptomic analysis, and combination studies with reference compounds such as minoxidil are planned in order to further elucidate the pharmacological properties of these derivatives.

## Experimental section

### Chemistry

Optical rotations were determined with a digital polarimeter. IR spectra were recorded on a FT-IR spectrophotometer. NMR spectra were recorded on FT-NMR spectrometers (^1^H, 800 MHz; ^13^C, 200 MHz). Tetramethylsilane (TMS) was used as an internal standard for ^1^H NMR measurements in CDCl_3_, whereas ^13^C NMR measurements utilized the solvent signal (77.0 ppm) of CDCl_3_ for this purpose. When CD_3_OD or DMSO-*d*_6_ was used for the measurement of ^1^H and ^13^C NMR spectra, solvent signals [CD_3_OD (*δ*_H_ 3.30 ppm and *δ*_C_ 49.0 ppm); DMSO-*d*_6_ (*δ*_H_ 2.49 ppm and *δ*_C_ 39.7 ppm)] were used as standards for ^1^H and ^13^C NMR measurements. Sodium 2,2-dimethyl-2-silapentane-5-sulfonate (DSS) was used as an external standard for the measurement of ^1^H and ^13^C NMR spectra in D_2_O. 1D NMR peak assignments were confirmed by COSY, HSQC, and HMBC spectra. High-resolution mass spectra were recorded on an Orbitrap mass spectrometer (ESI). All organic extracts were dried over anhydrous Na_2_SO_4_ prior to evaporation. Column chromatography was performed over silica gel (45–106 μm). Mixtures of each 12b and 13b, 12c and 13c, 12d and 13d, 12e and 13e, and 12f and 13f, as well as compounds 14b, 14d, 14e, and 14f, were prepared according to the literature procedures^21^ prior to methanolysis, which was then carried out as described below.

#### 3-(β-d-Glucopyranosyloxy)-1*H*-indol-2-yl 1-thio-β-d-galactopyranoside (8b)

The mixture of 12b (207 mg, 0.22 mmol), and 13b (198 mg, 0.22 mmol) was treated with K_2_CO_3_ (6 mg, 0.044 mmol) in MeOH (5 mL) at room temperature (rt) for 4 h. After the reaction mixture was condensed *in vacuo*, the residue was purified by column chromatography (CHCl_3_/MeOH = 20/1) to give the title compound (8b, 179 mg, 0.37 mmol, 83%) as a pale yellow microcrystalline solid. Mp 138–139 °C. [*α*]^25^_D_ −10.0 (*c* = 1.0, MeOH). IR (KBr): 3366, 2920, 2853, 1449, 1344, 1240, 1134, 1074, 1011, 856, 824 cm^−1^. ^1^H NMR (800 MHz, D_2_O) *δ*: 3.28 (1H, ddd, *J* = 9.6, 5.1, 2.4, OGlu H-5), 3.44 (1H, dd, *J* = 9.3, 9.3, OGlu H-4), 3.46 (1H, dd, *J* = 9.7, 9.7, SGal H-2), 3.48 (1H, dd, *J* = 9.6, 9.6, OGlu H-3), 3.51–3.54 (2H, m, SGal H-3 and SGal H-5), 3.52 (1H, dd, *J* = 7.9, 9.6, OGlu H-2), 3.60 (1H, dd, *J* = 11.7, 4.3, SGal H-6a), 3.66 (1H, dd, *J* = 12.5, 5.1, OGlu H-6a), 3.67 (1H, dd, *J* = 11.7, 7.9, SGal H-6b), 3.74 (1H, dd, *J* = 12.5, 2.4, OGlu H-6b), 3.82 (1H, dd, *J* = 3.3, 1.0, SGal H-4), 4.58 (1H, d, *J* = 9.7, SGal H-1), 5.02 (1H, d, *J* = 7.9, OGlu H-1), 7.03 (1H, ddd, *J* = 8.0, 6.9, 1.0, H-5), 7.14 (1H, ddd, *J* = 8.2, 6.9, 1.2, H-6), 7.28 (1H, br dd-like *J* = *ca.* 8.2, 1.0, H-7), 7.62 (1H, dd, *J* = 8.0, 1.2, H-4). ^13^C NMR (200 MHz, D_2_O) *δ*: 60.5 (OGlu C-6), 61.2 (SGal C-6), 68.8 (SGal C-4), 69.3 (OGlu C-4), 69.6 (SGal C-2), 73.4 (OGlu C-2), 73.9 (SGal C-3), 75.6 (OGlu C-3), 76.2 (SGal C-5), 79.3 (OGlu C-5), 88.3 (SGal C-1), 104.7 (OGlu C-1), 111.9 (C-7), 113.4 (C-2), 117.9 (C-4), 120.0 (C-3a), 120.1 (C-5), 123.9 (C-6), 134.5 (C-7a), 139.0 (C-3). HRMS (ESI) *m*/*z*: [M+Na]^+^ Calcd for C_20_H_27_NNaO_11_S 512.1197; found 512.1197.

#### 3-(β-d-Glucopyranosyloxy)-1*H*-indol-2-yl 1-thio-β-d-mannopyranoside (8c)

Following the method similar to that used for the preparation of 8b, the mixture of 12c (143 mg, 0.16 mmol) and 13c (137 mg, 0.16 mmol) was treated with K_2_CO_3_ in MeOH to give the title compound (8c, 139 mg, 0.28 mmol, 88%) as a pale yellow microcrystalline solid. Mp 154–155 °C. [*α*]^25^_D_ −12.0 (*c* = 1.0, MeOH). IR (KBr): 3632, 2920, 2881, 1449, 1343, 1240, 1097, 1068 cm^−1^. ^1^H NMR (800 MHz, D_2_O) *δ*: 3.27 (1H, dd, *J* = 9.4, 6.5, 2.4, SMan H-5), 3.36 (1H, ddd, *J* = 9.4, 5.1, 2.3, OGlu H-5), 3.54 (1H, dd, *J* = 9.4, 9.4, OGlu H-4), 3.59 (1H, dd, *J* = 9.2, 9.2, OGlu H-3), 3.60–3.66 (3H, m, OGlu H-2, SMan H-3 and H-4), 3.73 (1H, dd, *J* = 12.4, 6.5, SMan H-6a), 3.75 (1H, dd, *J* = 12.4, 5.1, OGlu H-6a), 3.82 (1H, dd, *J* = 12.4, 2.3, OGlu H-6b), 3.84 (1H, dd, *J* = 12.4, 2.4, SMan H-6b), 4.22 (1H, d, *J* = 2.4, SMan H-2), 4.98 (br s-like, Man H-1), 5.00 (1H, d, *J* = 7.9, OGlu H-1), 7.13 (1H, ddd, *J* = 7.9, 6.9, 1.0, H-5), 7.25 (1H, ddd, *J* = 8.2, 6.9, 1.2, H-6), 7.39 (1H, dd, *J* = 8.2, 1.0, H-7), 7.72 (1H, dd, *J* = 6.9, 1.0, H-4). ^13^C-NMR (200 MHz, D_2_O) *δ*: 60.4 (SMan C-6), 61.0 (OGlu C-6), 66.5 (SMan C-4), 69.2 (OGlu C-4), 71.6 (SMan C-2), 73.3 (OGlu C-2), 73.6 (SMan C-3), 75.6 (OGlu C-3), 76.1 (SMan C-5), 80.3 (OGlu C-5), 87.5 (C-1 SMan), 104.7 (OGlu C-1), 111.7 (C-7), 114.9 (C-2), 117.7 (C-4), 119.7 (C-3a), 119.9 (C-5), 123.7 (C-6), 134.3 (C-7a), 138.2 (C-3). HRMS (ESI) *m*/*z*: [M + Na]^+^ Calcd for C_20_H_27_NNaO_11_S 512.1195; found 512.1197.

#### 3-(β-d-Glucopyranosyloxy)-1*H*-indol-2-yl 1-thio-β-d-xylopyranoside (8d)

Following the method similar to that used for the preparation of 8b, the mixture of 12d (51 mg, 60 μmol) and 13d (49 mg, 60 μmol) was treated with K_2_CO_3_ in MeOH to give the title compound (8d, 53 mg, 0.115 mmol, 96%) as a pale yellow microcrystalline solid. Mp 170–171 °C. [*α*]^25^_D_ −6.6 (*c* = 1.0, MeOH). IR (KBr): 3385, 2922, 1740, 1653, 1341, 1238, 1098, 1051, 1009, 897 cm^−1^. ^1^H NMR (800 MHz, D_2_O) *δ*: 3.13–3.17 (2H, m, SXyl H-2 and H-5a), 3.27 (1H, ddd, *J* = 9.5, 5.0, 2.3, OGlu H-5), 3.34 (1H, dd, *J* = 9.0, 9.0, SXyl H-3), 3.41–3.50 (3H, m, OGlu H-3, H-4 and SXyl H-4), 3.54 (1H, dd, *J* = 9.2, 7.9, OGlu H-2), 3.66 (1H, dd, *J* = 12.4, 5.0, OGlu H-6a), 3.74 (1H, dd, *J* = 12.4, 2.3, OGlu H-6b), 3.90 (1H, dd, *J* = 11.5, 5.3, SXyl H-5b), 4.47 (1H, d, *J* = 9.5, SXyl H-1), 4.90 (1H, d, *J* = 7.9, OGlu H-1), 7.00 (1H, ddd, *J* = 8.1, 7.0, 0.9, H-5), 7.27 (1H, ddd, *J* = 8.3, 7.0, 1.2, H-6), 7.35 (1H, br d-like, *J* = *ca.* 8.3, H-7), 7.69 (1H, br d-like, *J* = *ca.* 8.1, H-4). ^13^C-NMR (200 MHz, D_2_O) *δ*: 60.5 (OGlu C-6), 69.0(2C)/69.3 (OGlu C-4, SGlu C-4 and C-5), 72.0 (SXyl H-2), 73.4 (OGlu H-2), 75.7 (OGlu H-3), 76.3 (OGlu H-5), 77.1 (SXyl C-3), 87.9 (SXyl C-1), 104.6 (OGlu C-1), 112.0 (C-7), 112.6 (C-2), 117.9 (C-4), 119.6 (C-3a), 120.1 (C-5), 124.0 (C-6), 134.6 (C-7a), 139.5 (C-3). HRMS (ESI) *m*/*z*: [M + Na]^+^ Calcd for C_19_H_25_NNaO_10_S 482.1091; found 482.1091.

#### 3-(β-d-Glucopyranosyloxy)-1*H*-indol-2-yl 1-thio-β-l-arabinopyranoside (8e)

Following the method similar to that used for the preparation of 8b, the mixture of 12e (94 mg, 0.110 mmol) and 13e (87 mg, 0.108 mmol) was treated with K_2_CO_3_ in MeOH to give the title compound (8e, 84 mg, 0.183 mmol, 84%) as a pale yellow microcrystalline solid. Mp 150–151 °C. [*α*]^25^_D_ −23.2 (*c* = 1.0, MeOH). IR (KBr): 3524, 2922, 2855, 1701, 1458, 1240, 1076 cm^−1^. ^1^H NMR (800 MHz,D_2_O) *δ*: 3.34 (1H, ddd, *J* = 7.2, 4.9, 2.2, OGlu H-5), 3.36–3.58 (3H, m, OGlu H-4, SArab H-3 and H-5a), 3.60–3.65 (3H, m, OGlu H-2 and H-3, SArab H-2), 3.75 (1H, dd, *J* = 12.5, 5.0, OGlu H-6a), 3.82 (1H, dd, *J* = 12.5, 2.2, OGlu H-6b), 3.91–3.99 (2H, m, SArab H-4 and H-5b), 4.56 (1H, d, *J* = 9.0, SArab H-1), 4.98 (1H, d, *J* = 7.9, OGlu H-1), 7.12 (1H, dd, *J* = 7.8, 7.8, H-5), 7.23 (1H, dd, *J* = 7.8, 7.8, H-6), 7.37 (1H, d, *J* = 7.8, H-7), 7.71 (1H, d, *J* = 7.8, H-4). ^13^C NMR (200 MHz, D_2_O) *δ*: 60.3 (OGlu C-6), 68.2 (SArab C-4), 69.2 (OGlu C-4), 69.5 (SArab C-5), 69.6 (SArab C-2), 73.0 (SArab C-3), 73.3 (OGlu C-2), 75.5 (OGlu C-3), 76.1 (OGlu C-5), 88.3 (SArab C-1), 104.6 (OGlu C-1), 111.8 (C-7), 113.3 (C-2), 117.7 (C-4), 119.5 (C-3a), 119.9 (C-6), 123.8 (C-5), 134.4 (C-7a), 139.0 (C-3). HRMS (ESI) *m*/*z*: [M + Na]^+^ Calcd for C_19_H_25_NNaO_10_S 482.1091; found 482.1092.

#### 3-(β-d-Glucopyranosyloxy)-1*H*-indol-2-yl 1-thio-β-d-maltopyranoside (8f)

Following the method similar to that used for the preparation of 8b, the mixture of 12f (51 mg, 42 μmol) and 13f (49 mg, 41 μmol) was treated with K_2_CO_3_ in MeOH to give the title compound (8f, 34 mg, 51 μmol, 61%) as a pale yellow microcrystalline solid. Mp 150–151 °C. [*α*]^19^_D_ +3.6 (*c* = 1.0, MeOH). IR (KBr): 3395, 2920, 2888, 1616, 1449, 1342, 1240, 1148, 1059, 1032, 916 cm^−1^. ^1^H NMR (800 MHz, D_2_O) *δ*: 3.28 (1H, dd, *J* = 9.4, 9.4), 3.40–3.43 (2H, m), 3.48 (1H, ddd, *J* = 9.9, 5.0, 2.3), 3.55–3.58 (3H, m), 3.61 (1H, dd, *J* = 9.2, 9.2), 3.65–3.71 (3H, m), 3.74–3.81 (4H, m), 3.83–3.90 (3H, m), 4.60 (1H, d, *J* = 9.7, SMal H-1), 5.03 (1H, d, *J* = 7.8, OGlu H-1), 5.33 (1H, d, *J* = 4.0, SMal H-1′), 7.14 (1H, br dd-like, *J* = *ca.* 8.2, 6.9, H-5), 7.26 (1H, dd, *J* = 8.4, 6.9, 1.2, H-6), 7.41 (1H, br d-like, *J* = *ca.* 8.4, H-7), 7.76 (1H, dd, *J* = 8.2, 1.2, H-4). ^13^C NMR (200 MHz, D_2_O) *δ*: 60.5, 60.6, 60.7, 69.3, 69.4, 71.8, 71.9, 72.8, 72.9, 73.4, 75.6, 76.2, 76.6, 77.5, 78.6, 87.1 (SMal C-1), 99.8 (OGlu C-1), 104.6 (OMal C-1′), 111.9 (C-7), 112.5 (C-2), 117.9 (C-4), 119.5 (C-3a), 120.1 (C-5), 124.0 (C-6), 134.6 (C-7a), 139.5 (C-3). HRMS (ESI) *m*/*z*: [M + Na]^+^ Calcd for C_26_H_37_NNaO_16_S 674.1725; found 674.1726.

#### 3-(β-d-Galactopyranosyloxy)-1*H*-indol-2-yl 1-thio-β-d-glucopyranoside (9b)

Following the method similar to that used for the preparation of 8b, 14b (150 mg, 0.170 mmol) was treated with K_2_CO_3_ in MeOH to give the title compound (9b, 83 mg, 0.155 mmol, 95%) as a pale red microcrystalline solid. Mp 159–160 °C. [*α*]^25^_D_ +83.1 (*c* = 1.0, MeOH). IR (KBr): 3337, 2930, 2859, 1748, 1715, 1636, 1506, 1429, 1340, 1240, 1067, 1048, 1026 cm^−1^. ^1^H NMR (800 MHz, CD_3_OD) *δ*: 3.10 (1H, dd, *J* = 9.5, 8.9, SGlu H-2), 3.18 (1H, dd, *J* = 8.9, 8.9, SGlu H-4), 3.29 (1H, ddd, *J* = 8.9, 5.7, 2.4, SGlu H-5), 3.37 (1H, dd, *J* = 8.9, 8.9, SGlu H-3), 3.56 (1H, ddd, *J* = 6.2, 6.2, 1.2, OGal H-5), 3.58 (1H, dd, *J* = 7.8, 3.4, OGal H-3), 3.66 (1H, dd, *J* = 12.0, 5.7, SGlu H-6a), 3.76–3.77 (2H, br m, OGal H-6), 3.86 (1H, dd, *J* = 12.0, 2.4, SGlu H-6b), 3.87 (1H, dd, *J* = 8.9, 7.8, OGal H-2), 3.89 (1H, dd, *J* = 3.4, 1.2, OGal H-4), 4.41 (1H, d, *J* = 9.5, SGlu H-1), 4.86 (1H, d, *J* = 7.8, OGal H-1), 6.98 (1H, ddd, *J* = 8.0, 7.0, 0.9, H-5), 7.11 (1H, ddd, *J* = 8.2, 7.0, 1.2, H-6), 7.24 (1H, dd, *J* = 8.2, 0.9, H-7), 7.78 (1H, dd, *J* = 8.0, 1.2, H-4). ^13^C NMR (200 MHz, CD_3_OD) *δ*: 62.2 (OGal C-6), 62.6 (SGlu C-6), 70.2 (OGal C-4), 71.1 (SGlu C-4), 72.9 (OGal C-2), 73.6 (SGlu C-2), 74.8 (OGal C-3), 76.8 (OGal C-5), 79.1 (SGlu C-3), 81.9 (SGlu C-5), 89.0 (SGlu C-1), 107.2 (OGal C-1), 112.0 (C-7), 112.9 (C-2), 119.7 (C-4), 120.2 (C-5), 121.7 (C-3a), 124.2 (C-6), 136.3 (C-7a), 142.3 (C-3). HRMS (ESI) *m*/*z*: [M + Na]^+^ Calcd for C_20_H_27_NNaO_11_S 512.1197; found 512.1191.

#### 3-(β-d-Xylopyranosyloxy)-1*H*-indol-2-yl 1-thio-β-d-glucopyranoside (9d)

Following the method similar to that used for the preparation of 8b, 14d (180 mg, 0.23 mmol) was treated with K_2_CO_3_ in MeOH to give the title compound (9d, 87 mg, 0.19 mmol, 83%) as a pale yellow microcrystalline solid. Mp 154–155 °C. [*α*]^25^_D_ −6.7 (*c* = 1.0, MeOH). IR (KBr): 3337, 2930, 2859, 1748, 1715, 1636, 1506, 1429, 1340, 1240, 1067 cm^−1^. ^1^H NMR (800 MHz, CD_3_OD) *δ*: 3.05 (1H, dd, *J* = 9.5, 8.9, SGlu H-2), 3.15 (1H, dd, *J* = 9.8, 8.9, SGlu H-4), 3.22 (1H, dd, *J* = 11.6, 9.8, OXyl H-5b), 3.26 (1H, ddd, *J* = 9.8, 5.6, 2.4, SGlu H-5), 3.34 (1H, dd, *J* = 8.9, 8.9, SGlu H-3), 3.42 (1H, dd, *J* = 8.7, 8.7, Oxyl H-3), 3.52 (1H, dd, *J* = 8.7, 7.2, OXyl H-2), 3.58 (1H, ddd, *J* = 9.8, 8.7, 5.2, OXyl H-4), 3.61 (1H, dd, *J* = 12.0, 5.6, SGlu H-6a), 3.81 (1H, dd, *J* = 12.0, 2.4, SGluH-6b), 3.96 (1H, dd, *J* = 11.6, 5.2, OXyl H-5b), 4.36 (1H, d, *J* = 9.5, SGlu H-1), 4.88 (1H, d, *J* = 7.2, OXyl H-1), 6.96 (1H, ddd, *J* = 8.1, 7.0, 0.9, H-5), 7.09 (1H, ddd, *J* = 8.3, 7.0, 1.1, H-6), 7.22 (1H, d, *J* = 8.3, 0.9, H-7), 7.63 (1H, dd, *J* = 8.1, 1.1, H-4). ^13^C NMR (200 MHz, CD_3_OD) *δ*: 62.6 (SGlu C-6), 66.9 (SXyl C-5), 71.06 (Oxyl C-4), 71.08 (SGlu C-4), 73.6 (SGlu C-2), 75.0 (OXyl C-2), 77.2 (OXyl C-3), 79.1 (SGlu C-3), 82.0 (SGlu C-5), 88.9 (SGlu C-1), 107.0 (OXyl C-1), 112.2 (C-7), 112.9 (C-2), 119.2 (C-4), 120.2 (C-5), 121.7 (C-3a), 124.2 (C-6), 136.3 (C-7a), 141.9 (C-3). HRMS (ESI) *m*/*z*: [M + Na]^+^ Calcd for C_19_H_25_NNaO_10_S 482.1091; found 482.1085.

#### 3-(β-l-Arabinopyranosyloxy)-1*H*-indol-2-yl 1-thio-β-d-glucopyranoside (9e)

Following the method similar to that used for the preparation of 8b, 14e (168 mg, 0.21 mmol) was treated with K_2_CO_3_ in MeOH to give the title compound (9e, 73 mg, 0.16 mmol, 79%) as a pale yellow microcrystalline solid. Mp 144–145 °C. [*α*]^26^_D_ −7.6 (*c* = 1.0, MeOH). IR (KBr): 3397, 2922, 2881, 2444, 1456, 1341, 1240, 1068, 1022, 947, 912, 873 cm^−1^. ^1^H NMR (800 MHz, D_2_O) *δ*: 3.11 (1H, dd, *J* = 9.7, 9.0, SGlu H-2), 3.21 (1H, dd, *J* = 9.9, 9.0, SGlu H-4), 3.29 (1H, ddd, *J* = 9.9, 5.6, 2.3, SGlu H-5), 3.39 (1H, dd, *J* = 9.0, 9.0, SGlu H-3), 3.45 (1H, dd, *J* = 13.5, 2.3, SGlu H-6a), 3.60 (1H, dd, *J* = 12.3, 5.6, OArab H-5a), 3.64 (1H, dd, *J* = 9.4, 3.4, OArab H-3), 3.76 (1H, dd, *J* = 12.3, 2.3, OArab H-5b), 3.83 (1H, dd, *J* = 9.4, 7.4, OArab H-2), 3.85–3.87 (2H, m, SGlu H-6b and OArab H-4), 4.50 (1H, d, *J* = 9.7, SGlu H-1), 4.87 (1H, d, *J* = 7.4, OArab H-1), 7.05 (1H, ddd, *J* = 8.1, 8.1, 0.9, H-5), 7.17 (1H, ddd, *J* = 8.1, 8.1, 1.0, H-6), 7.31 (1H, dd, *J* = 8.1, 0.9, H-7), 7.60 (1H, dd, *J* = 8.1, 1.0, H-4). ^13^C NMR (200 MHz, D_2_O) *δ*: 60.8 (SGlu C-6), 66.2 (OArab C-5), 68.0 (OArab C-4), 69.3 (SGlu C-4), 71.1 (OArab C-2), 72.0 (SGlu C-2), 72.1 (OArab C-3), 77.0 (SGlu C-3), 80.1 (SGlu C-5), 87.1 (SGlu C-1), 104.9 (OArab C-1), 111.9 (C-7), 112.4 (C-2), 117.7 (C-4), 119.5 (C-3a), 120.1 (C-5), 124.0 (C-6), 134.6 (C-7a), 139.3 (C-3). HRMS (ESI) *m*/*z*: [M + Na]^+^ Calcd for C_19_H_25_NNaO_10_S 482.1091; found 482.1085.

#### 3-(β-d-Maltopyranosyloxy)-1*H*-indol-2-yl 1-thio-β-d-glucopyranoside (9f)

Following the method similar to that used for the preparation of 8b, 14f (100 mg, 82 μmol) was treated with K_2_CO_3_ in MeOH to give the title compound (9f, 35 mg, 52 mmol, 63%) as a pale red microcrystalline solid. Mp 169–170 °C. [*α*]^26^_D_ +8.7 (*c* = 1.0, MeOH). IR (KBr): 3345, 3277, 2868, 1683, 1340, 1240, 1146, 1103, 1071, 1045, 914, 841 cm^−1^. ^1^H NMR (800 MHz, D_2_O) *δ*: 3.25 (1H, dd, *J* = 9.7, 9.0, SGlu H-2), 3.35 (1H, dd, *J* = 9.8, 9.0, SGlu H-4), 3.42 (1H, ddd, *J* = 9.8, 5.6, 2.3, SGlu H-5), 3.44 (1H, dd, *J* = 10.1, 9.2, OMal H-4), 3.49 (1H, br m, OMal H-5), 3.51 (1H, dd, *J* = 9.1, 9.0, SGlu H-3), 3.61 (1H, dd, *J* = 9.7, 4.0, OMal H-2′), 3.68 (1H, dd, *J* = 9.6, 7.9, OMal H-2), 3.71 (1H, dd, *J* = 9.7, 9.7, OMal H-3′), 3.72–3.76 (2H, m, OMal H-5′ and SGlu H-6a), 3.77–3.83 (3H, m, OMal H-4′, OMal H-6a and H-6′a), 3.84–3.95 (4H, m, OMal H-3, H-6b, H-6′b, SGlu H-6b), 4.62 (1H, d, *J* = 9.7, SGlu H-1), 5.02 (1H, d, *J* = 7.9, OMal H-1), 5.44 (1H, d, *J* = 4.0, OMal H-1′), 7.17 (1H, ddd, *J* = 8.0, 7.2, 0.9, H-5), 7.29 (1H, ddd, *J* = 8.3, 7.2, 1.0, H-6), 7.43 (1H, dd, *J* = 8.3, 0.9, H-7), 7.75 (1H, dd, *J* = 8.0, 1.0, H-4). ^13^C NMR (200 MHz, D_2_O) *δ*: 60.4/60.6/60.8 (OMal C-6, C-6′ and SGlu C-6), 69.3 (SGlu C-4), 69.4 (OMal C-4), 71.8 (OMal C-2′), 72.0 (SGlu C-2), 72.8 (OMal C-5′), 72.9 (OMal C-3′), 73.3 (OMal C-2), 74.8 (OMal C-5), 76.0 (OMal C-3), 76.6 (OMal C-4′), 77.1 (SGlu C-3), 80.0 (SGlu C-5), 87.2 (SGlu C-1), 99.8 (OMal C-1′), 104.4 (OMal C-1), 111.9 (C-7), 112.7 (C-2), 117.9 (C-4), 119.5 (C-3a), 120.1 (C-5), 124.0 (C-6), 134.6 (C-7a), 139.4 (C-3). HRMS (ESI) *m*/*z*: [M + Na]^+^ Calcd for C_26_H_37_NNaO_16_S 674.1725; found 674.1724.

#### *N*-Acetyl-2-(ethylthio)-1*H*-indol-3-yl 2,3,4,6-tetra-*O*-propionyl-1-thio-β-d-glucopyranoside (15)

A mixture of 2 (300 mg, 1.29 mmol), ethanethiol (98 μL, 1.35 mmol), BF_3_·Et_2_O (0.49 mL, 3.86 mmol), and 1,1,1,3,3,3-hexafluoropropan-2-ol (HFIP, 1.5 mL) was stirred at rt for 2 h. After removal of HFIP *in vacuo*, the residue was treated with 5a (3.0 g, 6.43 mmol) in a mixture of saturated aqueous K_3_PO_4_ (30 mL) and CH_2_Cl_2_ (10 mL) containing tetrabutylammonium hydrogen sulfate (TBAHS, 218 mg, 0.64 mmol) at rt for 30 min. The reaction mixture was poured into cold water (30 mL) and extracted with EtOAc (20 mL × 3). The extract was washed with brine (30 mL) and concentrated *in vacuo*. The residue was purified by column chromatography (*n*-hexane/EtOAc = 7/1) to give the title compound (15, 251 mg, 0.40 mmol, 31%) as a colourless viscous oil. [*α*]^26^_D_ −9.5 (*c* = 1.0, CH_3_Cl). IR (neat): 1738, 1697, 1360, 1296, 1163, 1064, 1016, 887, 806, 752 cm^−1^. ^1^H NMR (800 MHz, CDCl_3_) *δ*: 1.09 [9H, t, *J* = 7.8, C(O)CH_2_C*H*_3_], 1.13 [3H, t, *J* = 7.8, C(O)CH_2_C*H*_3_], 1.16 (each 3H, t, *J* = 7.8, SCH_2_C*H*_3_), 2.26–2.44 [8H, m, C(O)C*H*_2_CH_3_], 2.76–2.85 (2H, m, SC*H*_2_CH_3_), 2.89 [3H, s, C(O)C*H*_3_], 3.75–3.77 (1H, m, Glu H-5), 4.16 (1H, d, *J* = 12.0, Glu H-6a), 4.26 (1H, dd, *J* = 12.0, 5.2, H-6b), 5.16 (1H, d, *J* = 7.8, Glu H-1), 5.22 (1H, dd, *J* = 9.6, 9.6 H-4), 5.31 (1H, dd, *J* = 9.6, 9.6 H-3), 5.42 (1H, dd, *J* = 9.6, 7.8, H-2), 7.27 (1H, dd, *J* = 7.8, 7.8, H-5), 7.39 (1H, dd, *J* = 7.8, 7.8, H-6), 7.72 (1H, d, *J* = 7.8, H-4), 8.35 (1H, d, *J* = 7.8, H-7). ^13^C NMR (200 MHz, CDCl_3_) *δ*: 8.89/8.95/8.98/9.1 [C(O)CH_2_*C*H_3_], 14.5 (SCH_2_*C*H_3_), 27.26/27.34/27.4/27.6 [C(O)*C*H_2_CH_3_], 28.2 [C(O)*C*H_3_], 31.4 (S*C*H_2_CH_3_), 61.9 (Glu C-6), 68.0 (Glu C-4), 71.3 (Glu C-2), 72.3 (Glu C-5), 72.6 (Glu C-3), 102.2 (Glu C-1), 116.2 (C-7), 118.2 (C-4), 118.3 (C-2), 122.9 (C-3a), 123.5 (C-5), 126.7 (C-6), 135.7 (C-7a), 146.9 (C-3), 171.1/172.8/172.9/173.6/174.0 (CO). HRMS (ESI) *m*/*z*: [M + Na]^+^ Calcd for C_30_H_39_NNaO_11_S 644.2136; found 644.2133.

#### 2-(Ethylthio)-1*H*-indol-3-yl β-d-glucopyranoside (8g)

Following the method similar to that used for the preparation of 8b, 15 (101 mg, 0.16 mmol) was treated with K_2_CO_3_ in MeOH to give the title compound (8g, 53 mg, 0.15 mmol, 92%) as a pale green microcrystalline solid. Mp 95–96 °C. [*α*]^26^_D_ +167.8 (*c* = 1.0, MeOH). IR (KBr): 3395, 1622, 1533, 1449, 1341, 1238, 1071, 1011, 897 cm^−1^. ^1^H NMR (800 MHz, CD_3_OD) *δ*: 1.24 (3H, t, *J* = 7.4, SCH_2_C*H*_3_), 2.86/2.94 (each 1H, dq, *J* = 14.7, 7.4, SC*H*_2_CH_3_), 3.30–3.32 (1H, m, OGlu H-5), 3.46 (2H, br m, OGlu H-2 and H-4), 3.53 (1H, dd, *J* = 8.6, 8.6, OGlu H-3), 3.73 (1H, dd, *J* = 11.8, 5.1, OGlu H-6a), 3.84 (1H, dd, *J* = 11.8, 2.5, H-6b), 4.91 (1H, d, *J* = 7.8, OGlu H-1), 6.99 (1H, ddd, *J* = 8.0, 7.0, 1.0, H-5), 7.10 (1H, ddd, *J* = 8.2, 7.0, 1.2, H-6), 7.24 (1H, d, *J* = 8.2, H-7), 7.75 (1H, d, *J* = 8.0, H-4). ^13^C NMR (200 MHz, CD_3_OD) *δ*: 15.5 (SCH_2_*C*H_3_), 31.0 (S*C*H_2_CH_3_), 62.6 (OGlu C-6), 71.4 (OGlu C-4), 75.4 (OGlu C-2), 77.9 (OGlu C-3), 78.1 (OGlu C-5), 106.4 (OGlu C-1), 112.0 (C-7), 117.3 (C-2), 119.1 (C-4), 120.1 (C-5), 122.2 (C-6), 123.6 (C-3a), 135.8 (C-7a), 140.5 (C-3). HRMS (ESI) *m*/*z*: [M + Na]^+^ Calcd for C_16_H_21_NNaO_16_S 378.0982; found 378.0977.

#### *N*-Acetyl-3-(2,3,4,6-tetra-*O*-acetyl-β-d-glucopyranosyloxy)-1*H*-indol-2-yl 2,3,4,6-tetra-*O*-acetyl-1-thio-β-d-glucopyranoside (10a)

A mixture of 1 (70 mg, 0.143 mmol), acetic anhydride (Ac_2_O, 0.16 mL, 1.72 mmol), DMAP (1.0 mg, 7 μmol), and pyridine (3.0 mL) was stirred at rt for 16 h. The reaction mixture was poured into cold water (10 mL) and extracted with EtOAc (7 mL × 3). The extract was successively washed with 10% H_2_SO_4_ (7 mL × 4) and brine (7 mL) and concentrated *in vacuo*. The residue was purified by column chromatography (*n*-hexane/EtOAc = 5/1) to give the title compound (10a, 91 mg, 0.104 mmol, 73%) as a white amorphous. Mp 84–85 °C. IR (KBr): 2986, 2943, 2887, 1755, 1748, 1713, 1464, 1445, 1371, 1294, 1225, 1163, 1084, 925, 887 cm^−1^. ^1^H NMR (800 MHz, CDCl_3_) *δ*: 1.96/1.98/1.99/2.04/2.05/2.06/2.14/2.18 [each 3H, s, OC(O)C*H*_3_], 2.87 [each 3H, s, NC(O)C*H*_3_], 3.53 (1H, ddd, *J* = 10.2, 5.6, 2.2, SGlu H-5), 3.75 (1H, ddd, *J* = 10.2, 5.6, 2.5, OGlu H-5), 3.97 (1H, dd, *J* = 12.3, 2.2, SGlu H-6a), 4.12 (1H, dd, *J* = 12.3, 5.6, SGlu H-6b), 4.25 (1H, dd, *J* = 12.4, 2.5, OGlu H-6a), 4.27 (1H, dd, *J* = 12.4, 5.6, OGlu H-6b), 4.70 (1H, d, *J* = 10.1, SGlu H-1), 5.00 (1H, dd, *J* = 10.2, 9.4, SGlu H-2), 5.02 (1H, dd, *J* = 10.2, 9.4, SGlu H-4), 5.14 (1H, dd, *J* = 10.1, 9.4, OGlu H-4), 5.18 (1H, dd, *J* = 9.4, 9.4, SGlu H-3), 5.26 (1H, d, *J* = 7.8, OGlu H-1), 5.28 (1H, dd, *J* = 9.4, 9.4, OGlu H-3), 5.32 (1H, dd, *J* = 9.4, 7.8, OGlu H-2), 7.28 (1H, ddd, *J* = 8.0, 7.1, 0.9, H-5), 7.28 (1H, ddd, *J* = 8.0, 7.1, 0.9, H-5), 7.41 (1H, ddd, *J* = 8.5, 7.1, 1.3, H-6), 7.71 (1H, dd, *J* = 8.0, 1.3, H-4), 8.34 (1H, dd, *J* = 8.5, 0.9, H-7). ^13^C NMR data were in good agreement with the literature values.^18^ HRMS (ESI) *m*/*z*: [M + Na]^+^ Calcd for C_38_H_46_NNaO_20_S 868.2318; found 868.2318.

#### 3-(2,3,4,6-Tetra-*O*-propionyl-β-d-glucopyranosyloxy)-1*H*--indol-2-yl 2,3,4,6-tetra-*O*-propionyl-1-thio-β-d-glucopyranoside (10b)

A mixture of 1 (70 mg, 0.143 mmol), propionic anhydride (0.22 mL, 1.72 mmol), DMAP (1.0 mg, 7 μmol), and pyridine (3.0 mL) was stirred at rt for 24 h. The reaction mixture was poured into cold water (10 mL) and extracted with EtOAc (7 mL × 3). The extract was successively washed with 10% H_2_SO_4_ (7 mL × 4) and brine (7 mL) and concentrated *in vacuo*. The residue was purified by column chromatography (*n*-hexane/EtOAc =10/1) to give the title compound (10b, 77 mg, 82 μmol, 57%) as a pale yellow amorphous. Mp 81–82 °C. [*α*]^20^_D_ −6.4 (*c* = 1.0, CHCl_3_). IR (KBr): 2982, 2943, 1751, 1352, 1275, 1179, 1084, 1067 cm^−1^. ^1^H NMR (800 MHz, CDCl_3_) *δ*: 1.04/1.07/1.08/1.09/1.12/1.13/1.15/1.18 [each 3H, t, *J* = 7.6, C(O)CH_2_C*H*_3_], 2.20–2.43 [16H, m, C(O)C*H*_2_CH_3_], 3.71 (1H, ddd, *J* = 9.8, 4.4, 2.0, SGlu H-5), 3.74 (1H, ddd, *J* = 9.7, 5.5, 2.3, OGlu H-5), 4.18 (1H, dd, *J* = 12.3, 2.0, OGlu H-6a), 4.24 (1H, dd, *J* = 12.4, 4.4, SGlu H-6a), 4.27 (1H, dd, *J* = 12.3, 5.5, OGlu H-6b), 4.33 (1H, dd, *J* = 12.4, 2.0, SGlu H-6b), 4.56 (1H, d, *J* = 9.8, SGlu H-1), 4.98 (1H, dd, *J* = 9.8, 9.8, SGlu H-2), 5.01 (1H, dd, *J* = 9.8, 9.8, SGlu H-4), 5.03 (1H, d, *J* = 8.0, OGlu H-1), 5.18 (1H, dd, *J* = 9.7, 9.7, OGlu H-4), 5.22 (1H, dd, *J* = 9.8, 9.8, SGlu H-3), 5.26 (1H, dd, *J* = 9.7, 9.7, OGlu H-3), 5.31 (1H, dd, *J* = 9.7, 8.0, OGlu H-2), 7.07 (1H, ddd, *J* = 8.0, 6.9, 0.9, H-5), 7.22 (1H, ddd, *J* = 8.2, 6.9, 1.2, H-6), 7.34 (1H, br d-like, *J* = *ca.* 8.2, H-7), 7.67 (1H, dd, *J* = 8.0, 0.9, H-4), 8.72 (1H, br s, NH). ^13^C NMR (200 MHz, CDCl_3_) *δ*: 8.89/8.96(3C)/8.03/9.0/9.1 [C(O)CH_2_*C*H_3_], 27.30/27.31/27.34(2C)/27.42/27.45/27.5/27.6 [C(O)*C*H_2_CH_3_], 61.4 (SGlu C-6), 62.1 (OGlu C-6), 67.6 (SGlu C-4), 68.3 (OGlu C-4), 69.9 (SGlu C-2), 71.3 (OGlu C-2), 72.1 (OGlu C-5), 72.7 (OGlu C-3), 73.7 (SGlu C-3), 76.5 (SGlu C-5), 74.0 (SGlu C-1), 102.2 (OGlu C-1), 110.8 (C-2), 111.2 (C-7), 118.6 (C-4), 119.8 (C-5), 120.5 (C-3a), 123.7 (C-6), 135.0 (C-7a), 140.6 (C-3), 172.7/172.8/172.9/173.3/173.4/173.6/174.1/174.6 [*C*(O)CH_2_CH_3_]. HRMS (ESI) *m*/*z*: [M+Na]^+^ Calcd for C_44_H_60_NNaO_19_S 938.3475; found 938.3475.

#### 2-Acetyloxy-2,3-dihydro-1-benzofuran-3-one (16a)

A mixture of 17^22^ (600 mg, 3.41 mmol), *m*CPBA (70%, 1.26 mg, 5.11 mmol), and CH_2_Cl_2_ (15 mL) was stirred at rt for 3 h. The reaction was quenched by addition of a mixture of saturated aqueous NaHCO_3_ solution containing 10% NaHSO_3_ (15 mL), and the resulting mixture was extracted with CH_2_Cl_2_ (10 mL × 3). The extract was washed with brine (20 mL) and concentrated *in vacuo*. The residue was purified by column chromatography (*n*-hexane/EtOAc = 15/1) to give the title compound (16a,^25^ 417 mg, 2.17 mmol, 64%) as a colourless oil. ^1^H NMR (800 MHz, CDCl_3_) *δ*: 2.20 [3H, s, C(O)C*H*_3_], 6.21 (1H, s), 7.11 (1H, d, *J* = 8.3, H-7), 7.14 (1H, dd, *J* = 7.5, 7.5, H-5), 7.67 (1H, dd, *J* = 8.3, 7.5, H-6), 7.69 (1H, d, *J* = 7.5, H-4). ^13^C NMR (200 MHz, CDCl_3_) *δ*: 20.5 [C(O)*C*H_3_], 91.8 (C-2), 113.4 (C-7), 119.2 (C-3a), 123.1 (C-5), 124.8 (C-4), 139.0 (C-6), 169 [*C*(O)CH_3_], 171.4 (C-7a), 194.9 (C-3). HRMS (ESI) *m*/*z*: [M + Na]^+^ Calcd for C_10_H_8_NaO_4_ 215.0315; found 215.0307.

#### 2,3-Dihydro-1-benzofuran-3-one (18)

A mixture of 17 (1.77 g, 10.1 mmol), Na_2_SO_3_ (1.89 g, 15.1 mmol), H_2_O (25 mL), and MeOH (50 mL) was stirred at rt for 3 h. After removal of MeOH *in vacuo*, the aqueous residue was extracted with EtOAc (30 mL × 3). The extract was washed with brine (30 mL) and concentrated *in vacuo*. The residue was recrystallized from a mixture of *n*-hexane and EtOAc to give the title compound (18, 789 mg, 5.88 mmol, 59%) as a pale yellow microcrystalline solid. Mp 98–99 (lit.^26^ 100–102 °C, lit.^27^ 101–103 °C). ^1^H NMR (800 MHz, CDCl_3_) *δ*: 4.61 (2H, s, H-2), 7.08 (1H, dd, *J* = 7.7, 7.7, H-5), 7.13 (1H, d, *J* = 8.4, H-7), 7.60 (1H, dd, *J* = 8.4, 7.7, H-6), 7.66 (1H, d, *J* = 7.7, H-4). ^13^C NMR (200 MHz, CDCl_3_) *δ*: 74.6 (C-2), 113.6 (C-7), 121.1 (C-3a), 121.9 (C-5), 124.0 (C-4), 137.8 (C-6), 173.9 (C-7a), 199.8 (C-3). HRMS (ESI) *m*/*z*: [M + H]^+^ Calcd for C_8_H_7_O_2_ 9135.0441; found 135.0439.

#### 2-Bromo-2,3-dihydro-1-benzofuran-3-one (16b)

A mixture of 18 (771 mg, 5.75 mmol), NBS (1.13 g, 6.33 mmol), PTSA (109 mg, 0.58 mmol), and MeCN (30.0 mL) was stirred at rt for 6 h. The reaction mixture was poured into cold water (30 mL) and extracted with EtOAc (20 mL × 3). The extract was washed with brine (30 mL) and concentrated *in vacuo*. The residue was purified by column chromatography (*n*-hexane/EtOAc = 7/1) to give the title compound (16b,^28^ 1.14 g, 5.36 mmol, 93%) as a pale yellow microcrystalline solid. Mp 74–75 °C. IR (KBr): 1735, 1614, 1463, 1325, 1182, 1011, 762 cm^−1^. ^1^H NMR (800 MHz, CDCl_3_) *δ*: 6.50 (1H, s, H-2), 7.18 (1H, d, *J* = 8.4, H-7), 7.23 (1H,dd *J* = 7.4, 7.4, H-5), 7.70 (1H, ddd, *J* = 8.4, 7.4, 1.5, H-6), 7.77 (1H, br d-like, *J* = *ca.* 7.4, 1.5, H-4). ^13^C NMR (200 MHz, CDCl_3_) *δ*: 75.7 (C-2), 113.9 (C-7), 117.9 (C-3a), 124.0 (C-5), 125.5 (C-4), 138.8 (C-6), 170.2 (C-7a), 194.5 (C-3). HRMS (ESI) *m*/*z*: [M + H]^+^ Calcd for BrC_8_H_6_O_2_ 212.9546; found 212.9541.

### 3-(2,3,4,6-Tetra-*O*-propionyl-β-d-glucopyranosyloxy)benzofuran-2-yl 2,3,4,6-tetra-*O*-acetyl-1-thio-β-d-glucopyranoside (20)

A mixture of 16b (300 mg, 1.41 mmol), 3a (550 mg, 1.41 mmol), NaHCO_3_ (237 mg, 2.82 mmol), and CH_3_CN (20 mL) was stirred at rt for 1 h. After removal of CH_3_CN *in vacuo*, the residue, consisting primarily of 19, was treated with 5a (3.2 g, 7.04 mmol) in a mixture of saturated aqueous K_3_PO_4_ (43 mL) and CH_2_Cl_2_ (14 mL) containing TBAHS (239 mg, 0.70 mmol) at rt for 30 min. The reaction mixture was poured into cold water (100 mL) and extracted with EtOAc (100 mL × 4). The extract was washed with brine (100 mL) and concentrated *in vacuo*. The residue was purified by column chromatography (*n*-hexane/EtOAc = 7/1) to give the title compound (20, 649 mg, 0.73 mmol, 53%) as pale red microsrystaline solid. Mp 86–87 °C. [*α*]^22^_D_ −18.3 (*c* = 1.0, CHCl_3_). IR (KBr): 2984, 2945, 2882, 1763, 1379, 1364, 1240, 1169, 1086, 1059 cm^−1^. ^1^H NMR (800 MHz, CDCl_3_) *δ*: 1.08/1.09/1.11/1.15 [each 3H, t, *J* = 7.6, C(O)CH_2_C*H*_3_], 1.98/2.011/2.013/2.15 [each 3H, s, OC(O)C*H*_3_], 2.24–2.44 [8H, m, C(O)C*H*_2_CH_3_], 3.69 (1H, ddd, *J* = 10.2, 5.3, 2.2, SGlu H-5), 3.87 (1H, ddd, *J* = 10.2, 5.3, 2.3, OGlu H-5), 4.16 (1H, dd, *J* = 12.5, 5.3, SGlu H-6a), 4.18 (1H, dd, *J* = 12.5, 2.2, OGlu H-6a), 4.22 (1H, dd, *J* = 12.5, 2.2, SGlu H-6b), 4.27 (1H, dd, *J* = 12.3, OGlu H-6b), 4.78 (1H, d, *J* = 10.0, SGlu H-1), 4.91 (1H, dd, *J* = 10.1, 9.6, SGlu H-2), 5.02 (1H, dd, *J* = 9.6, 9.6, SGlu H-4), 5.33 (1H, dd, *J* = 9.6, 9.6, SGlu H-3), 5.34 (1H, dd, *J* = 9.6, 9.6, OGlu H-4), 5.34 (1H, dd, *J* = 9.6, 9.6, OGlu H-3), 5.34 (1H, dd, *J* = 7.7, 9.6, OGlu H-2), 5.40 (1H, d, *J* = 7.7, OGlu H-1), 7.22 (1H, ddd, *J* = 8.0, 6.9, 1.2, H-6), 7.34 (1H, ddd, *J* = 8.0, 6.9, 1.3, H-5), 7.36 (1H, dd, *J* = 8.0, 1.2, H-4), 7.61 (1H, dd, *J* = 8.0, 1.3, H-7). ^13^C NMR (200 MHz, CDCl_3_) *δ*: 9.0/9.1/9.16/9.19 [C(O)CH_2_*C*H_3_], 20.61/20.63(2C)/20.8 [C(O)*C*H_3_], 27.38/27.42/27.51/27.59 [C(O)*C*H_2_CH_3_], 61.8 (SGlu C-6), 62.0 (OGlu C-6), 67.9 (SGlu C-4), 68.3 (OGlu C-4), 70.6 (SGlu C-2), 71.1 (OGlu C-2), 72.0 (OGlu C-5), 72.6 (OGlu C-3), 74.1 (SGlu C-3), 76.6 (SGlu C-5), 84.1 (SGlu C-1), 100.6 (OGlu C-1), 111.8 (C-4), 119.7 (C-7), 122.9 (C-3a), 123.1 (C-6), 126.6 (C-5), 130.0 (C-2), 145.7 (C-3), 154.4 (C-7a), 169.5(2C)/170.2/170.7 [*C*(O)CH_3_], 172.9/173.0/173.6/174.2(*C*O). HRMS (ESI) *m*/*z*: [M + Na]^+^ Calcd for C_20_H_50_NaO_20_S 905.2508; found 905.2509.

#### 3-(β-d-Glucopyranosyloxy)benzofuran-2-yl 1-thio-β-d-glucopyranoside (11)

A mixture of 20 (160 mg, 0.18 mmol), K_2_CO_3_ (8 mg, 0.06 mmol), and MeOH (6.0 mL) was stirred at rt for 4 h. After removal of MeOH *in vacuo*, the residue was purified by column chromatography (CHCl_3_/MeOH = 5/1) to give the title compound (11, 53 mg, 0.11 mmol, 61%) as a colourless microcrystalline solid. Mp 198–199 °C. [*α*]^23^_D_ −4.1 (*c* = 1.0, DMSO). IR (KBr): 3385, 2934, 2886, 1447, 1354, 1273, 1117, 1026 cm^−1^. ^1^H NMR (800 MHz, DMSO-*d*_6_) *δ*: 3.08–3.12 (2H, m, SGlu H-2 and H-4), 3.17 (1H, ddd, *J* = 9.7, 5.4, 2.4, SGlu H-5), 3.23–3.28 (2H, m, SGlu H-3 and OGlu H-4), 3.31–3.35 (3H, m, OGlu H-2, H-3, and H-5), 3.47 (1H, ddd, *J* = 11.8, 5.7, 5.7, SGlu H-6a), 3.57 (1H, ddd, *J* = 11.5, 5.7, 5.7, OGlu H-6a), 3.65 (1H, ddd, *J* = 11.8, 5.3, 2.4, SGlu H-6b), 3.73 (1H, ddd, *J* = 11.5, 5.1, 2.4, OGlu H-6b), 4.07/4.18/4.63/4.70/4.73/4.75/4.97/5.04 (each 1H, br s-like, O*H*), 4.59 (1H, d, *J* = 9.6, SGlu H-1), 5.20 (1H, d, *J* = 7.2, OGlu H-1), 7.24 (1H, ddd, *J* = 8.1, 7.2, 0.9, H-6), 7.36 (1H, ddd, *J* = 7.2, 7.2, 1.3, H-5), 7.47 (1H, dd, *J* = 7.2, 0.9, H-4), 7.78 (1H, dd, *J* = 8.1, 1.3, H-7). ^13^C NMR (200 MHz, DMSO-*d*_6_) *δ*: 60.7 (OGlu C-6), 60.8 (SGlu C-6), 69.6 (OGlu C-4), 69.7 (SGlu C-4), 73.1 (SGlu C-2), 73.4 (OGlu C-2), 76.2 (OGlu C-5), 76.6 (OGlu C-3), 77.6 (SGlu C-3), 80.7 (SGlu C-5), 87.1 (SGlu C-1), 102.4 (OGlu C-1), 111.0 (C-4), 119.3 (C-7), 122.2 (C-6), 122.3 (C-3a), 125.3 (C-5), 132.8 (C-3), 144.0 (C-2), 153.3 (C-7a). HRMS (ESI) *m*/*z*: [M + Na]^+^ Calcd for C_20_H_50_NaO_20_S 905.2508; found 905.2509.

### Bioassays

#### Cell culture

The HFDPCs were originally purchased from TaKaRa Bio Inc. (Shiga, Japan), and the cells were grown in Dulbecco's modified eagle medium (DMEM) supplemented with 10% fetal bovine serum (FBS), penicillin (100 units per mL), and streptomycin (100 μg mL^−1^) at 37 °C in 5% CO_2_/95% air. The cells were harvested by incubation in phosphate-buffered saline (PBS) containing 1 mM ethylenediaminetetraacetic acid (EDTA) and 0.25% trypsin for approximately 5 min at 37 °C and were used for the subsequent bioassays.

### Measurement of cell proliferation

The HFDPCs (1.0 × 10^4^ cells per mL) were seeded in 96-well plates in serum-free DMEM (100 μL/well) and cultured for 24 h. Afterward, 100 μL of the test compounds at various concentrations dissolved in serum-free DMEM were added to each well, followed by incubation for 4 d. After the incubation, 20 μL of Cell Counting Kit-8 (CCK-8, Dojindo Laboratories, Kumamoto, Japan) reagent was added to each well, followed by incubation for 1 h at 37 °C. The absorbance was recorded at 450 nm (absorbance at 650 nm as reference) on a microplate reader (MTP-900Lab; HITACHI, Japan). The results are expressed as mean percentages of the control ± standard error of the mean (S.E.M.).

Cell proliferation was calculated using the following equation:Cell proliferation (%) = *A*/*B* × 100where *A* is the absorbance at 450 nm (650 nm for the test sample) and *B* is the absorbance at 450 nm (650 nm for the control).

## Author contributions

RS, KT, HN and SM performed chemistry and analytical experiments. YM, FI and TM prformed the biological experiments and analysed data. GT supervised the study, obtained funding and wrote the paper. The manuscript was reviewed and approved by all authors.

## Conflicts of interest

There are no conflicts to declare.

## Supplementary Material

MD-OLF-D6MD00273K-s001

## Data Availability

All data associated with this study have been included in either the manuscript or in the supplementary file associated with the manuscript. Supplementary information (SI) is available. See DOI: https://doi.org/10.1039/d6md00273k.
