# Supplementary material for: Structure–activity relationship for calanthoside, a potential hair-growth stimulant with an indole 2-S-,3-O-bis-glucoside structure. Part 1: role of two glucoside moieties in promoting HFDPC proliferation
Source: RSC Med Chem. 2026 May 15;17(7):3331–41. doi: 10.1039/d6md00273k (PMC13280977; doi:10.1039/d6md00273k)

Supplementary Information (SI) for RSC Medicinal Chemistry.
This journal is © The Royal Society of Chemistry 2026

SUPPLEMENTARY INFORMATION
Experimental Details and Characterization Data

Structure–activity relationship for calanthoside, a potential hair-growth stimulant with an indole 2-*S*-,3-*O*-bis-glucoside structure. Part 1: Role of two glucoside moieties in promoting HFDPC proliferation

Riko Suzuki[a], Katsuki Takashima[b], Yoshiaki Manse[c], Haruto Nishikawa[b], Mina Ashidate[b], Shinsuke Marumoto[d], Fumihiro Ishikawa[b], Toshio Morikawa*[c], Genzoh Tanabe*[a,b,c]

[a]Graduate School of Pharmacy, [b]Faculty of Pharmacy, [c]Pharmaceutical Research and Technology Institute, [d]Joint Research Centre, Kindai University, 3-4-1 Kowakae, Higashi-osaka, Osaka 577-8502, Japan

Table of Contents

^{1}H- AND ^{13}C-NMR SPECTROSCOPOC DATA OF SYNTHETIC COMPOUNDS S1–S37

^{1}H-NMR (800 MHz, D_2O) of 3-(*β*-D-Glucopyranosyloxy)-1*H*-indol-2-yl 1-thio-*β*-D-glactopyranoside (8b).

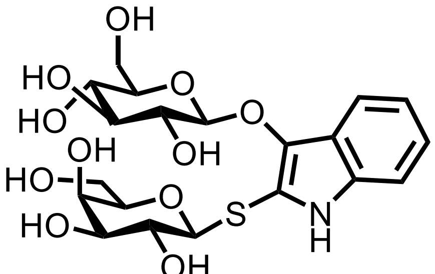

8b

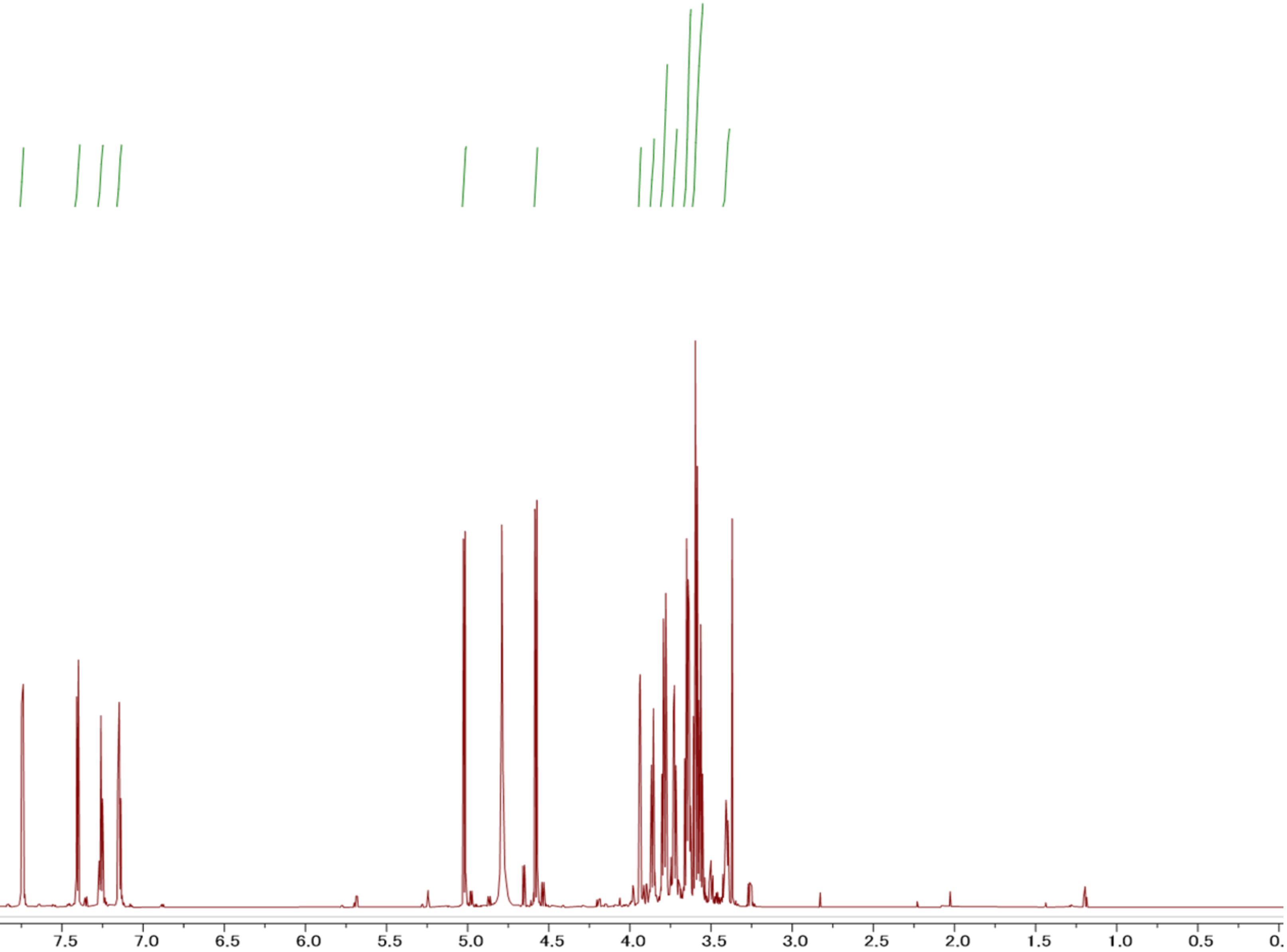

¹³C-NMR (200 MHz, D_2O) of 3-(*β*-D-Glucopyranosyloxy)-1*H*-indol-2-yl 1-thio-*β*-D-glactopyranoside (8b).

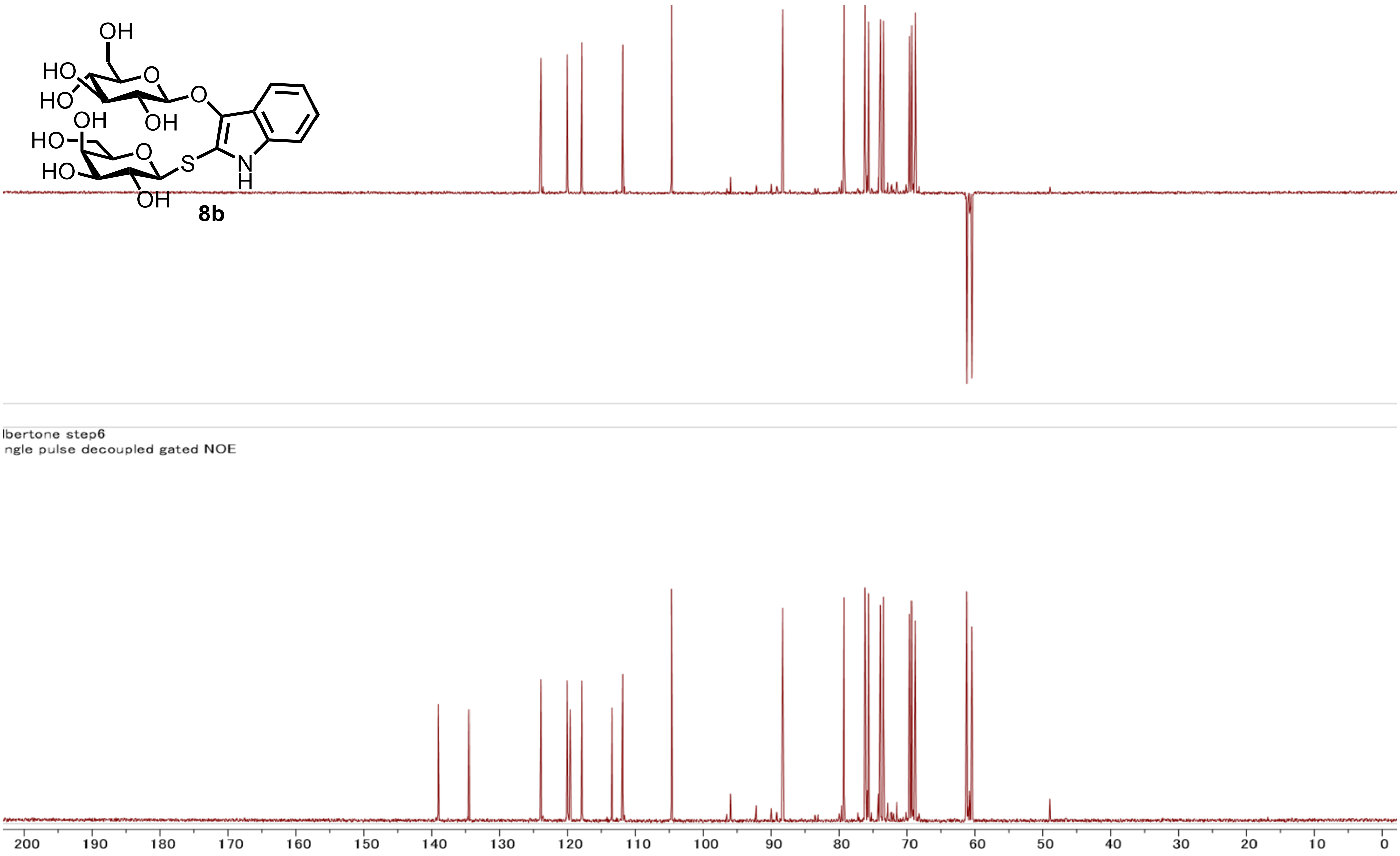

^{1}H-NMR (800 MHz, D_2O) of 3-(*β*-D-Glucopyranosyloxy) -1*H*-indol-2-yl 1-thio-*β*-D-mannopyranoside (8c).

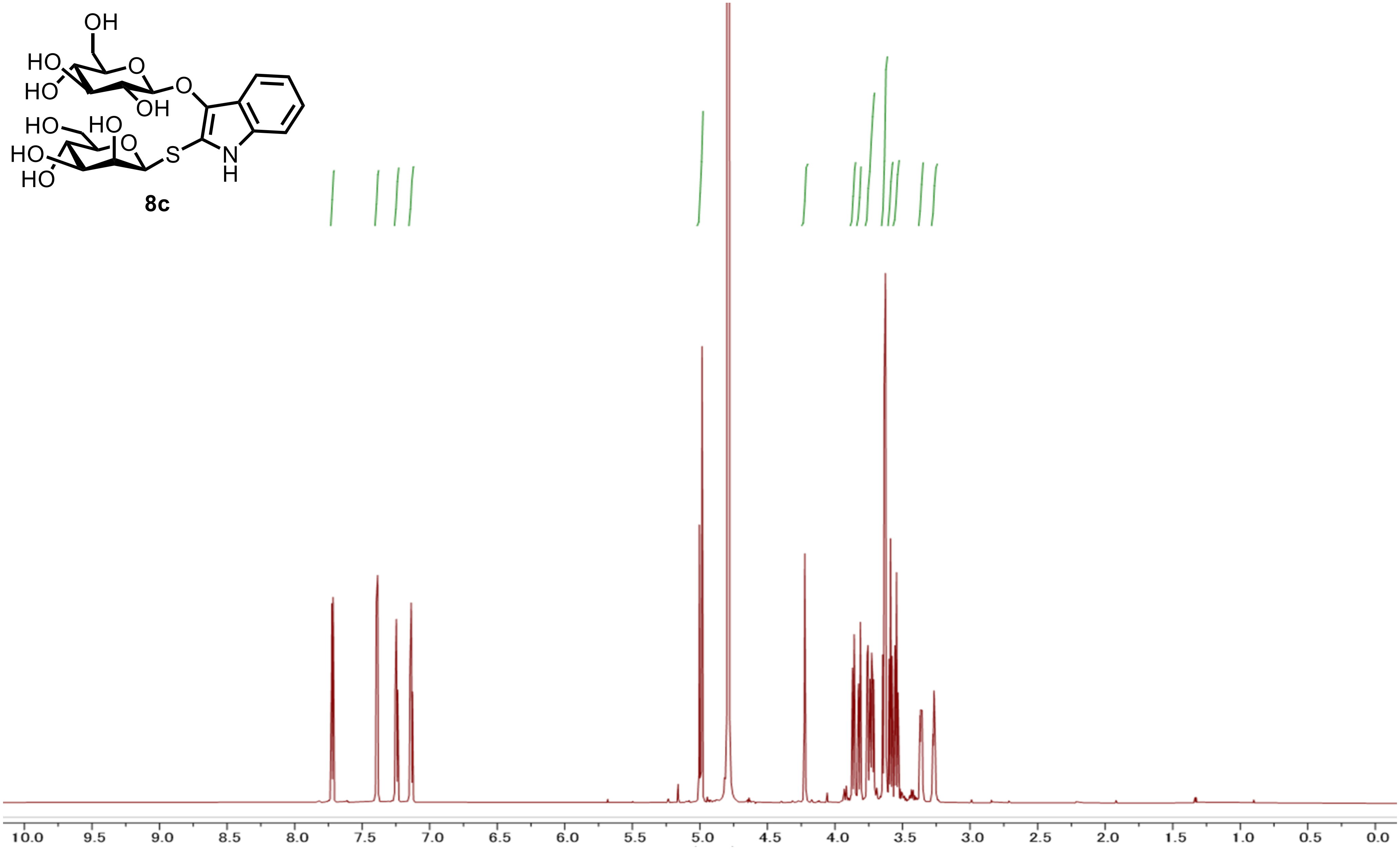

8c

^{13}C-NMR (200 MHz, D_2O) of 3-(*β*-D-Glucopyranosyloxy) -1*H*-indol-2-yl 1-thio-*β*-D-mannopyranoside (8c).

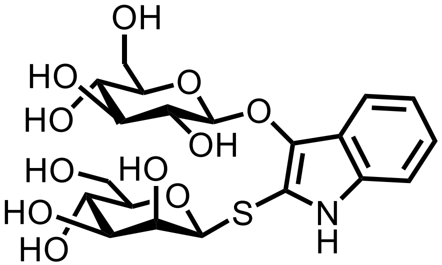

8c

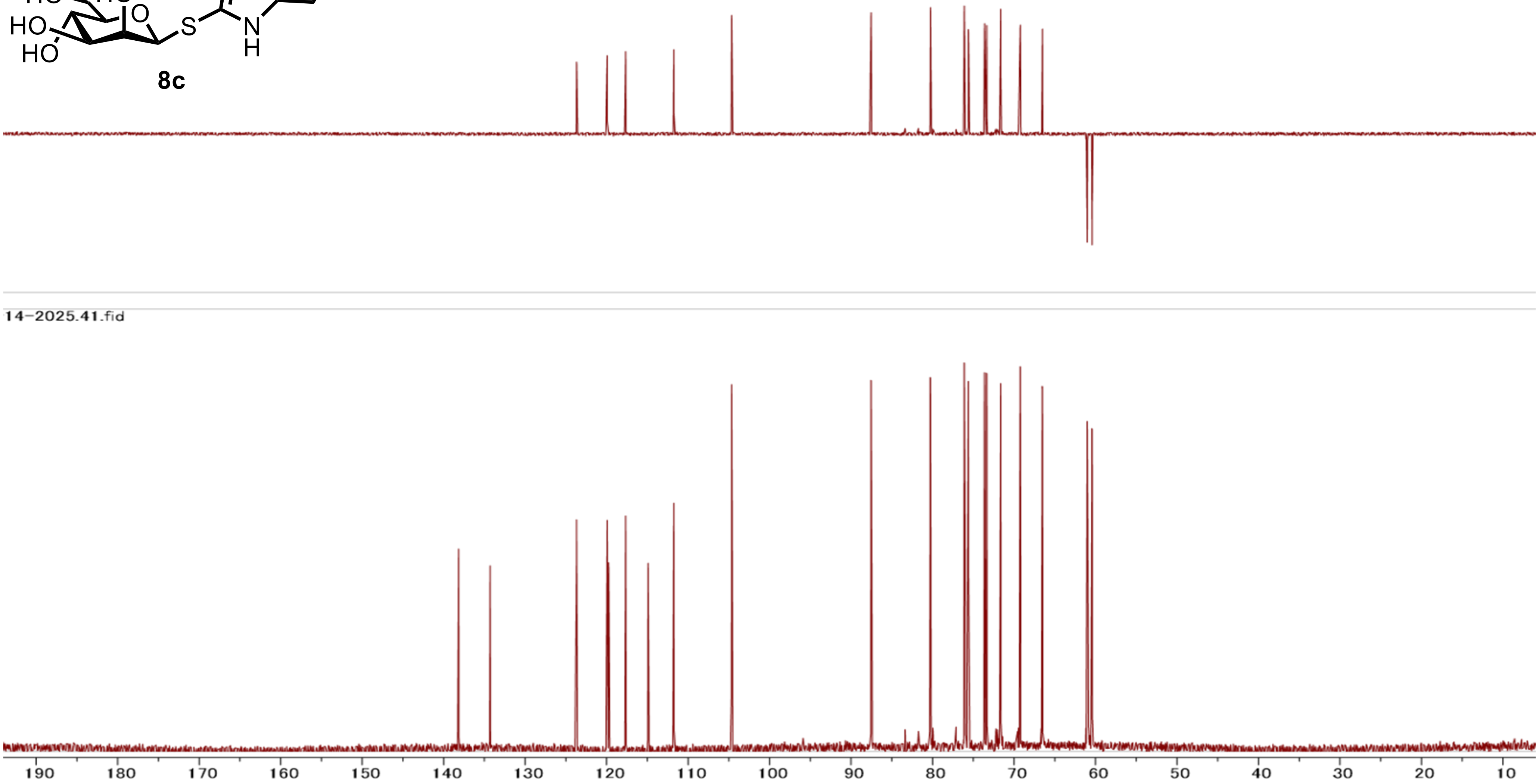

NOESY (800 MHz, D_2O) of 3-(*β*-D-Glucopyranosyloxy) -1*H*-indol-2-yl 1-thio-*β*-D-mannopyranoside (8c).

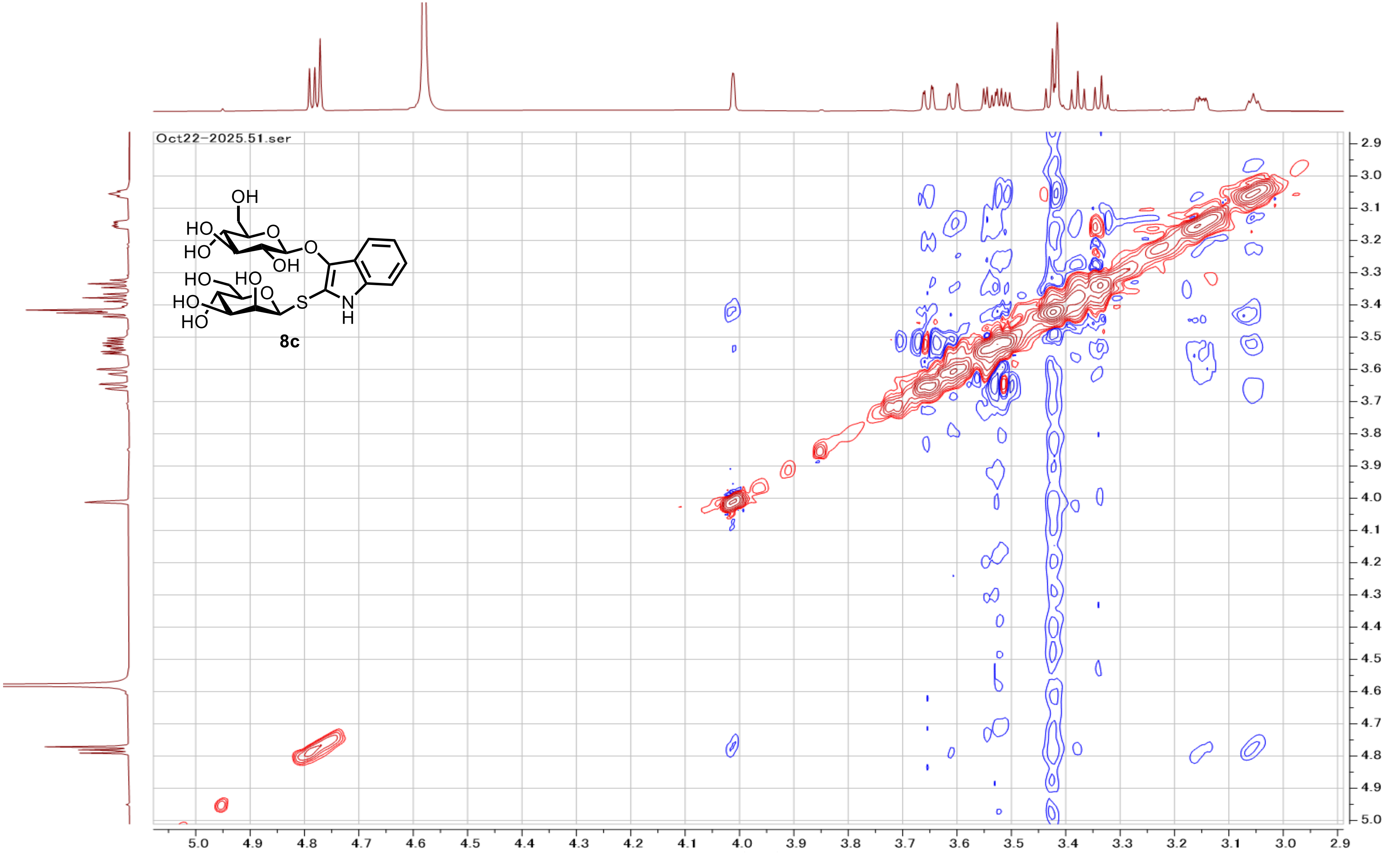

^{1}H-NMR (800 MHz, D_2O) of 3-(*β*-D-Glucopyranosyloxy)-1*H*-indol-2-yl 1-thio-*β*-D-xylopyranoside (8d).

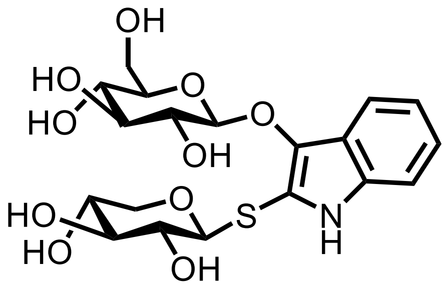

8d

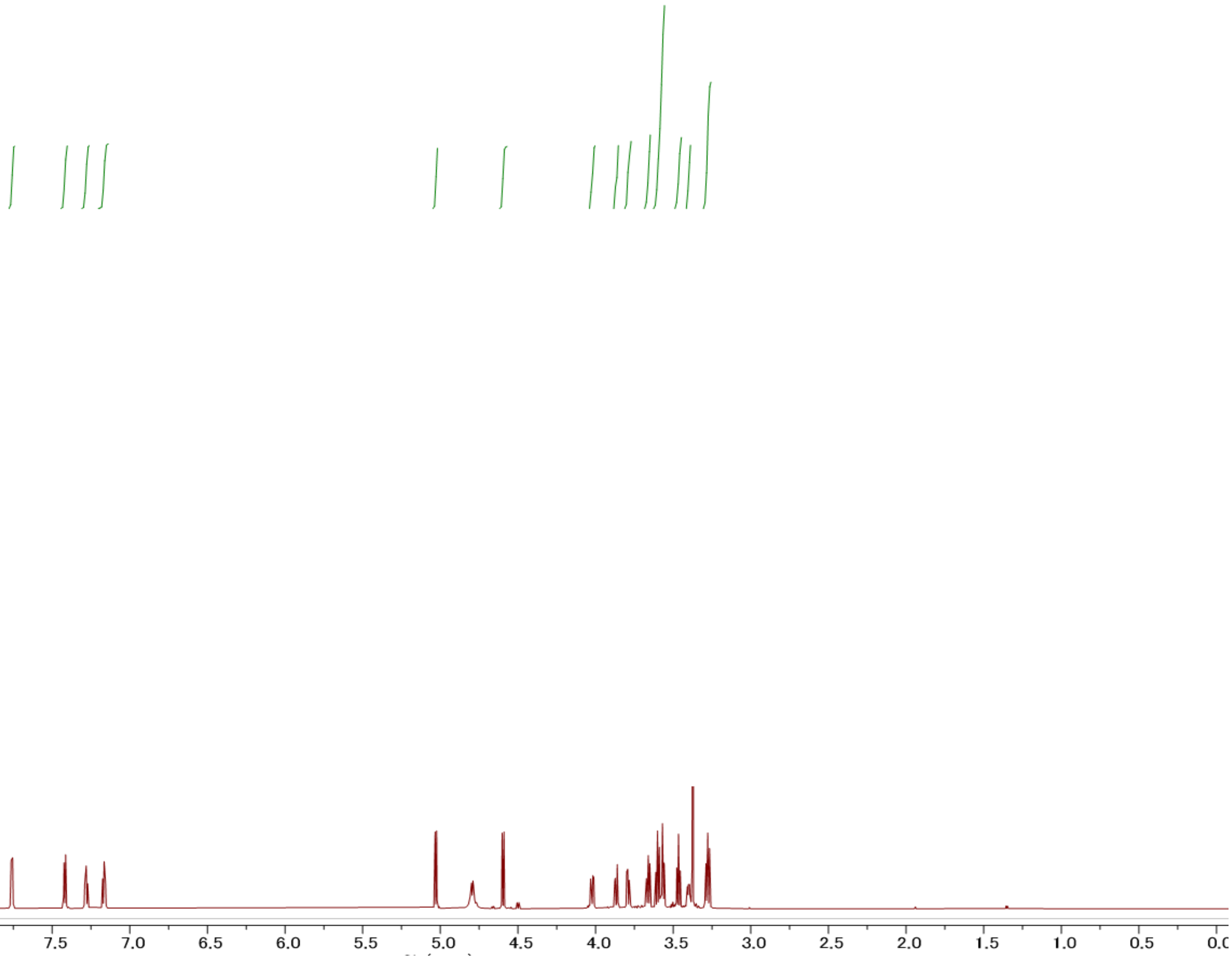

^{13}C-NMR (200 MHz, D_2O) of 3-(*β*-D-Glucopyranosyloxy)-1*H*-indol-2-yl 1-thio-*β*-D-xylopyranoside (8d).

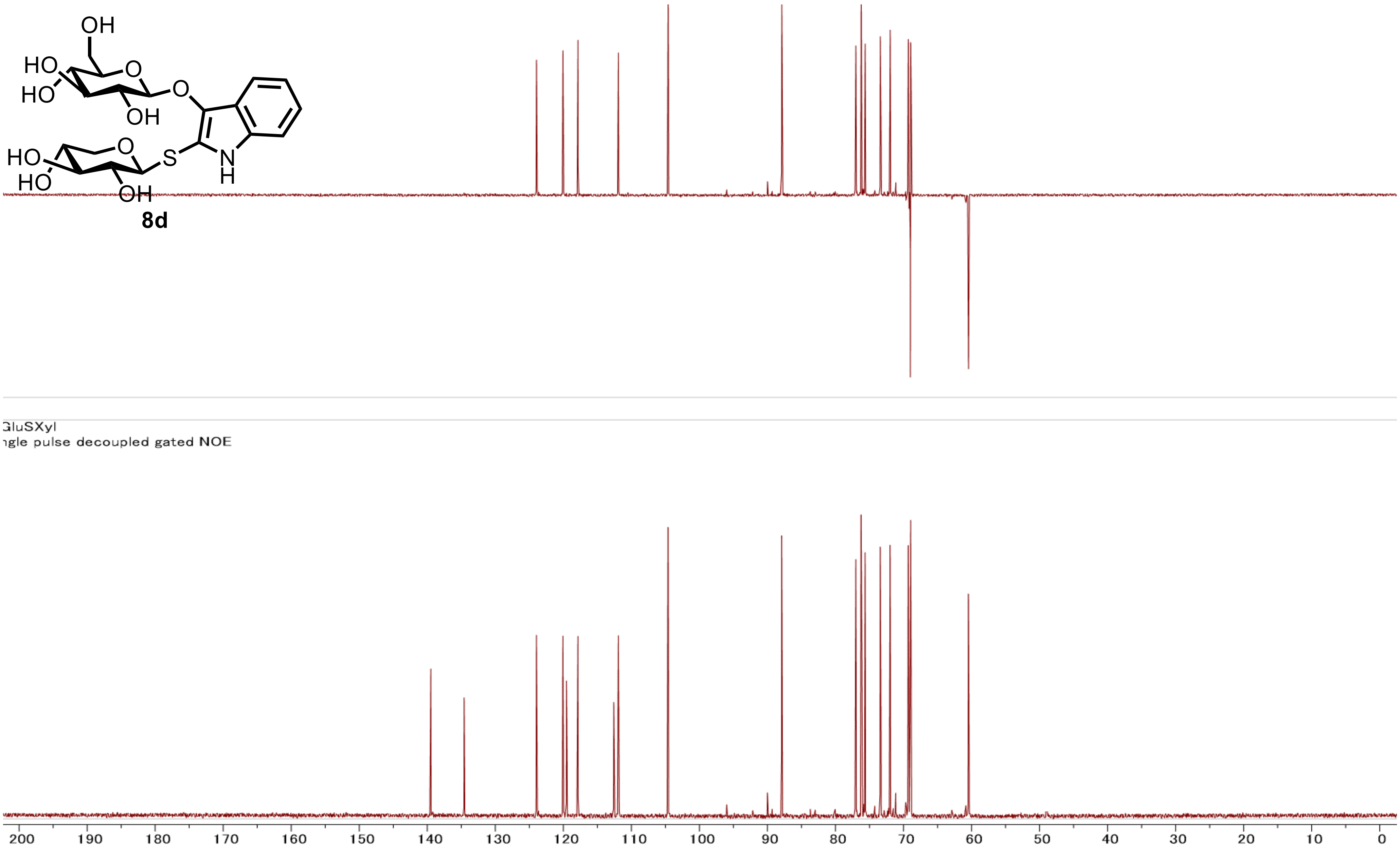

^{1}H-NMR (800 MHz, D_2O) of 3-(*β*-D-Glucopyranosyloxy)-1*H*-indol-2-yl 1-thio-*β*-L-arabinopyranoside (8e).

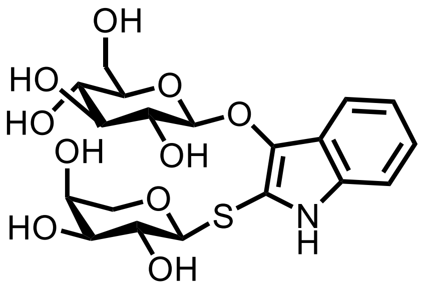

8e

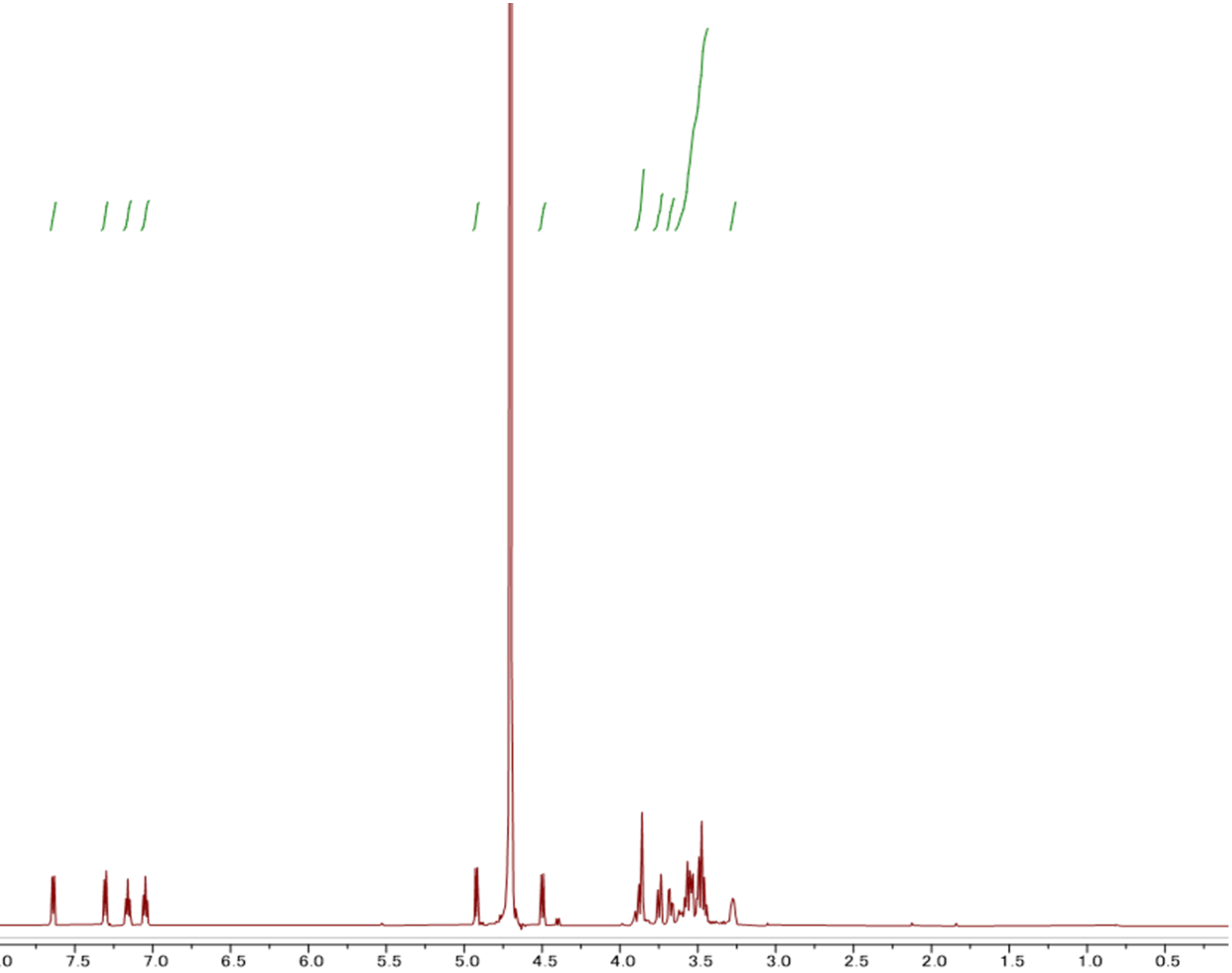

^{13}C-NMR (200 MHz, D_2O) of 3-(*β*-D-Glucopyranosyloxy)-1*H*-indol-2-yl 1-thio-*β*-L-arabinopyranoside (8e).

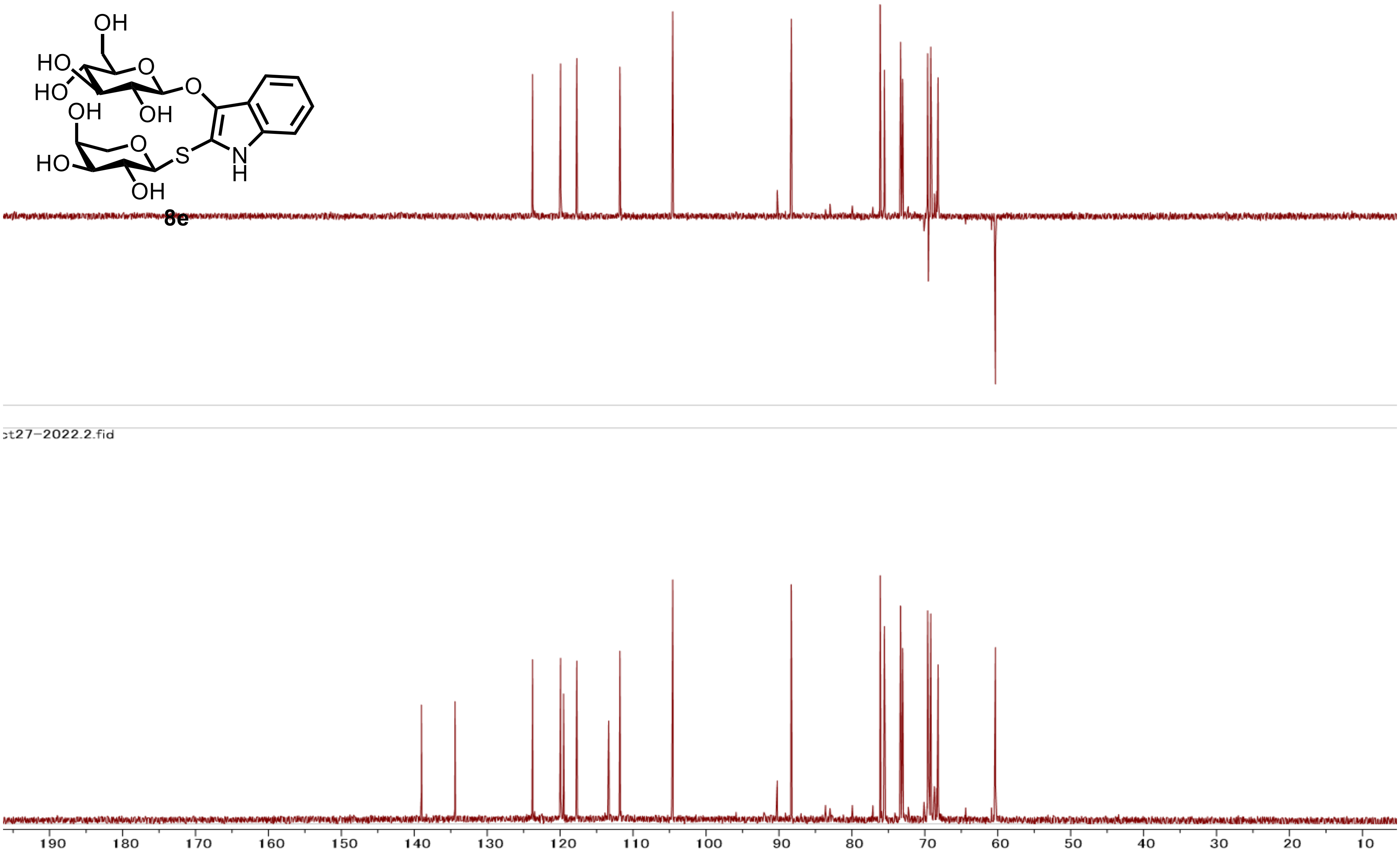

^{1}H-NMR (800 MHz, D_2O) of 3-(*β*-D-Glucopyranosyloxy)-1*H*-indol-2-yl 1-thio-*β*-D-maltopyranoside (8f).

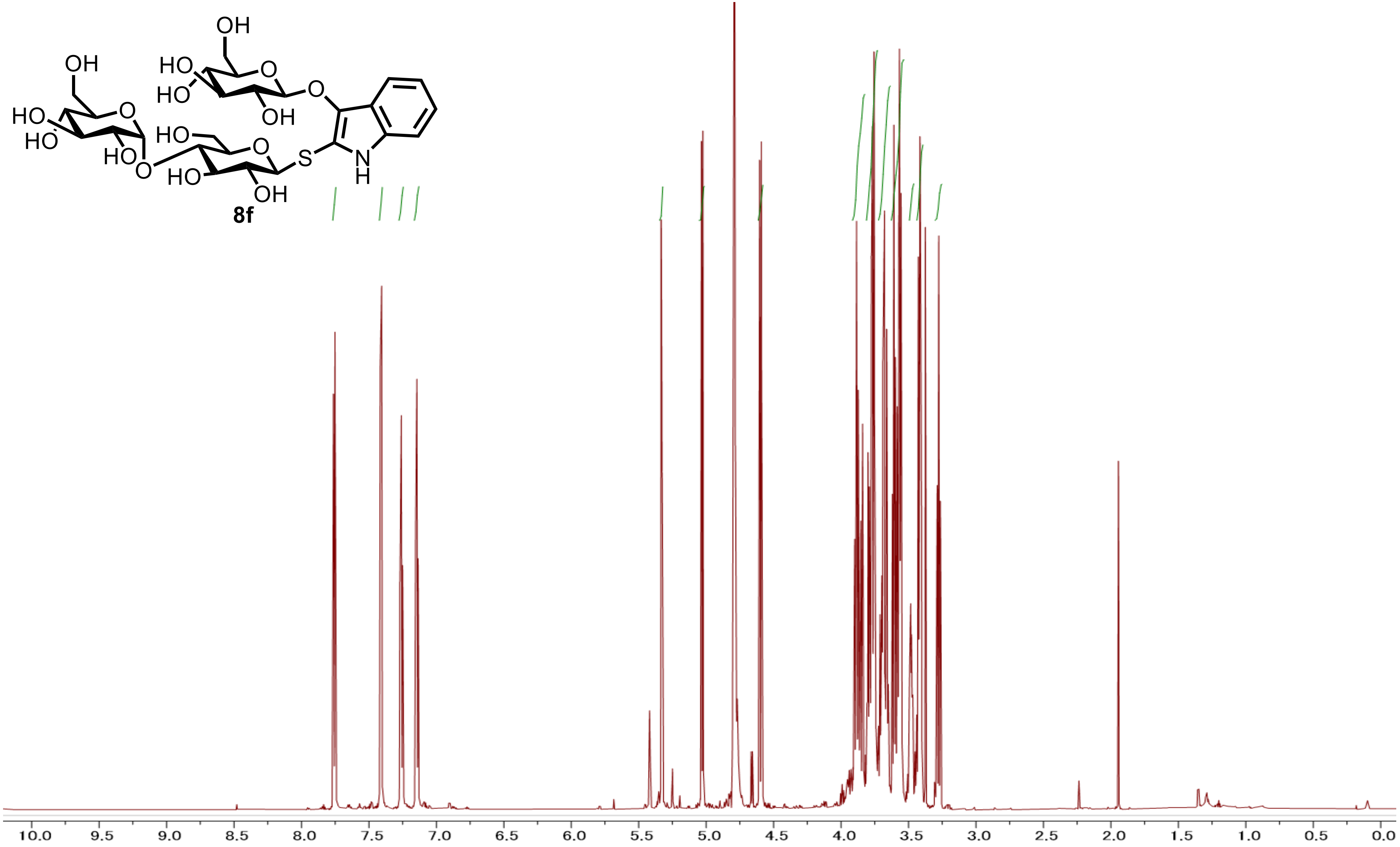

^{13}C-NMR (200 MHz, D_2O) of 3-(*β*-D-Glucopyranosyloxy)-1*H*-indol-2-yl 1-thio-*β*-D-maltopyranoside (8f).

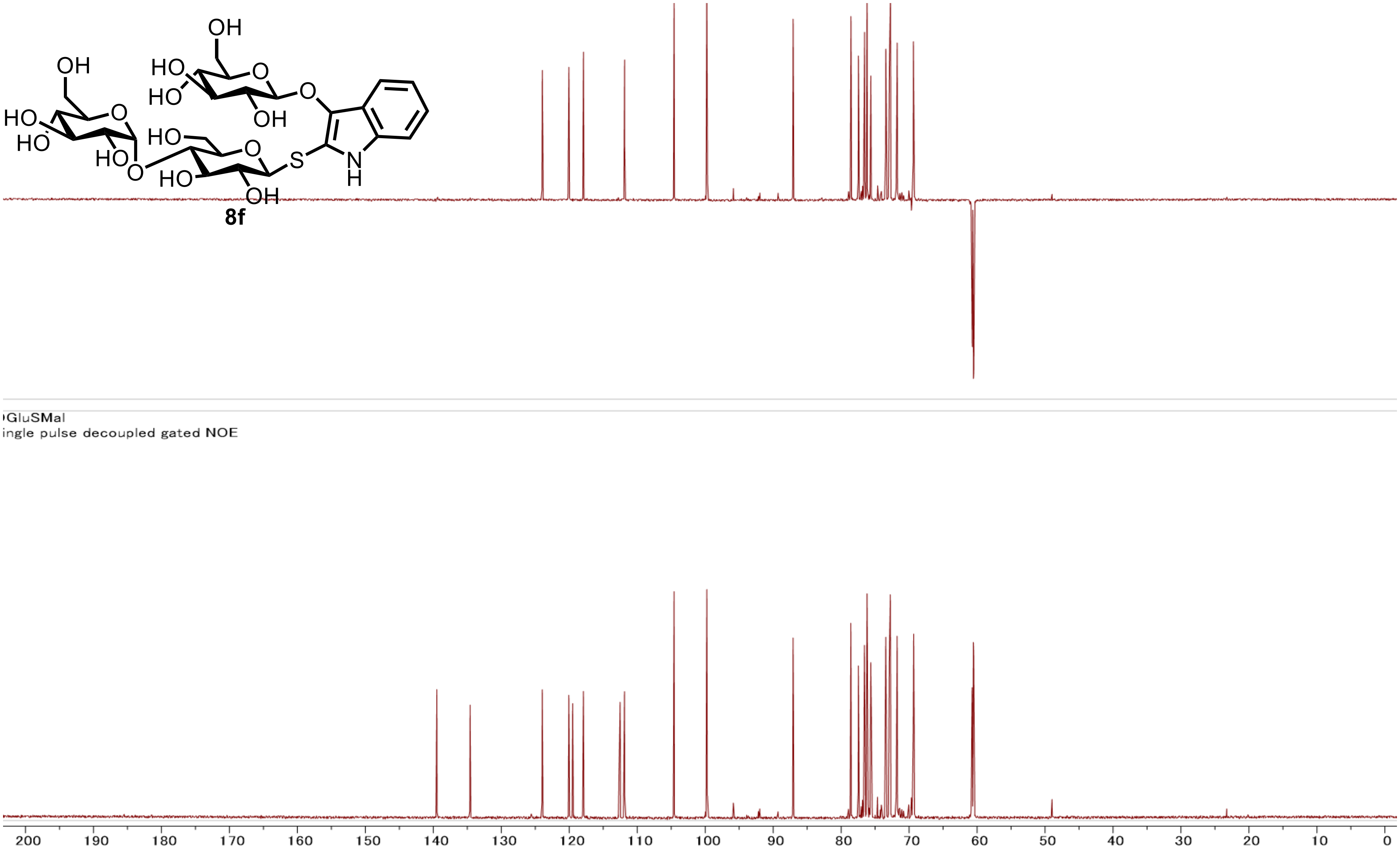

^{1}H-NMR (800 MHz, $CDCl_3$) of *N*-Acetyl-2-(ethylthio)-1*H*-indol-3-yl 2,3,4,6-tetra-*O*-propionyl-1-thio-*β*-D-glucopyranoside (15).

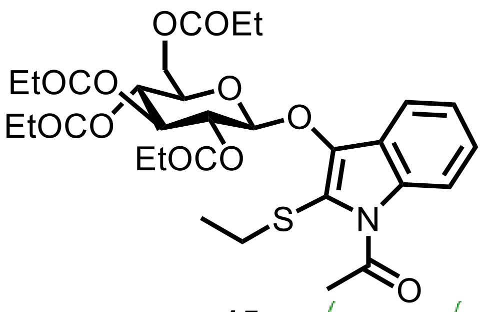

15

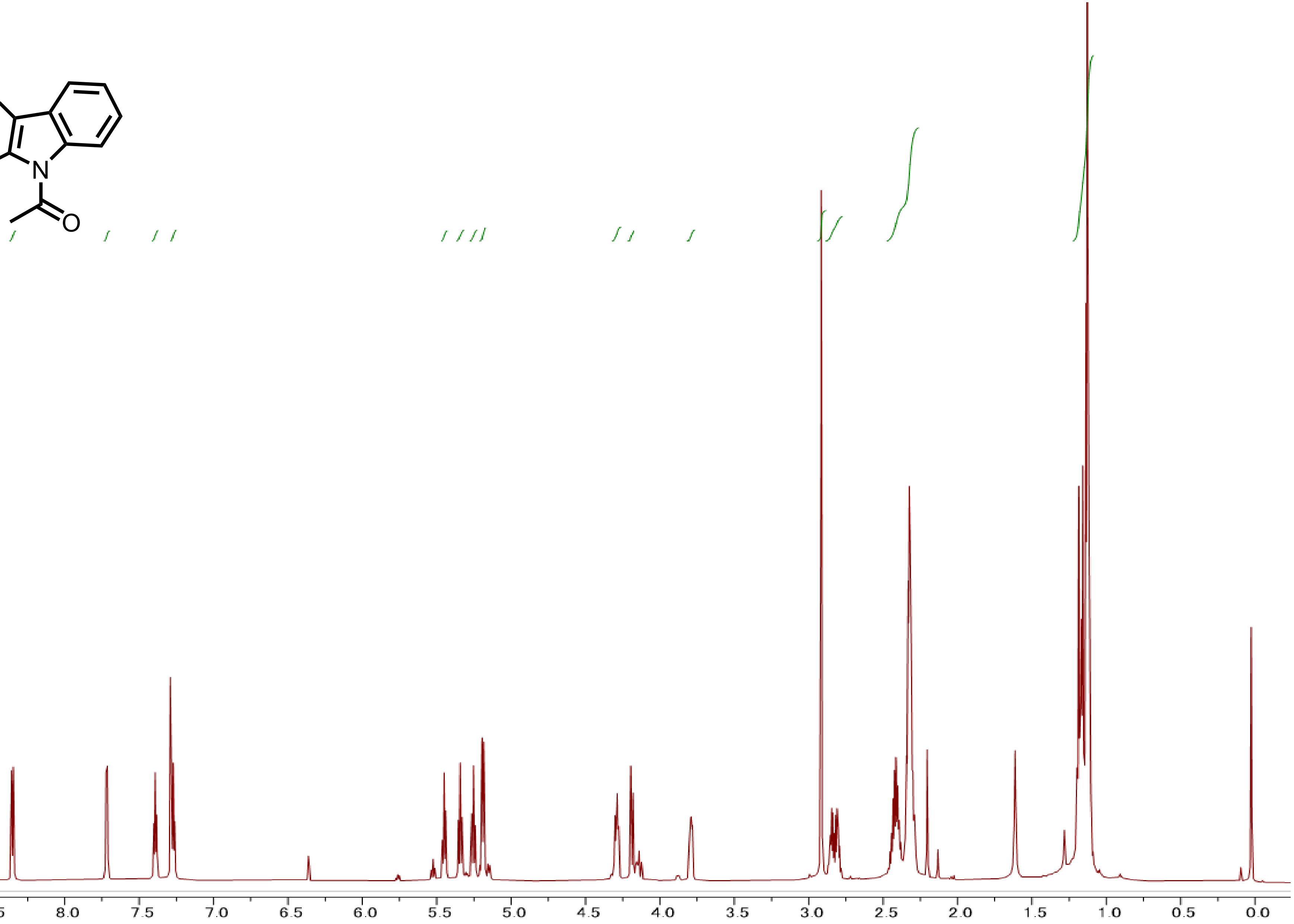

^{13}C-NMR (200 MHz, $CDCl_3$) of *N*-Acetyl-2-(ethylthio)-1*H*-indol-3-yl 2,3,4,6-tetra-*O*-propionyl-1-thio-*β*-D-glucopyranoside (15).

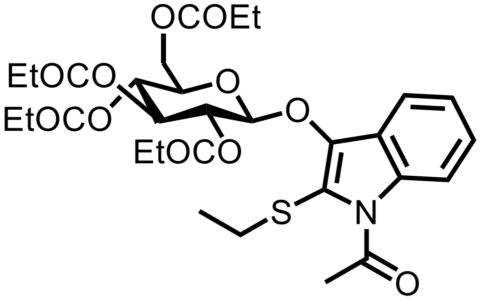

15

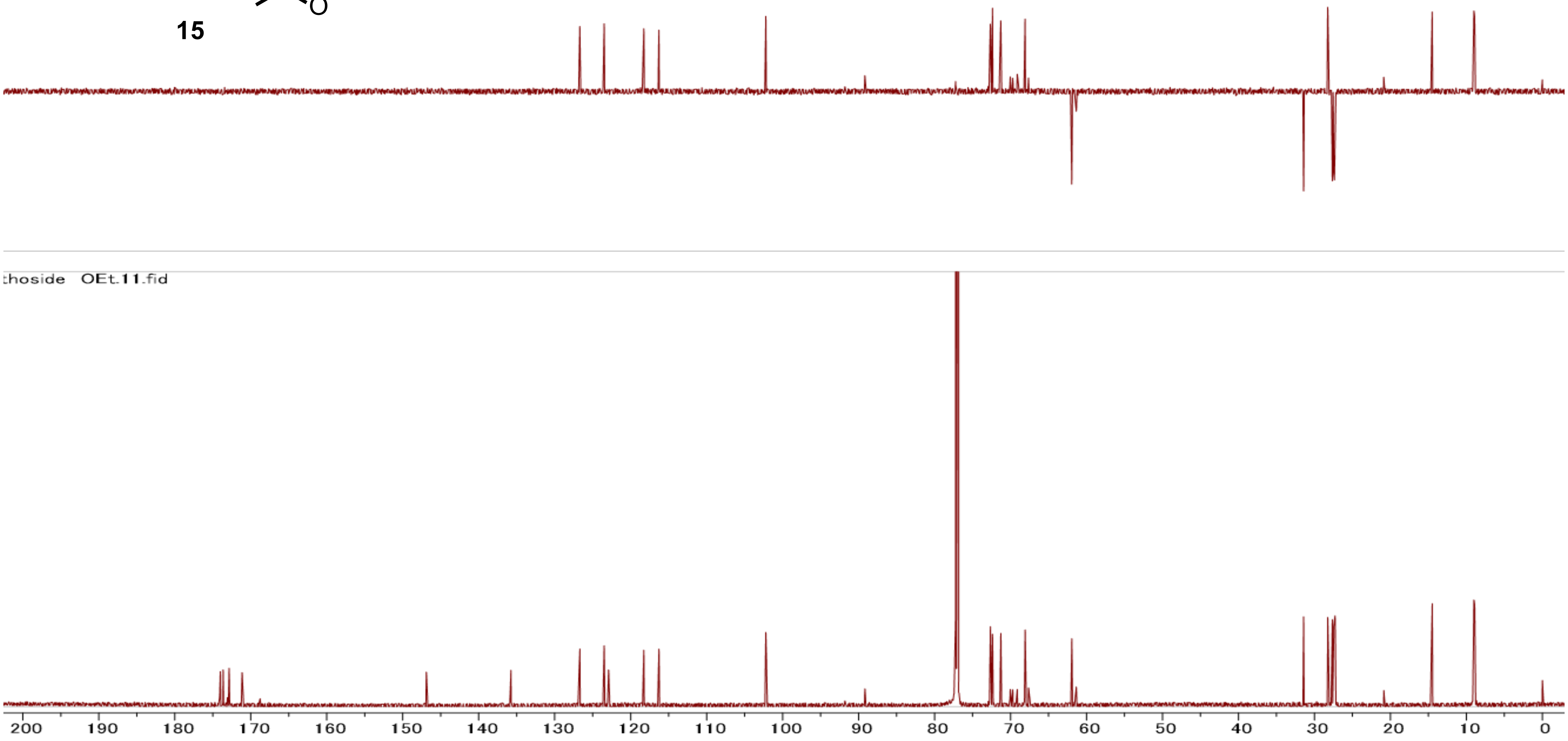

^{1}H-NMR (800 MHz, CD_3OD) of 2-(Ethylthio)-1*H*-indol-3-yl *β*-D-glucopyranoside (8g).

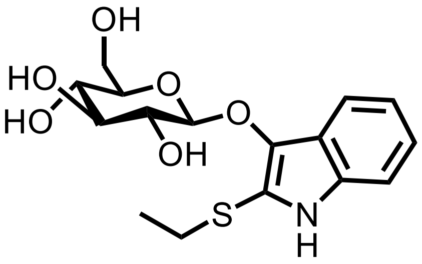

8g

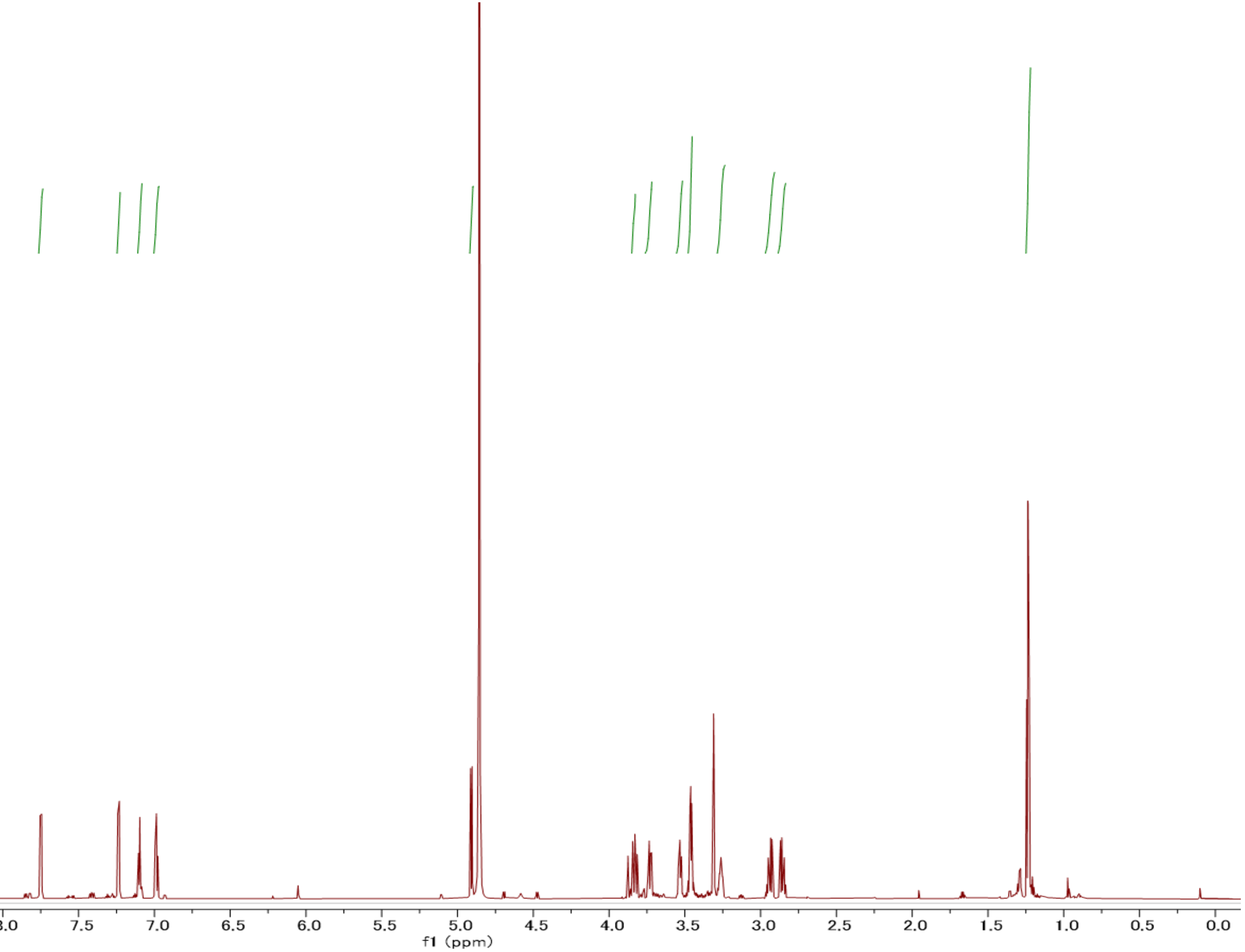

^{13}C-NMR (200 MHz, CD_3OD) of 2-(Ethylthio)-1*H*-indol-3-yl *β*-D-glucopyranoside (8g).

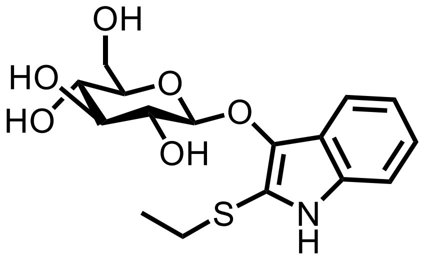

8g

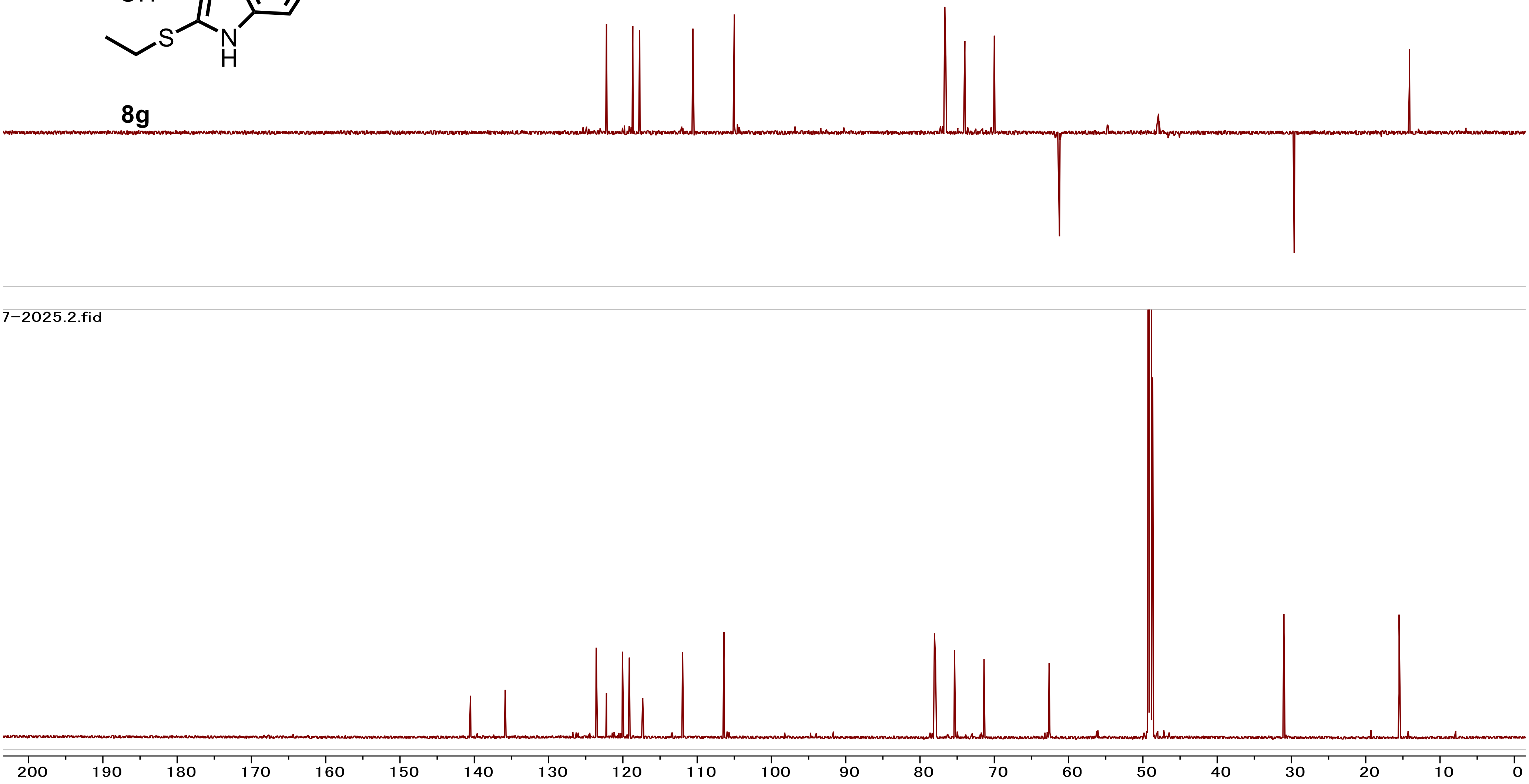

^{1}H-NMR (800 MHz, CD_3OD) of 3-(*β*-D-Glactopyranosyloxy)-1*H*-indol-2-yl 1-thio-*β*-D-glucopyranoside (9b).

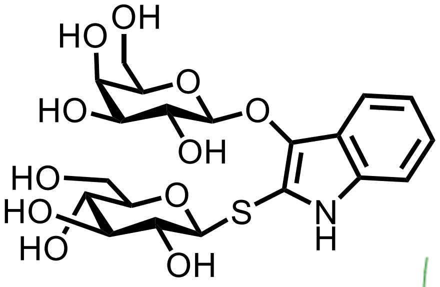

9b

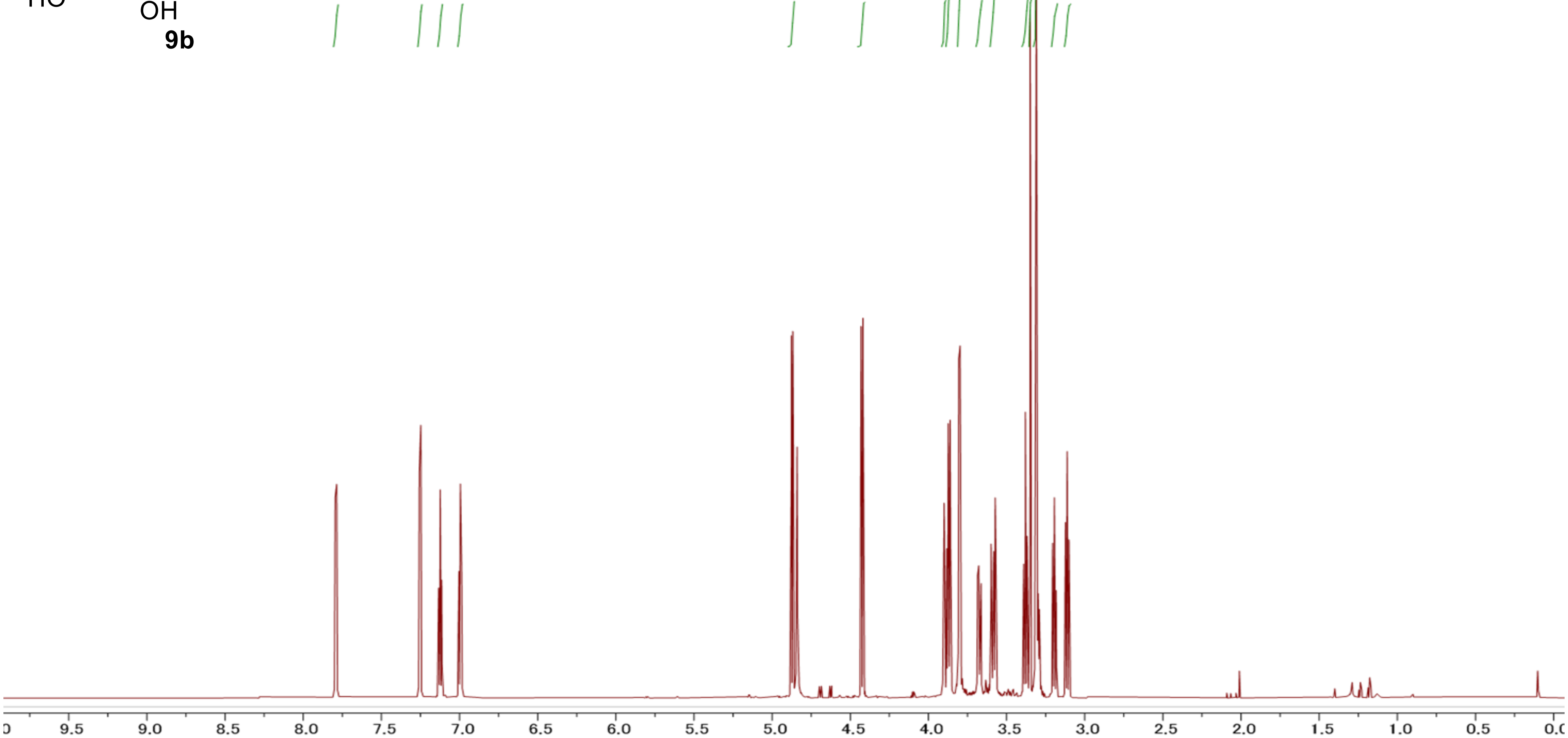

^{13}C-NMR (200 MHz, CD_3OD) of 3-(*β*-D-Glactopyranosyloxy)-1*H*-indol-2-yl 1-thio-*β*-D-glucopyranoside (9b).

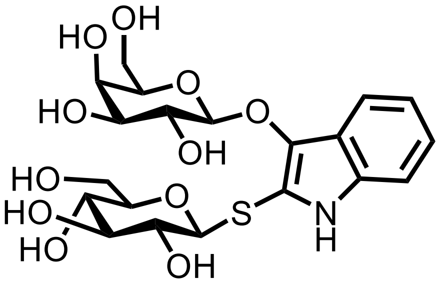

9b

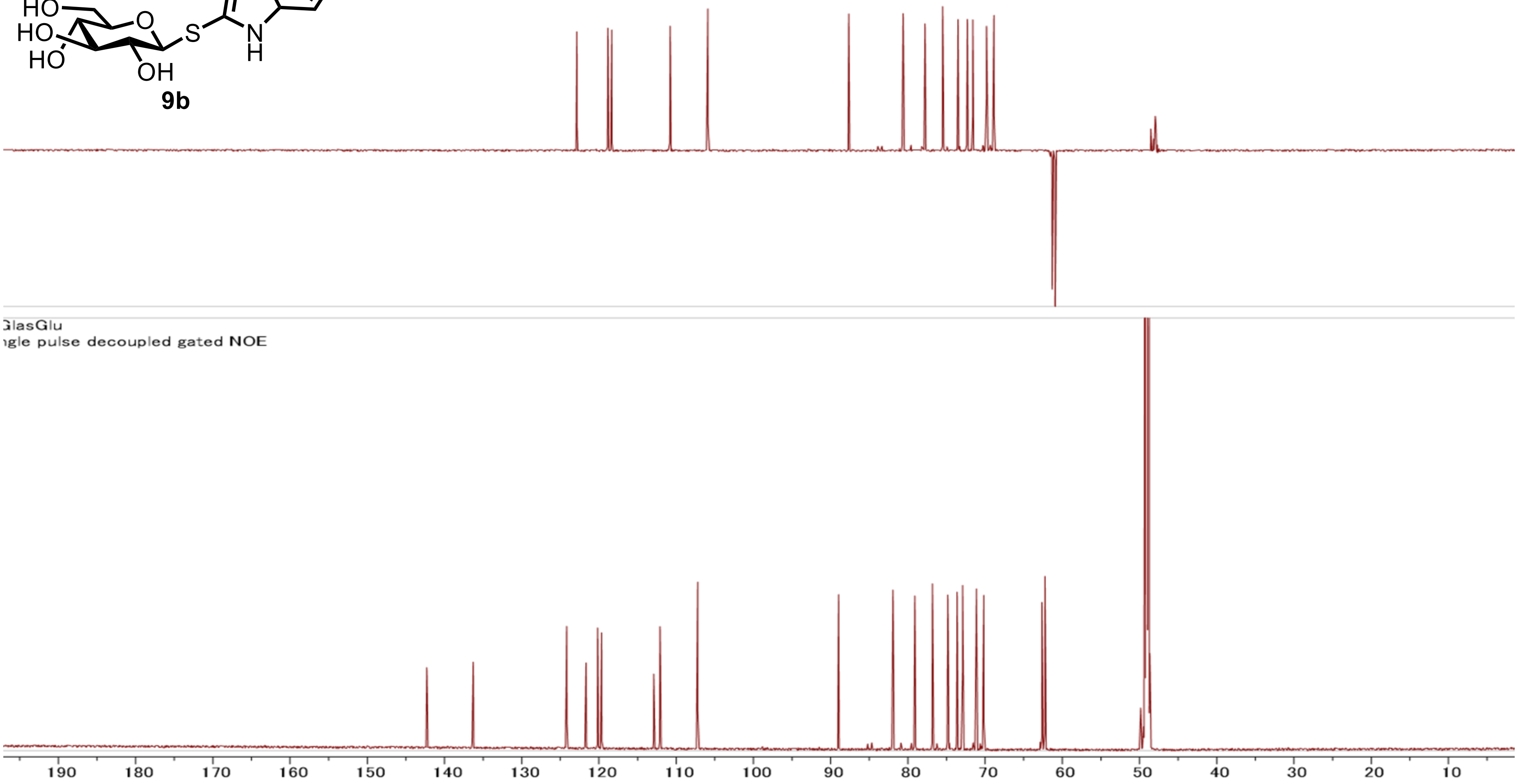

^{1}H-NMR (800 MHz, CD_3OD) of 3-(*β*-D-Xylopyranosyloxy)-1*H*-indol-2-yl 1-thio-*β*-D-glucopyranoside (9d).

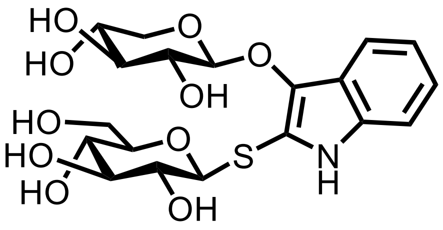

9d

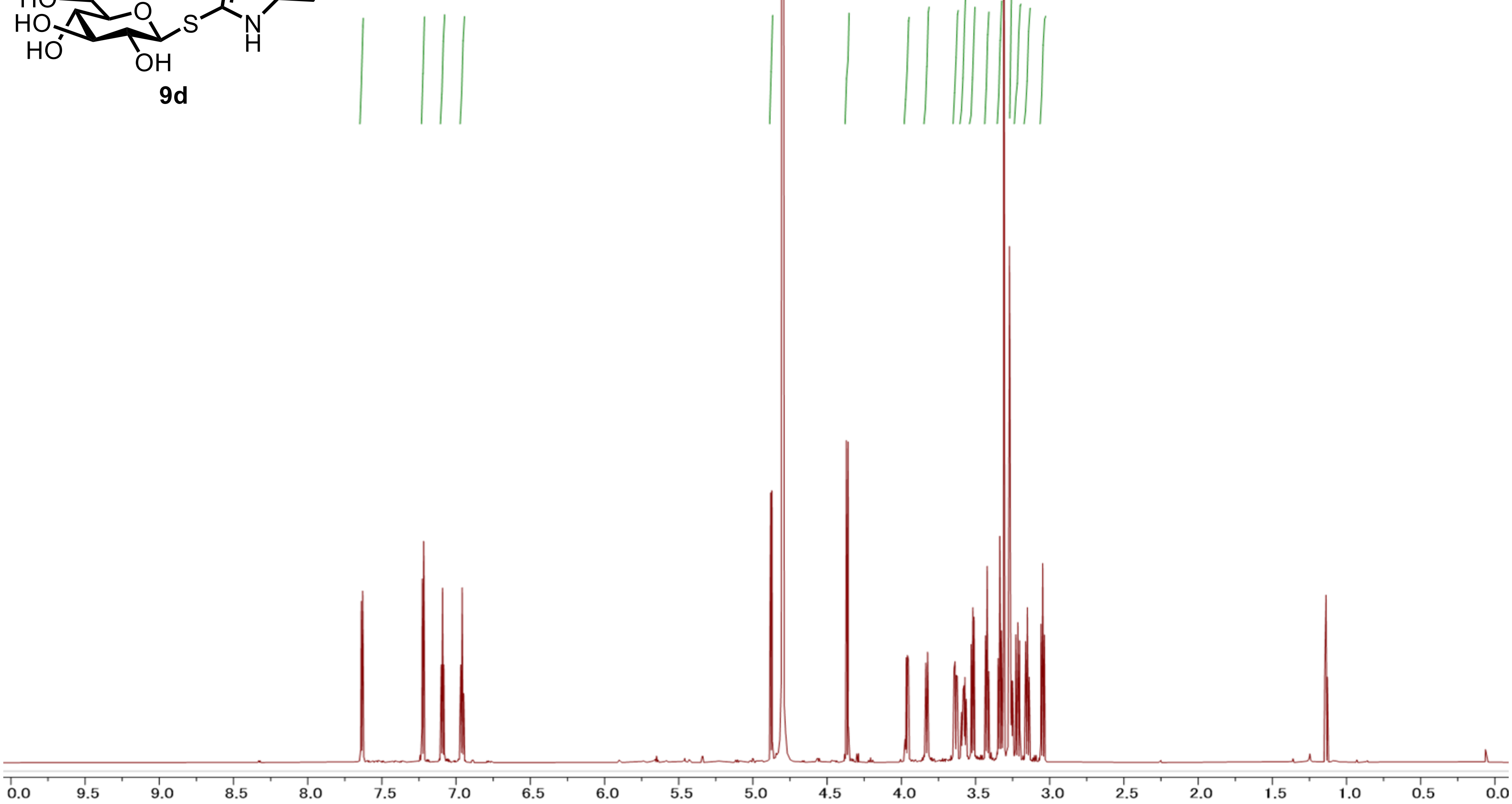

^{13}C-NMR (200 MHz, CD$_3$OD) of 3-(*β*-D-Xylopyranosyloxy)-1*H*-indol-2-yl 1-thio-*β*-D-glucopyranoside (9d).

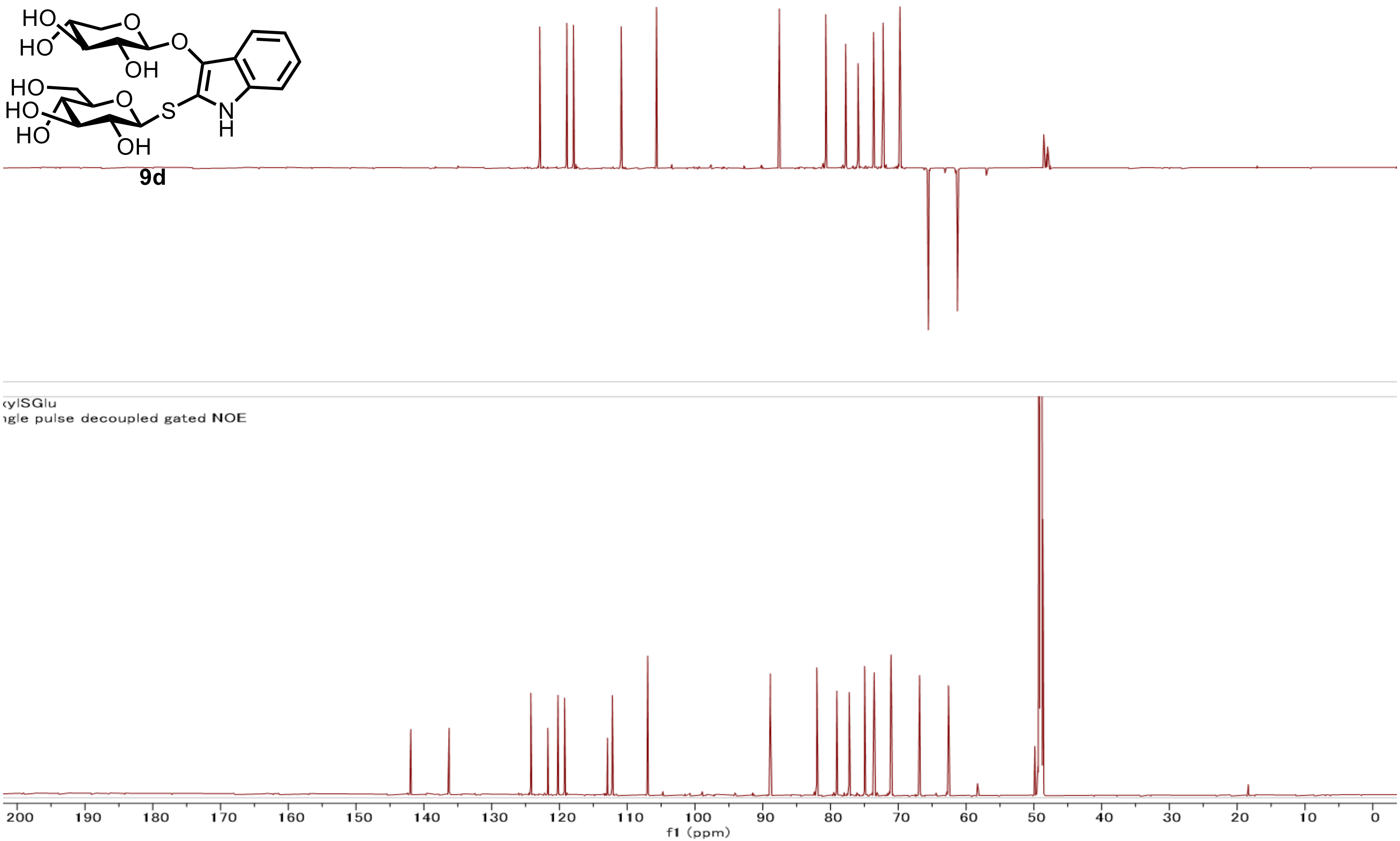

^{1}H-NMR (800 MHz, D_2O) of 3-(*β*-L-Arabinopyranosyloxy)-1*H*-indol-2-yl 1-thio-*β*-D-glucopyranoside (9e).

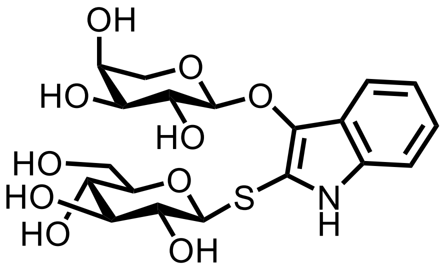

9e

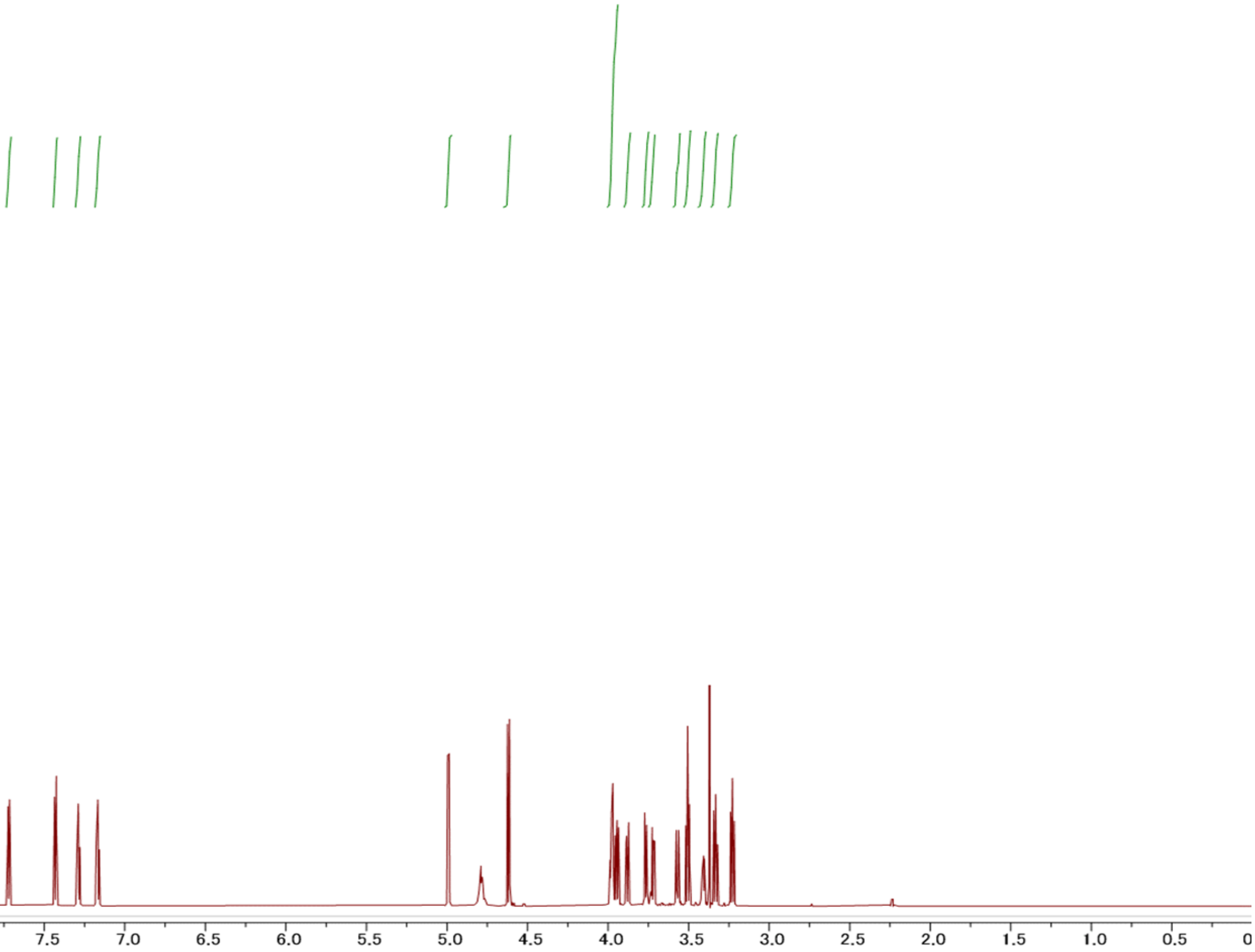

^{13}C-NMR (200 MHz, D$_2$O) of 3-(*β*-L-Arabinopyranosyloxy)-1*H*-indol-2-yl 1-thio-*β*-D-glucopyranoside (9e).

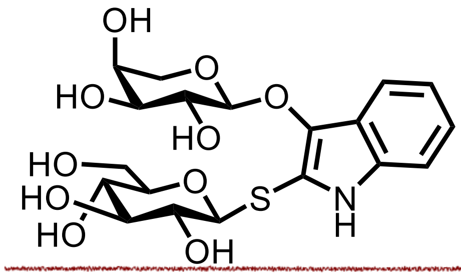

9e

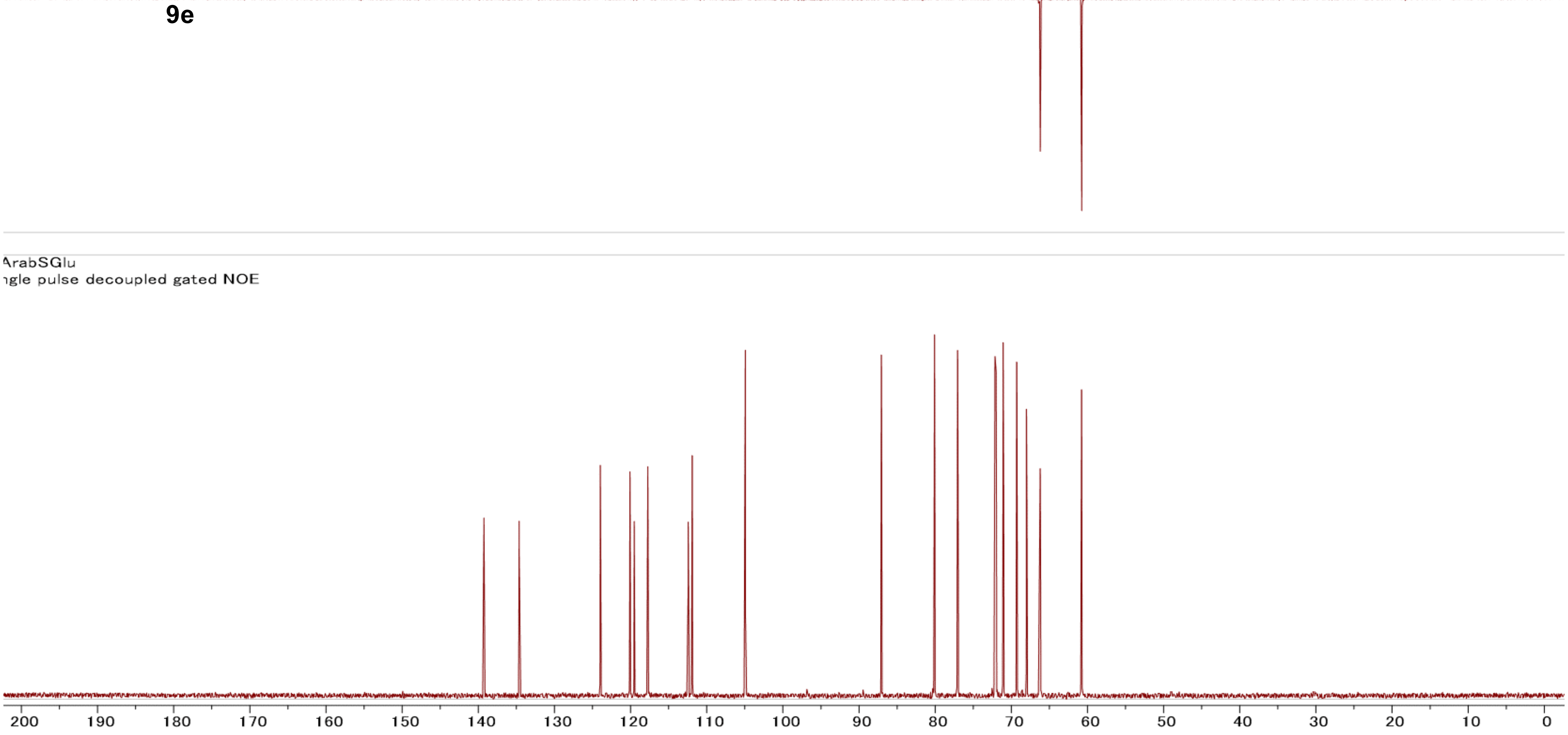

^{1}H-NMR (800 MHz, D_2O) of 3-(*β*-D-Maltopyranosyloxy)-1*H*-indol-2-yl 1-thio-*β*-D-glucopyranoside (9f).

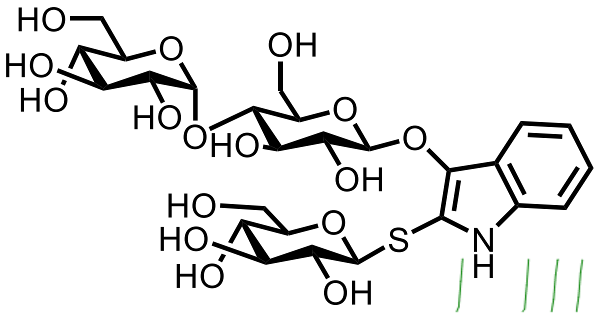

9f

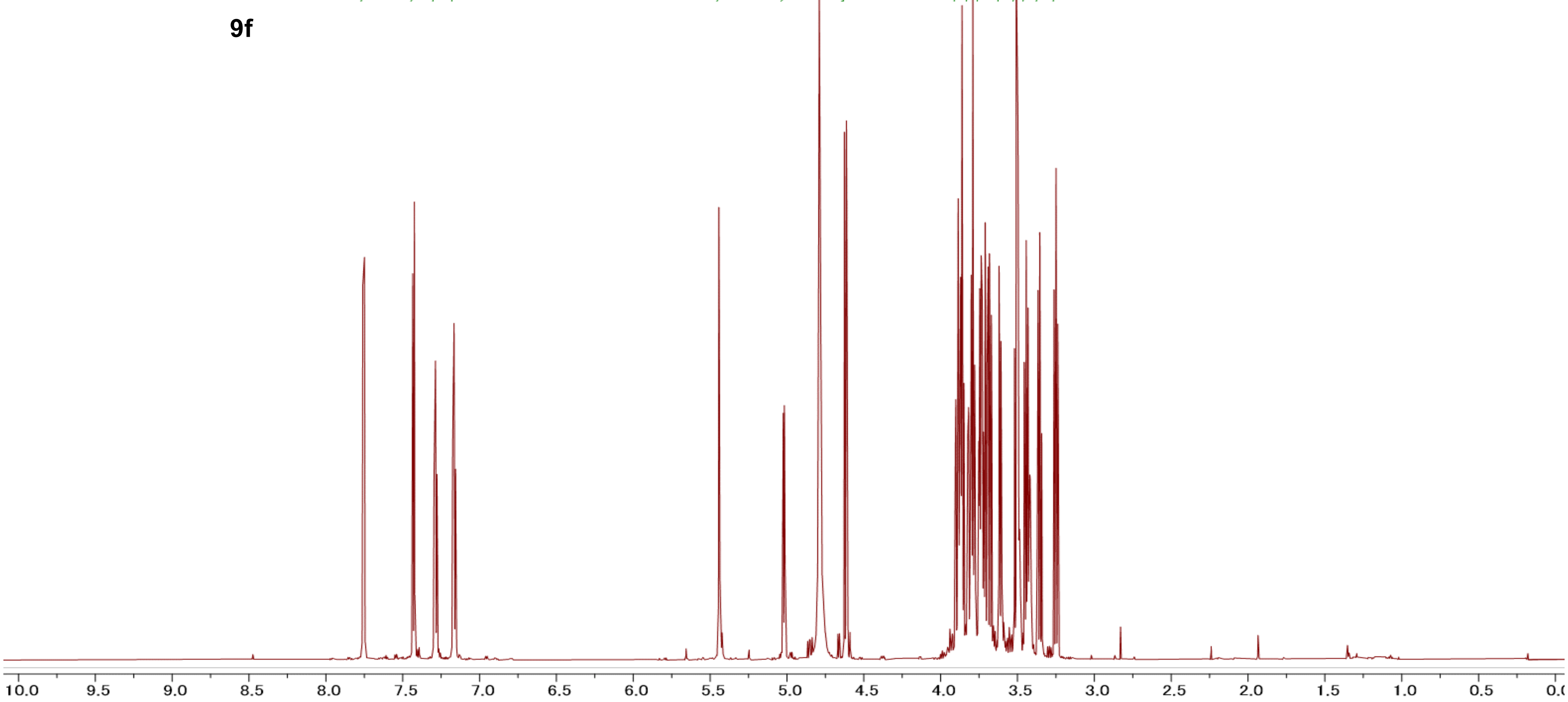

^{13}C-NMR (200 MHz, D_2O) of 3-(*β*-D-Maltopyranosyloxy)-1*H*-indol-2-yl 1-thio-*β*-D-glucopyranoside (9f).

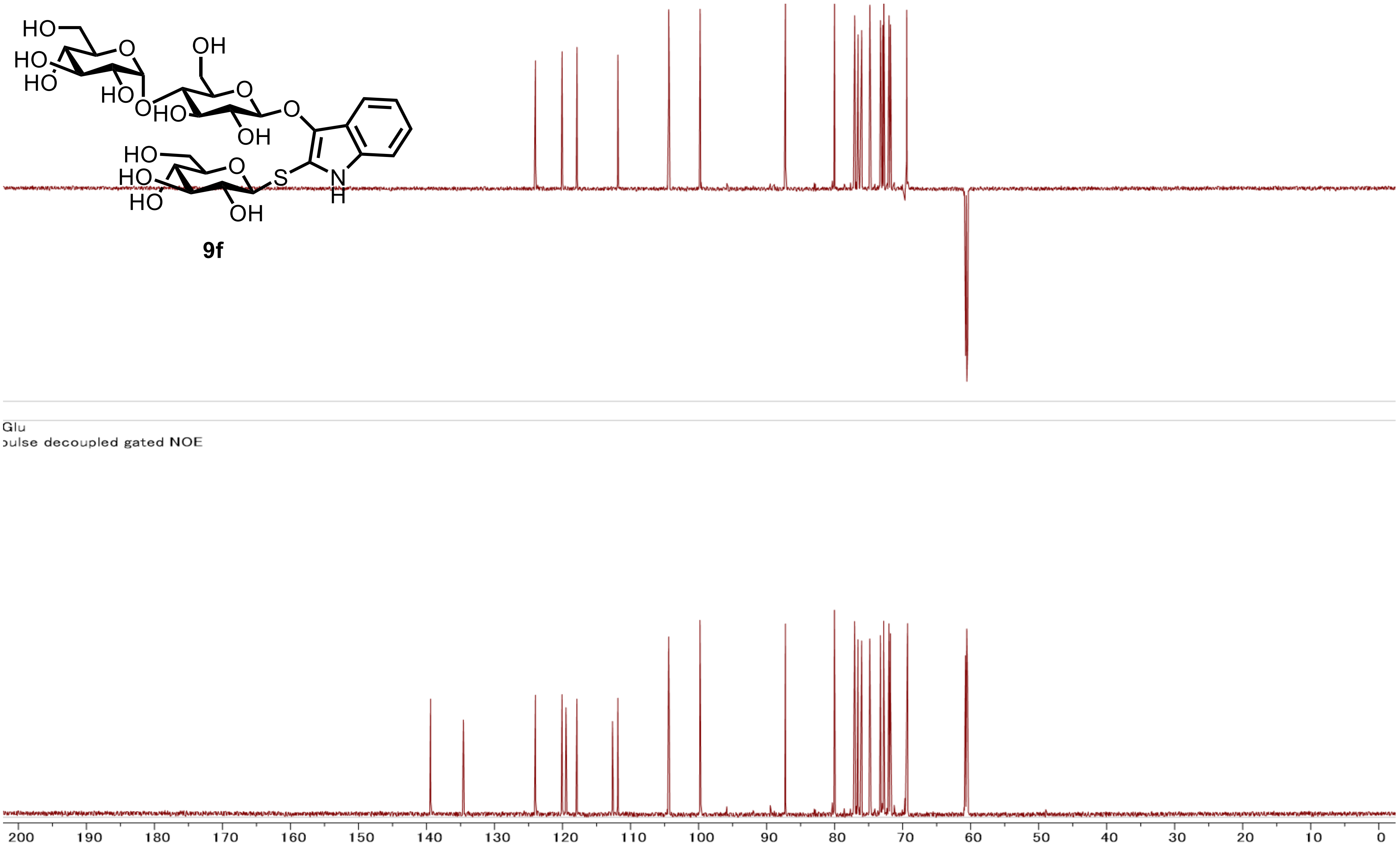

^{1}H-NMR (800 MHz, CDCl$_3$) of *N*-acetyl-3-(2,3,4,6-tetra-*O*-acetyl-β-D-glucopyranosyloxy)-1*H*-indol-2-yl 2,3,4,6-tetra-*O*-acetyl-1-thio-β-D-glucopyranoside (10a).

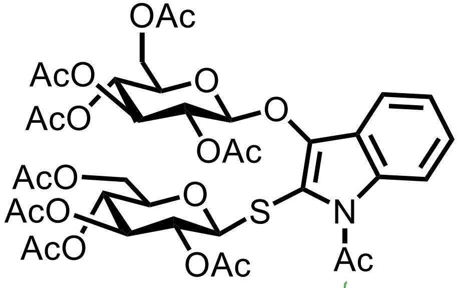

10a

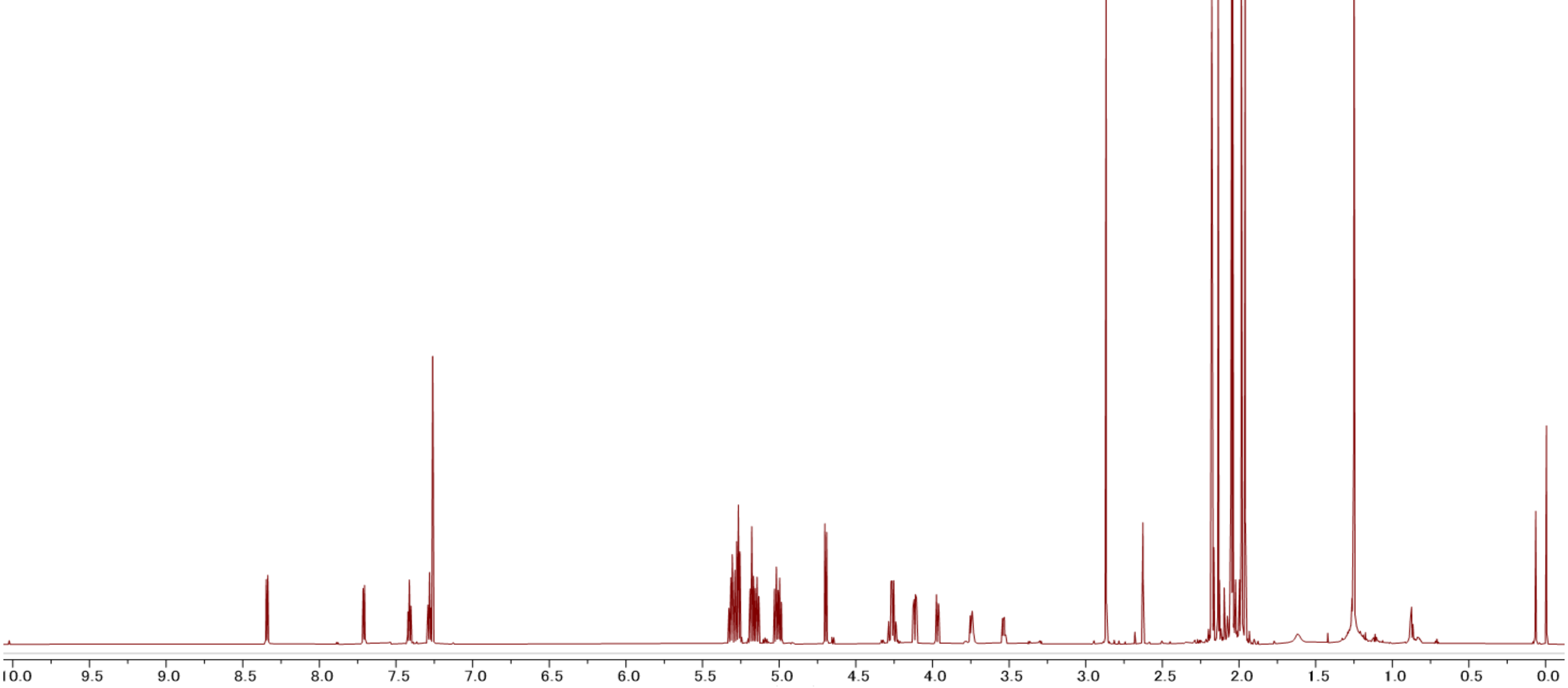

^{13}C-NMR (200 MHz, CDCl$_3$) of *N*-acetyl-3-(2,3,4,6-tetra-*O*-acetyl-β-D-glucopyranosyloxy)-1*H*-indol-2-yl 2,3,4,6-tetra-*O*-acetyl-1-thio-β-D-glucopyranoside (10a).

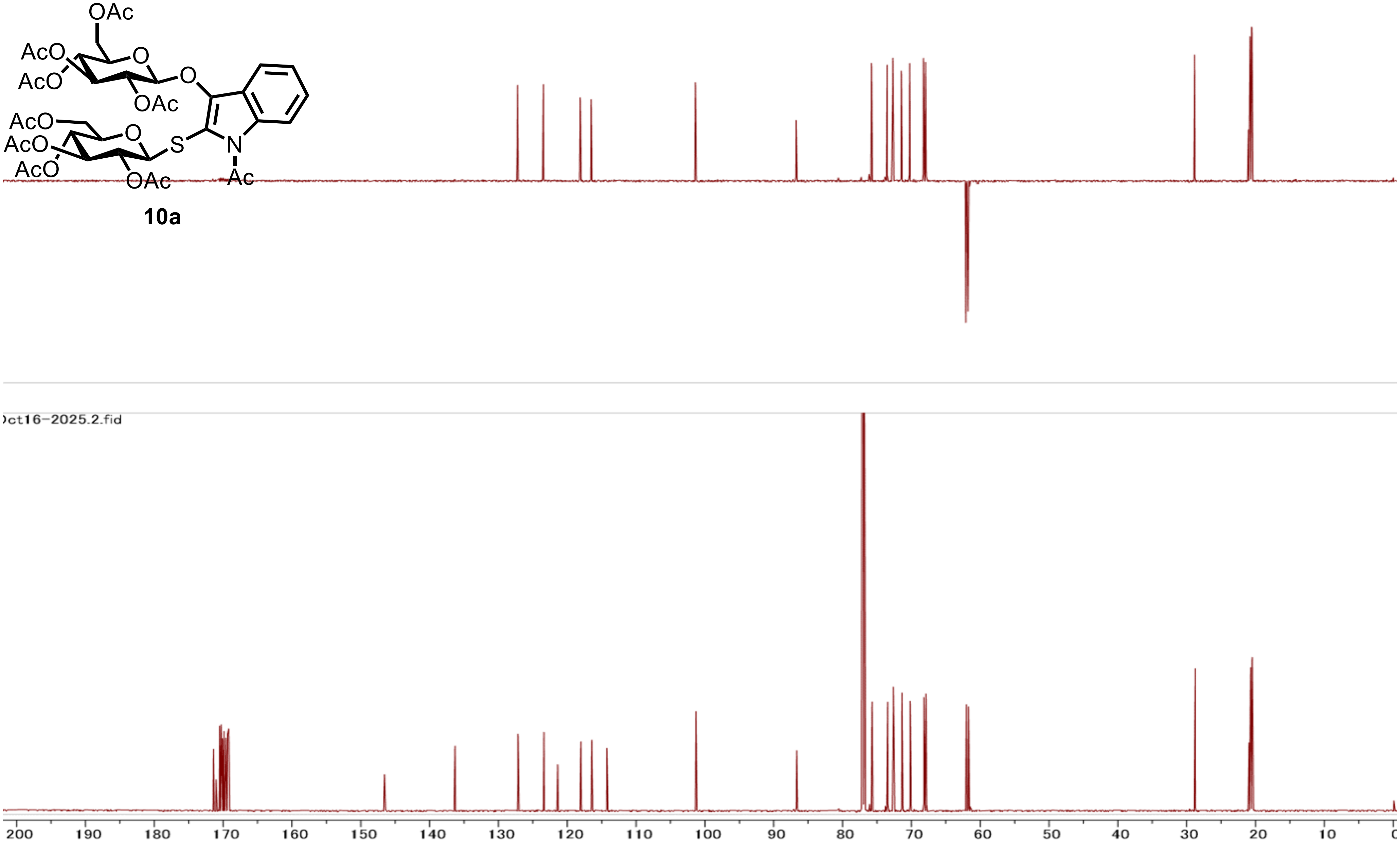

^{1}H-NMR (800 MHz, CDCl3) of 3-(2,3,4,6-Tetra-*O*-propionyl-*β*-D-glucopyranosyloxy)-1*H*-indol-2-yl 2,3,4,6-tetra-*O*-propionyl-1-thio-*β*-D-glucopyranoside (10b).

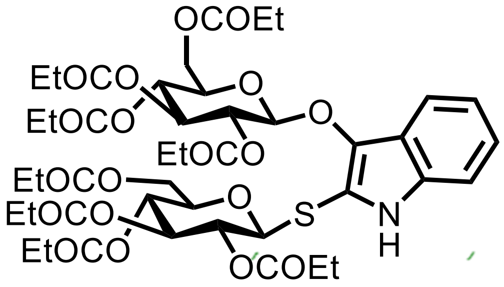

10b

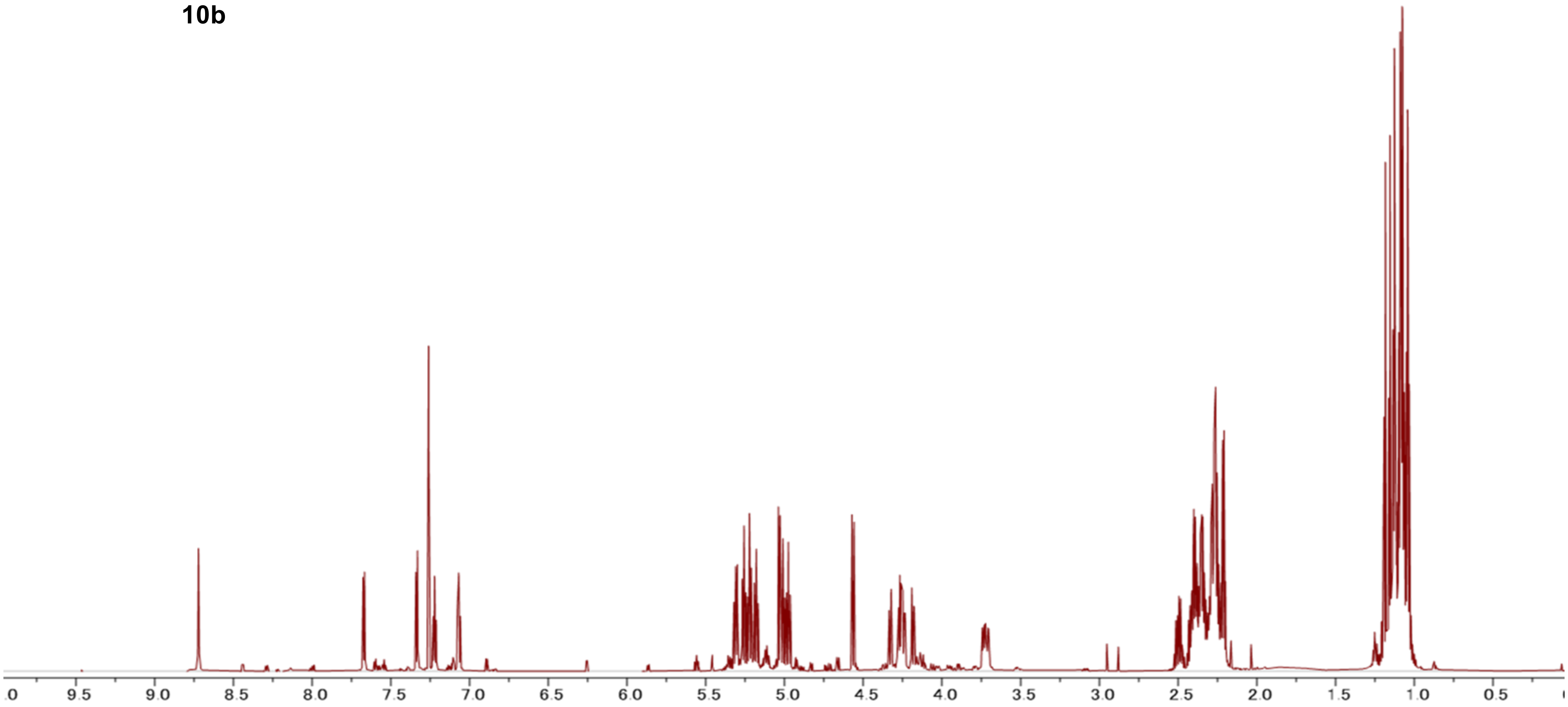

^{13}C-NMR (200 MHz, $CDCl_3$) of 3-(2,3,4,6-Tetra-*O*-propionyl-*β*-D-glucopyranosyloxy)-1*H*-indol-2-yl 2,3,4,6-tetra-*O*-propionyl-1-thio-*β*-D-glucopyranoside (10b).

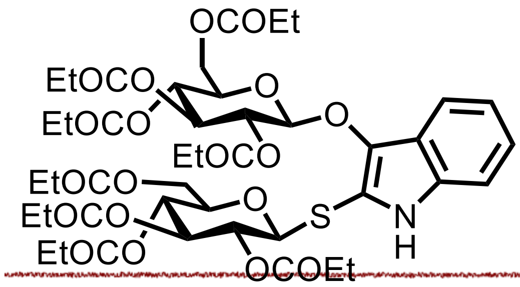

10b

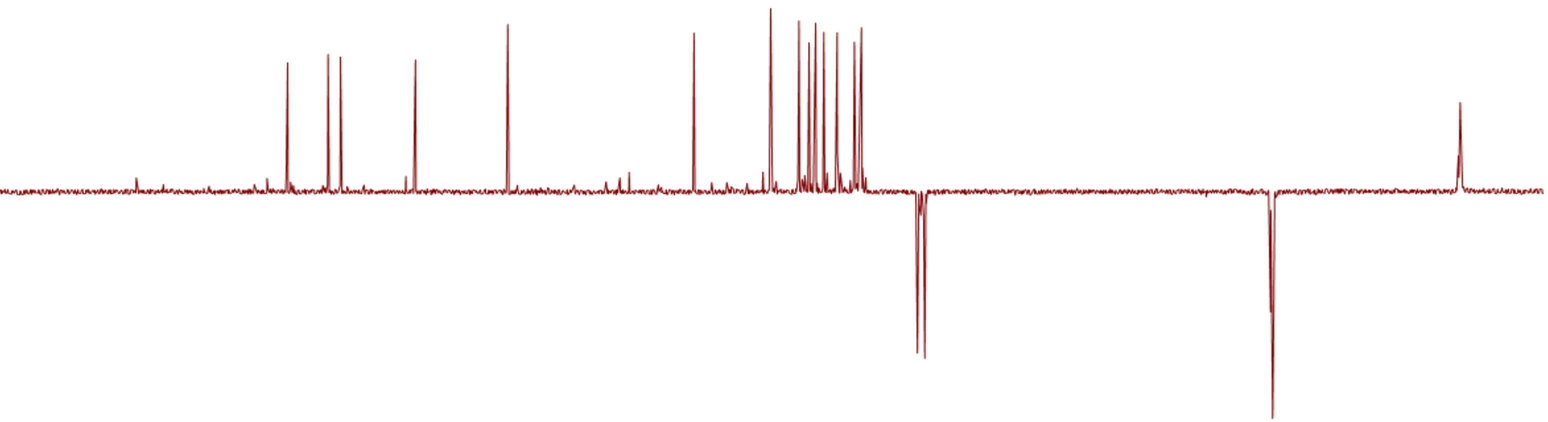

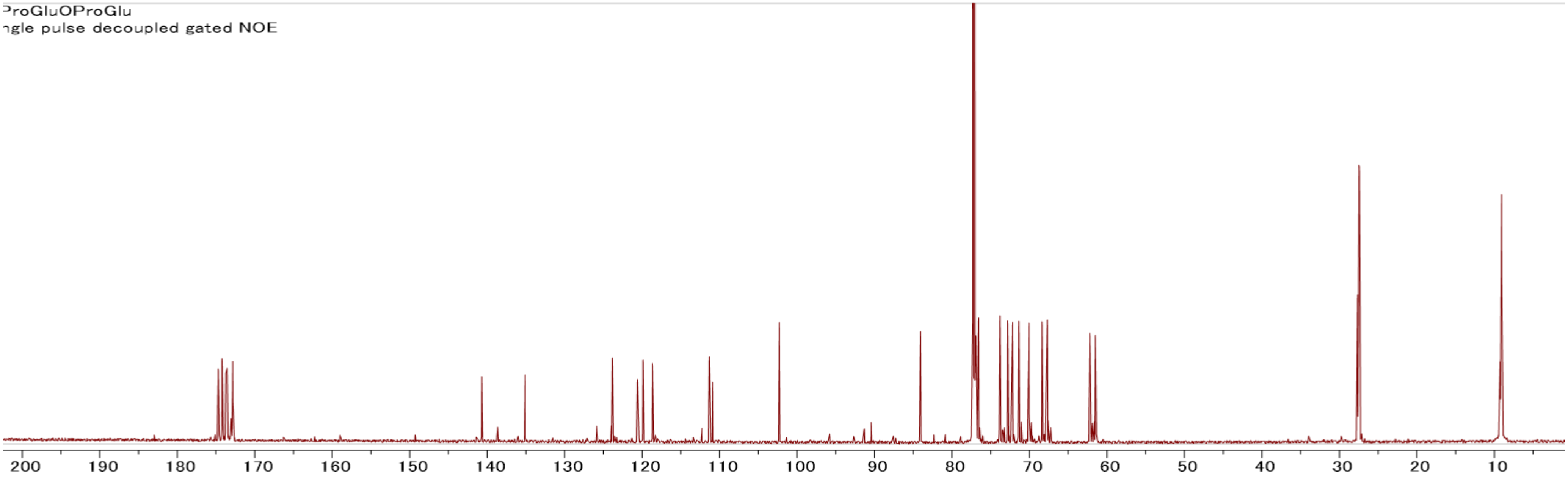

^{1}H-NMR (800 MHz, $CDCl_3$) of 2-Acetyloxy-2,3-dihydro-1-benzofuran-3-one (16a).

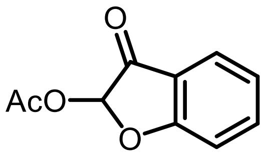

16a

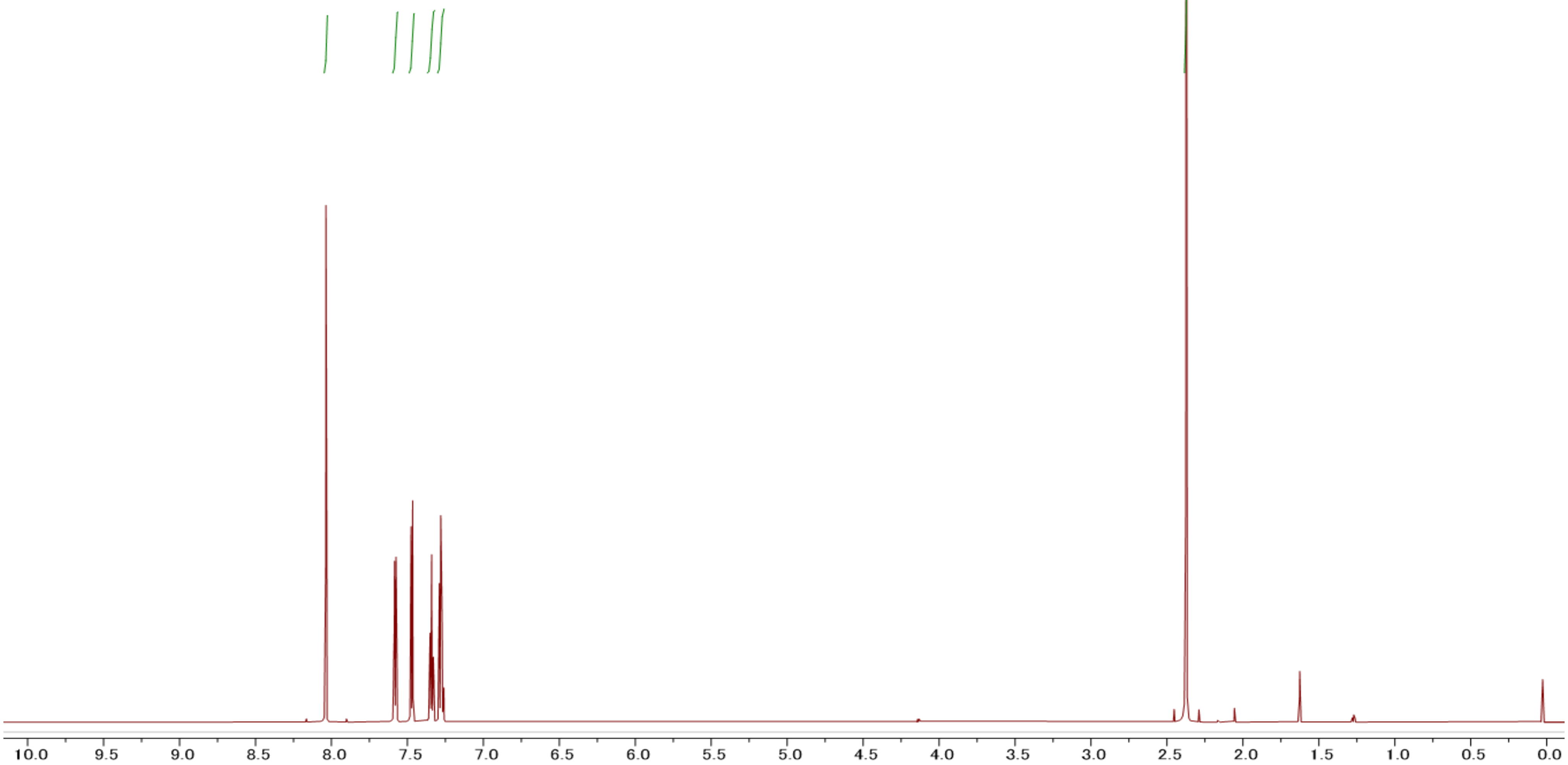

^{13}C-NMR (200 MHz, $CDCl_3$) of 2-Acetyloxy-2,3-dihydro-1-benzofuran-3-one (16a).

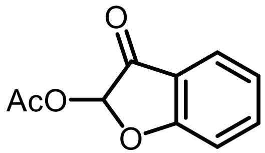

16a

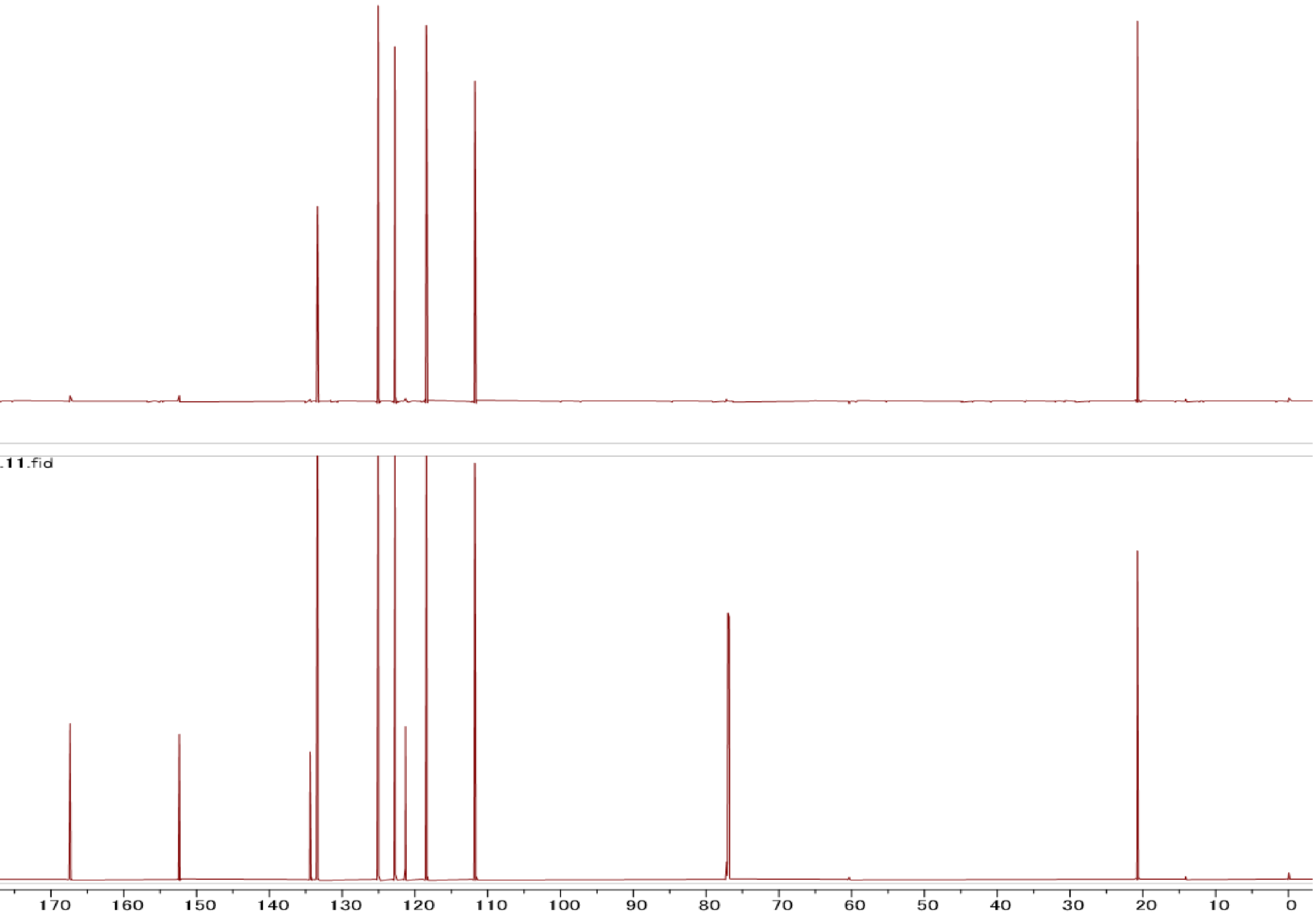

^{1}H-NMR (800 MHz, $CDCl_3$) of 2,3-Dihydro-1-benzofuran-3-one (18).

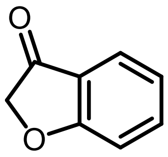

18

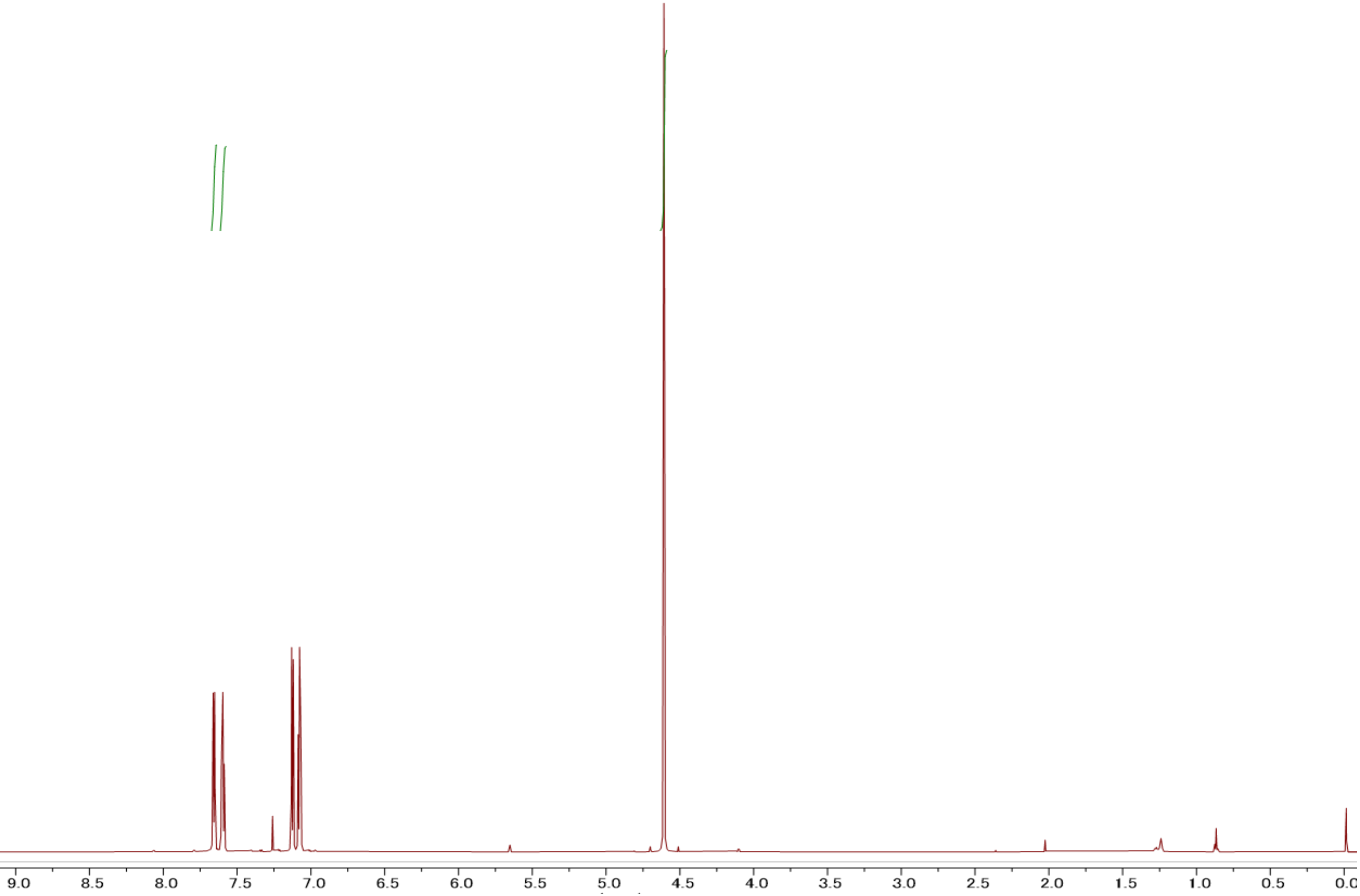

^{13}C-NMR (200 MHz, $CDCl_3$) of 2,3-Dihydro-1-benzofuran-3-one (18).

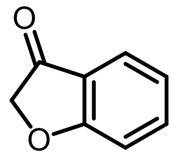

18

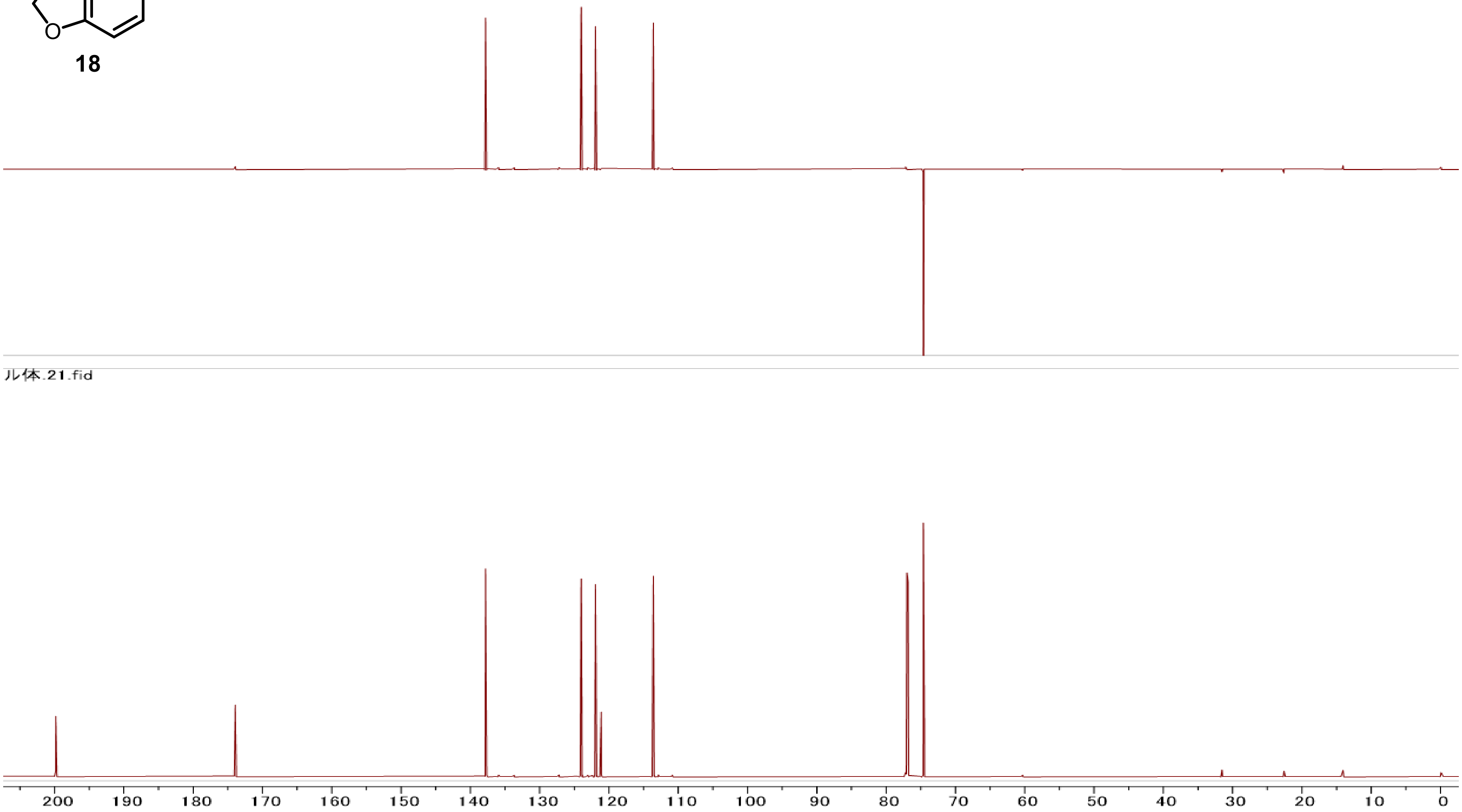

^{1}H-NMR (800 MHz, $CDCl_3$) of 2-Bromo-2,3-dihydro-1-benzofuran-3-one (16b).

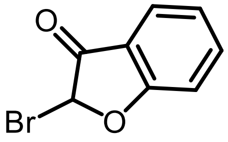

16b

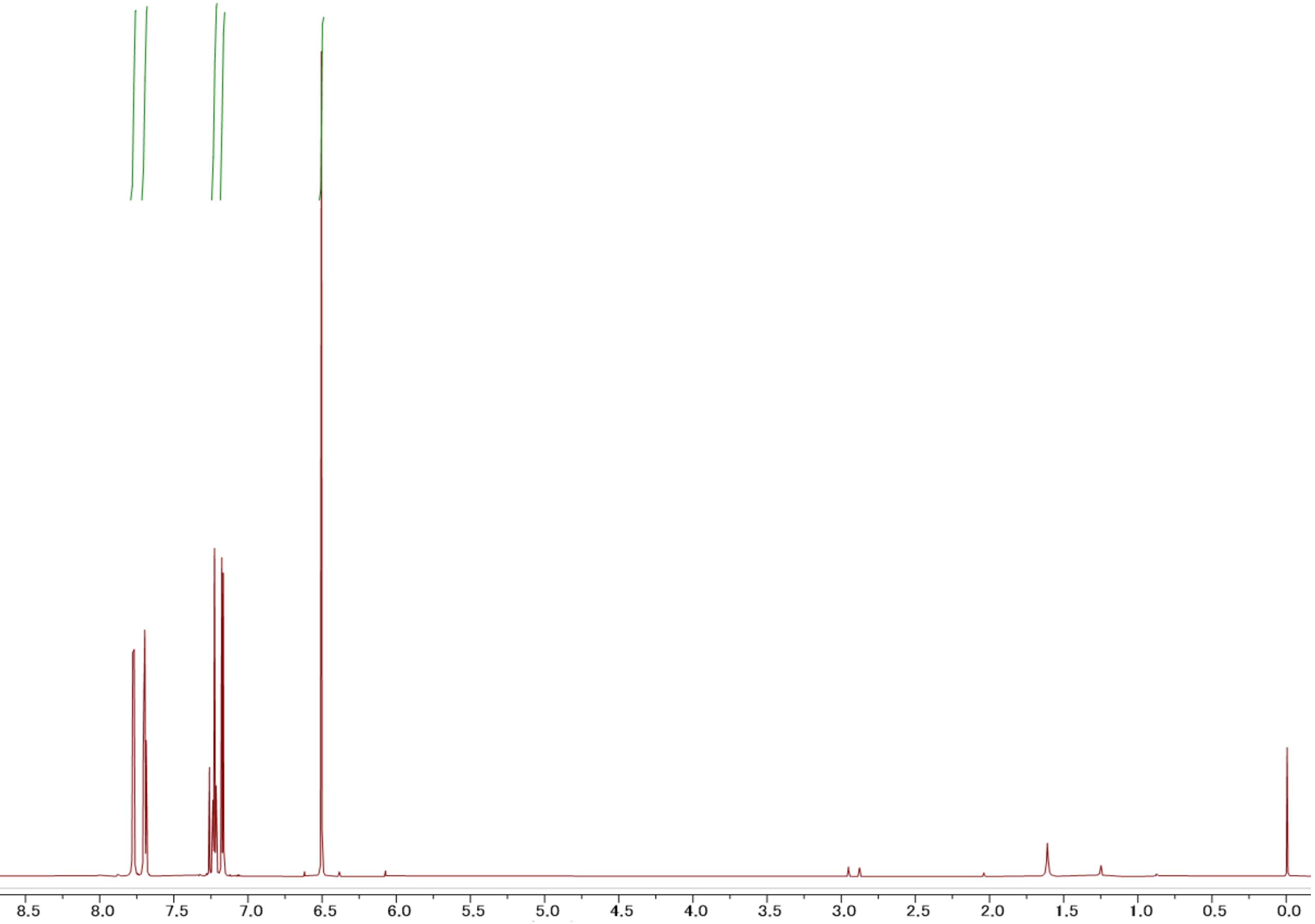

^{13}C-NMR (200 MHz, $CDCl_3$) of 2-Bromo-2,3-dihydro-1-benzofuran-3-one (16b).

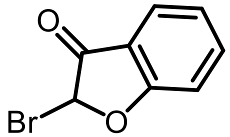

16b

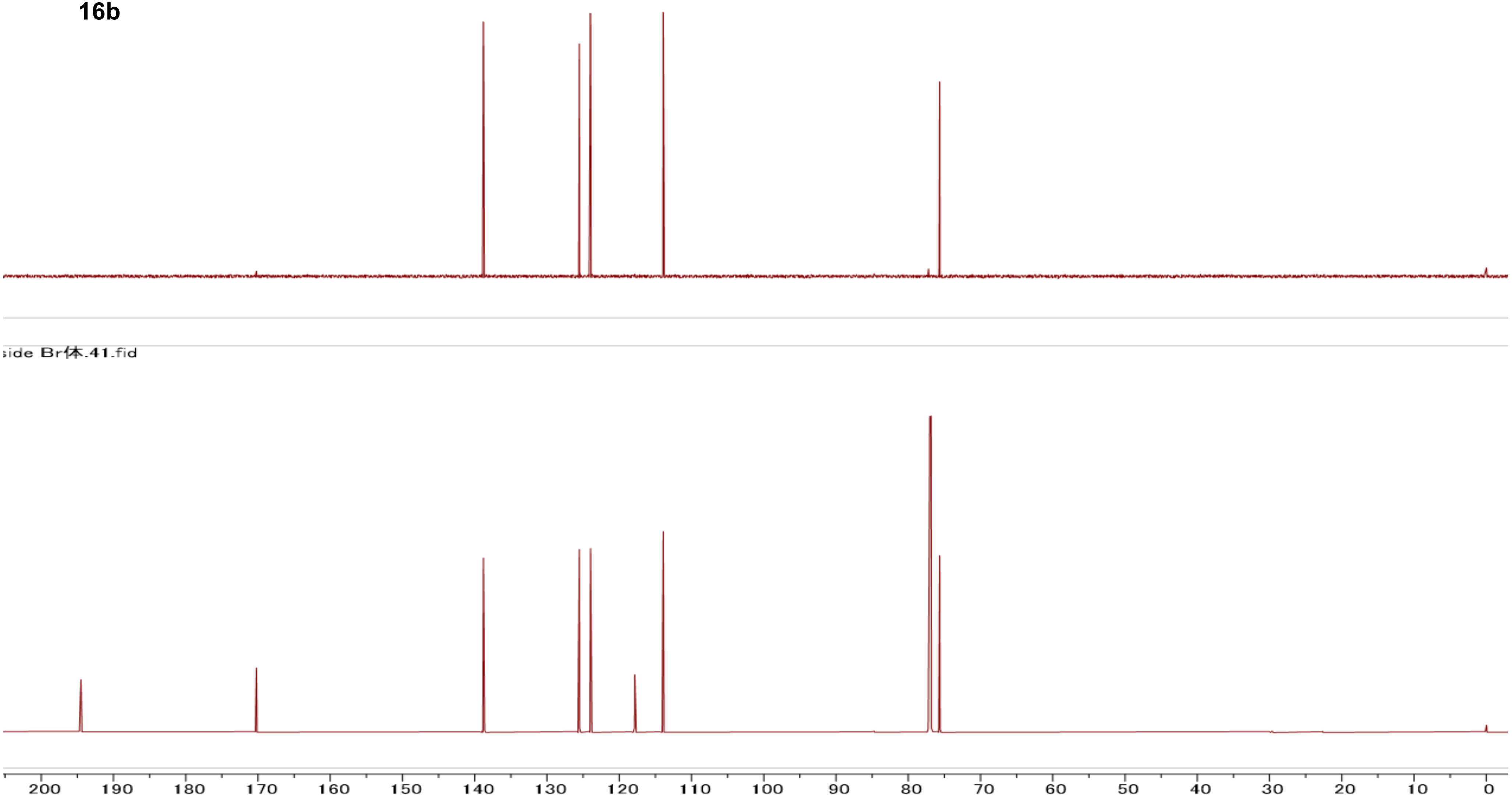

^{1}H-NMR (800 MHz, CDCl3) of 3-(2,3,4,6-Tetra-*O*-propionyl-*β*-D-glucopyranosyloxy)-benzofura-2-yl 2,3,4,6-tetra-*O*-acetyl-1-thio-*β*-D-glucopyranoside (20).

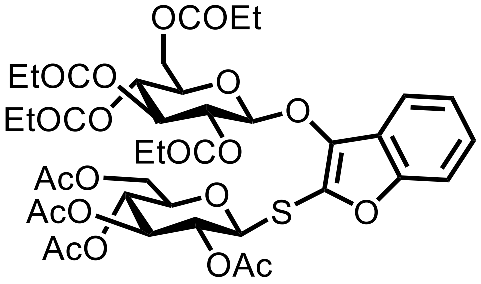

20

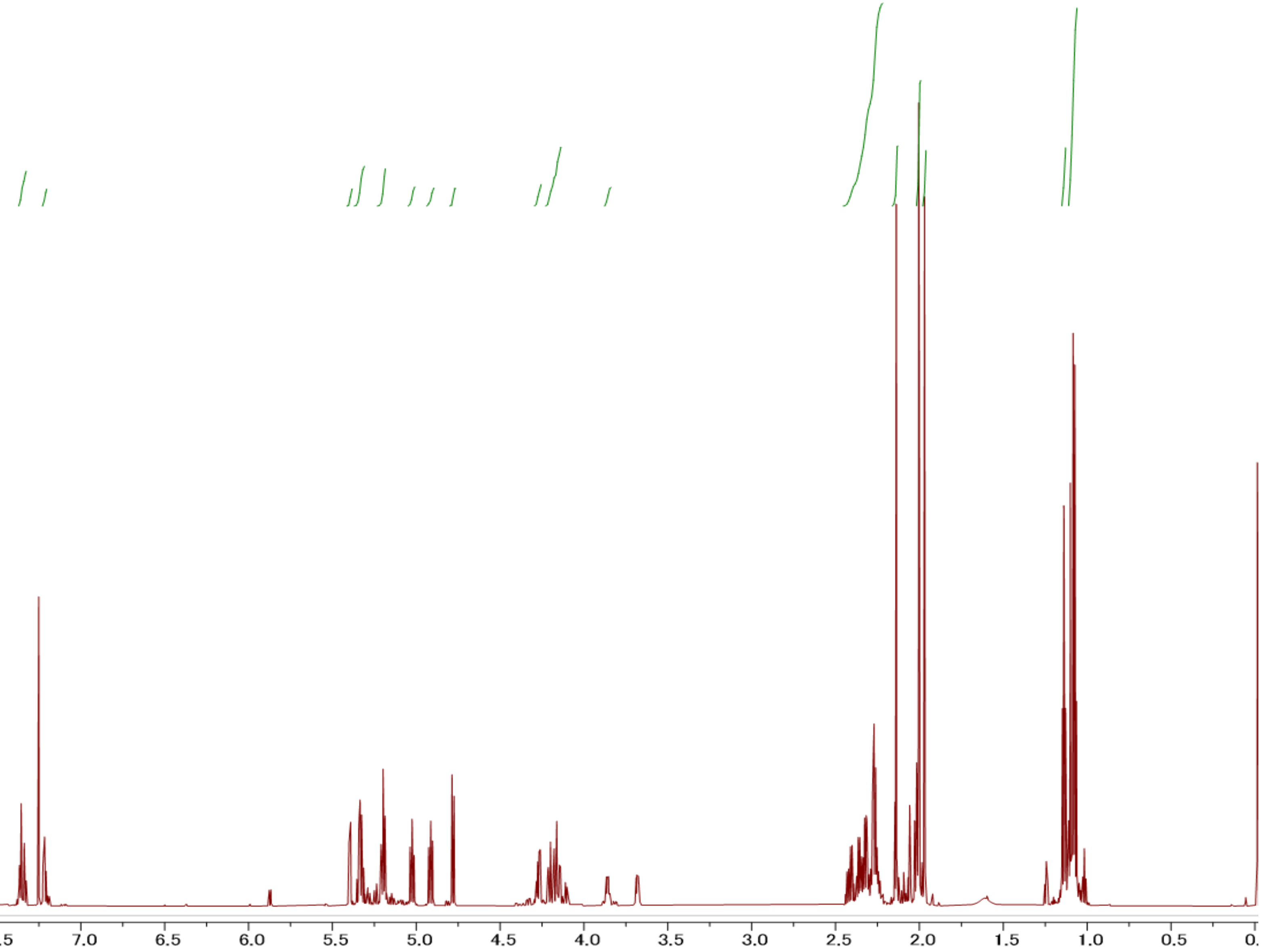

^{13}C-NMR (200 MHz, CDCl3) of 3-(2,3,4,6-Tetra-*O*-propionyl-*β*-D-glucopyranosyloxy)-benzofura-2-yl 2,3,4,6-tetra-*O*-acetyl-1-thio-*β*-D-glucopyranoside (20).

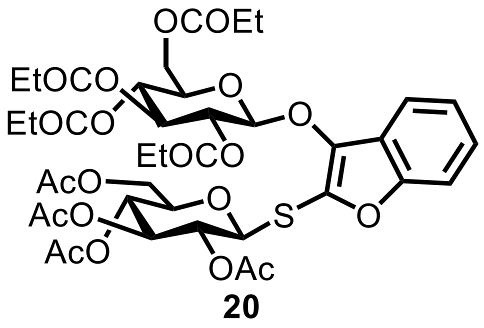

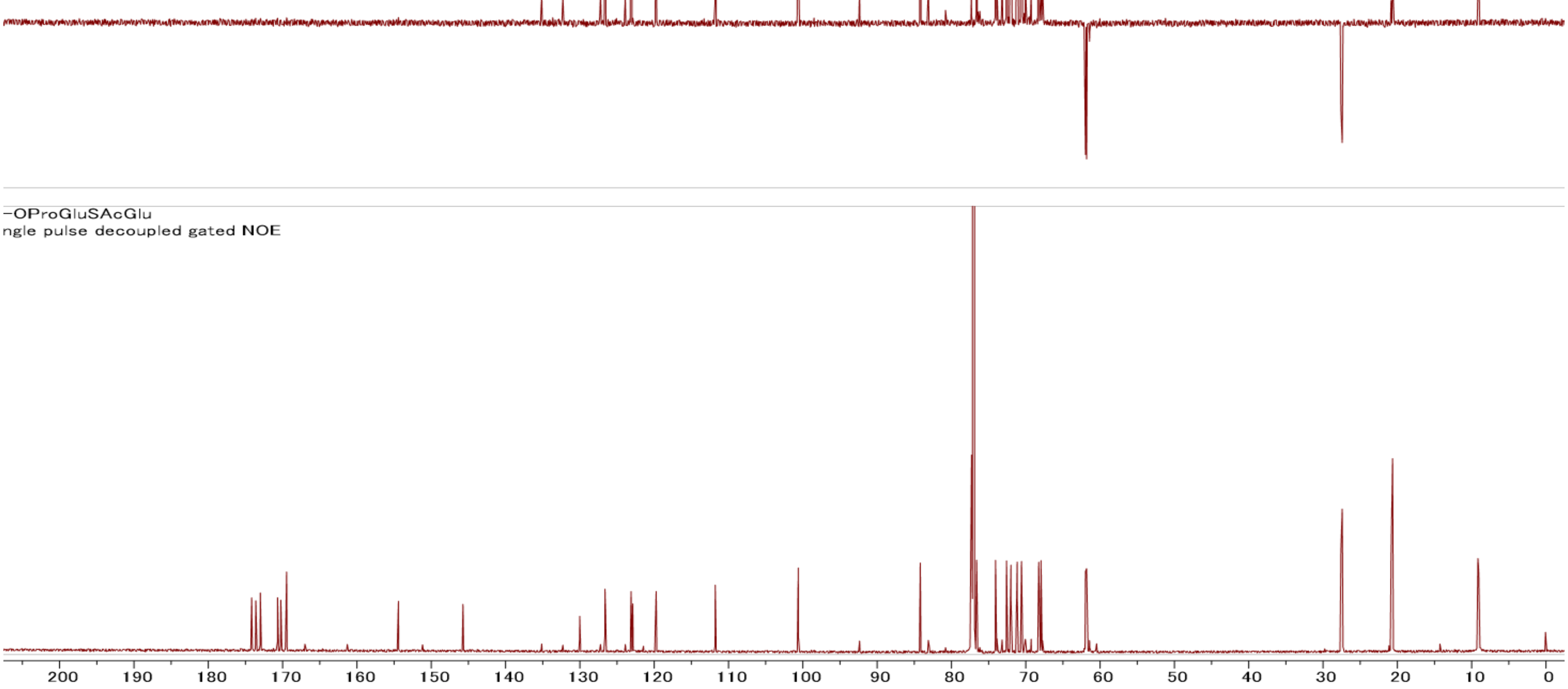

^{1}H-NMR (800 MHz, DMSO-*d6*) of 3-(*β*-D-Glucopyranosyloxy)benzofuran-2-yl 1-thio-*β*-D-glucopyranoside (11).

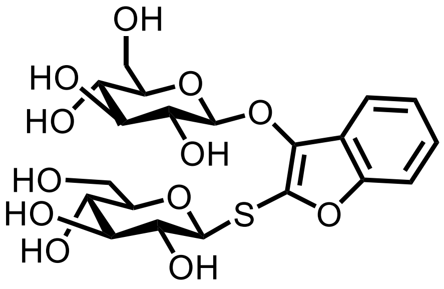

11

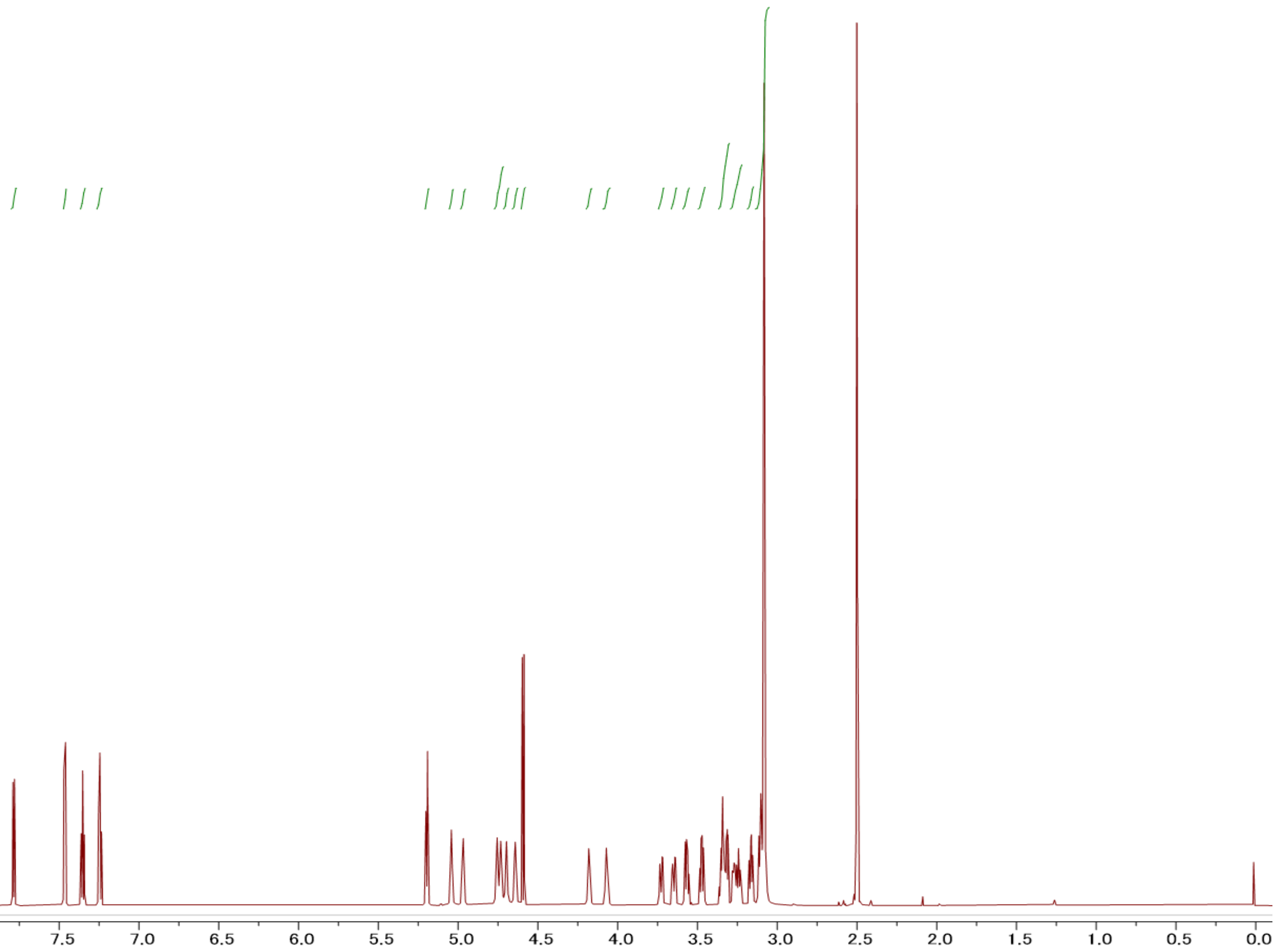

^{13}C-NMR (200 MHz, DMSO-*d6*) of 3-(*β*-D-Glucopyranosyloxy)benzofuran-2-yl 1-thio-*β*-D-glucopyranoside (11).

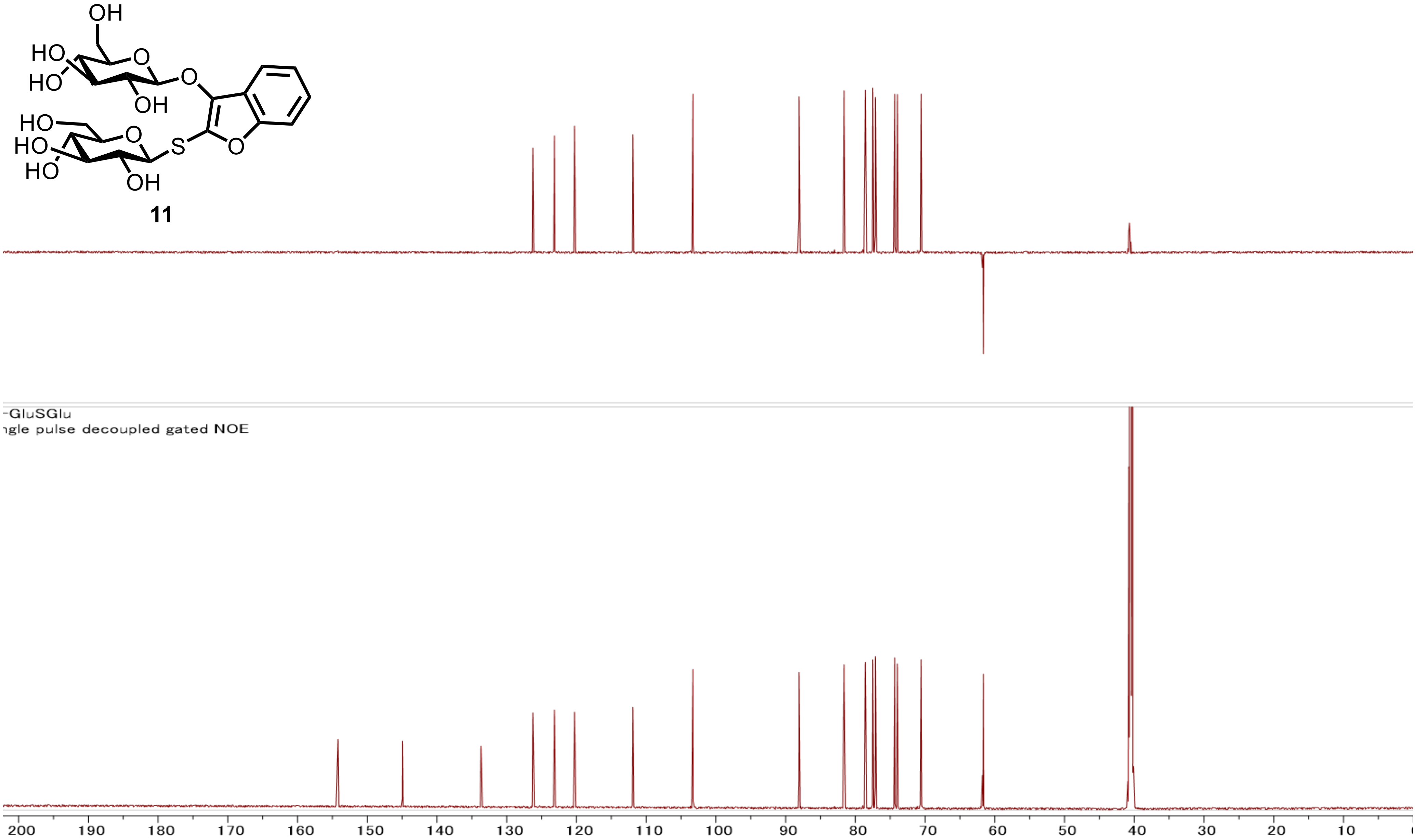

Supplement: MD-017-D6MD00273K-s001 [file MD-017-D6MD00273K-s001.pdf]
